# Enabling metallic behaviour in two-dimensional superlattice of semiconductor colloidal quantum dots

**DOI:** 10.1038/s41467-023-38216-y

**Published:** 2023-05-26

**Authors:** Ricky Dwi Septianto, Retno Miranti, Tomoka Kikitsu, Takaaki Hikima, Daisuke Hashizume, Nobuhiro Matsushita, Yoshihiro Iwasa, Satria Zulkarnaen Bisri

**Affiliations:** 1 RIKEN Center for Emergent Matter Science (CEMS), 2-1 Hirosawa, Wako, Saitama 351-0198 Japan; 2 Department of Materials Science and Engineering, Tokyo Institute of Technology, 2-12-1 Ookayama, Meguro, Tokyo, 152-8550 Japan; 3 RIKEN SPring-8 Center, 1-1-1 Kouto, Sayo, Hyogo 679-5198 Japan; 4 Quantum Phase Electronic Center (QPEC) and Department of Applied Physics, The University of Tokyo, 7-3-1 Hongo, Bunkyo-ku, Tokyo, 113-8656 Japan; 5 Department of Applied Physics and Chemical Engineering, Tokyo University of Agriculture and Technology, 2-24-16 Nakacho, Koganei, Tokyo, 184-8588 Japan

**Keywords:** Electronic devices, Quantum dots, Electronic properties and materials, Quantum dots

## Abstract

Semiconducting colloidal quantum dots and their assemblies exhibit superior optical properties owing to the quantum confinement effect. Thus, they are attracting tremendous interest from fundamental research to commercial applications. However, the electrical conducting properties remain detrimental predominantly due to the orientational disorder of quantum dots in the assembly. Here we report high conductivity and the consequent metallic behaviour of semiconducting colloidal quantum dots of lead sulphide. Precise facet orientation control to forming highly-ordered quasi-2-dimensional epitaxially-connected quantum dot superlattices is vital for high conductivity. The intrinsically high mobility over 10 cm^2^ V^−1^ s^−1^ and temperature-independent behaviour proved the high potential of semiconductor quantum dots for electrical conducting properties. Furthermore, the continuously tunable subband filling will enable quantum dot superlattices to be a future platform for emerging physical properties investigations, such as strongly correlated and topological states, as demonstrated in the moiré superlattices of twisted bilayer graphene.

## Abstract (web summary)

Charge carrier transport in colloidal quantum dot assemblies is slow due to hopping transport nature. Here, the authors report the demonstration of gate-tuned metallic state in epitaxially-connected quantum dot superlattices by minimizing disorders.

## Introduction

Assembling colloidal quantum dots (CQD) into coherent “single-crystal” superstructure solids is a major scientific challenge in nanoscience and nanotechnology. Besides advancing their optoelectronic functionalities, these “designer single-crystal” CQD solids are expected to become platforms to revolutionise technological advancement on many frontiers^1–5^. This includes the establishment of a quasi-two-dimensional giant superlattice formed by the monolayer assembly of the CQDs as the artificial giant atoms. Giant superlattices are particularly important, as seen in moiré superlattices of twisted bilayer graphene^6,7^, in which researchers found numerous emerging physical properties, including superconductivity, strongly correlated insulators, and topological states.

The significant challenge for forming a bottom-up solution-processable 2D giant superlattice from CQDs is to achieve highly conductive assembly of the QDs with bulk-like delocalised electronic wavefunctions together with a well-preservation of the quantum-confinement nature of the building blocks. Such assemblies are predicted to possess electronic miniband structures^8,9^, which could not be achieved in disordered assemblies or annealed and sintered assemblies^10–12^. Furthermore, the miniband formation can significantly enhance the carrier mobility and diffusion length. It is beneficial for designing QD solids with high electronic performance while retaining their unique properties stemming from the quantum confinement effect. Moreover, emerging electronic properties are also envisaged from these well-ordered CQD assemblies, e.g. Dirac cones and topological states, which have yet to be realised^13–15^.

Most of the current advancements in CQD technologies (displays, sensors, bio labels, solar cells) benefited from their unique optical properties, while charge carrier transport was still a detrimental factor^2^. Recent advances in the luminescence^16^, display^17,18^, lasing^19,20^, carrier multiplication^21–23^, upconversion^24,25^, magnetism^26,27^, and heat transport^28^ properties of the CQDs would require efficient charge carrier transport to realise the true all-QD direct electroluminescence devices, electrically-driven lasers, highly-sensitive detectors and scintillators, as well as thermoelectrics. The low conductivity in the utilised CQD solids stems from the spatially disordered assemblies and the localised nature of the charge carrier. Therefore, forming a well-defined CQD assembly with a minimum energetic and spatial disorder is crucial for understanding the essential and fundamental properties of electronic states^29^, which is also vital to advance device technology developments.

Here we report the electrical transport studies in PbS epitaxially-connected colloidal QD superlattice (QD-SL) and the observation of insulator-to-metal transition (IMT) by electric-field-induced doping, demonstrating the prospects of these material systems as a quantum material platform. IMT was only recently demonstrated in sintered photo-doped plasma-synthesised ZnO nanocrystals that promoted random connections of the particles in a disordered assembly^10,30^. To create a “confined-but-connected” system, the ordered nanocrystal assembly should be in large-area but with minimum energetic and spatial disorders, which should result in high electron mobility operation^31^. The unique characteristics of the colloidal-based QD assembly superstructures can be influenced by the original shape of their individual QDs. Long perceived as spherical shape objects, the realistic QDs consist of multiple facets stemming from their crystallographic structure. Rock-salt PbS QD has a truncated cuboctahedron structure with the facet’s size controlled by diameter^32,33^. The existence of known assembly superstructure phases, i.e. the body-centred-cubic (BCC) and face-centred-cubic (FCC) assembly structures and the intermediate phase body-centred-tetragonal (BCT), are among the consequential manifestations^29,34,35^. In this experiment, to form large-area 2D QD-SLs, we combine the gradual selective ligand stripping process and the facet attachment control during the self-assembly on the liquid surface by introducing chelating amine (i.e. ethylenediamine) as the chemical trigger to ensure the attachment of QD facets^36–38^. Electron microscopy, electron diffraction and small-angle/wide-angle X-ray scattering (SAXS/WAXS) confirmed the formation of large-area epitaxially-connected QD superlattice (QD-SL). Large area 1,2-ethanedithiol (EDT)-bridged QD assemblies were also prepared as controls.

Furthermore, ionic gating is utilised to electrostatically dope the QD solids continuously with high carrier density, sufficient to surpass the critical doping concentration for IMT in the epitaxially-connected QD-SLs. At room temperature, the electron mobility values of the QD-SLs are found to be independent of the diameters of the QD building block. The temperature-dependent resistance measurement transformed from the strongly localised hopping transport regime to the delocalised band transport behaviour at the epitaxially-connected QD-SL at high carrier density. The well-defined morphology in both the ligand-capped QD assemblies and the epitaxially-connected QD-SLs, as well as the continuous field-induced doping, allow us to observe the gradual transitions in charge carrier transport mechanism from Mott variable range hopping to metallic state surpassing the quantum conductance limit.

## Results

### Epitaxially-connected PbS QD assemblies

To realise the intrinsic properties of colloidal QD assemblies, the first challenge that should be overcome was the establishment of the large area quantum dots superlattice (QD-SLs) in which the QDs are strongly coupled and homogenous for the entire channel of the test devices. The superstructure of colloidal QDs assembly can be achieved by controlling the solvent evaporation that allows the low-rate self-assembly of QDs from bulk colloids into denser solid building blocks^35^. The complex assembly process occurs due to the ligand dynamics during the self-assembly process itself. Therefore, the initial conditions of both the bound ligands and the dissolved free ligands play crucial roles in determining the final structure of the assembly.

**Fig. 1:**
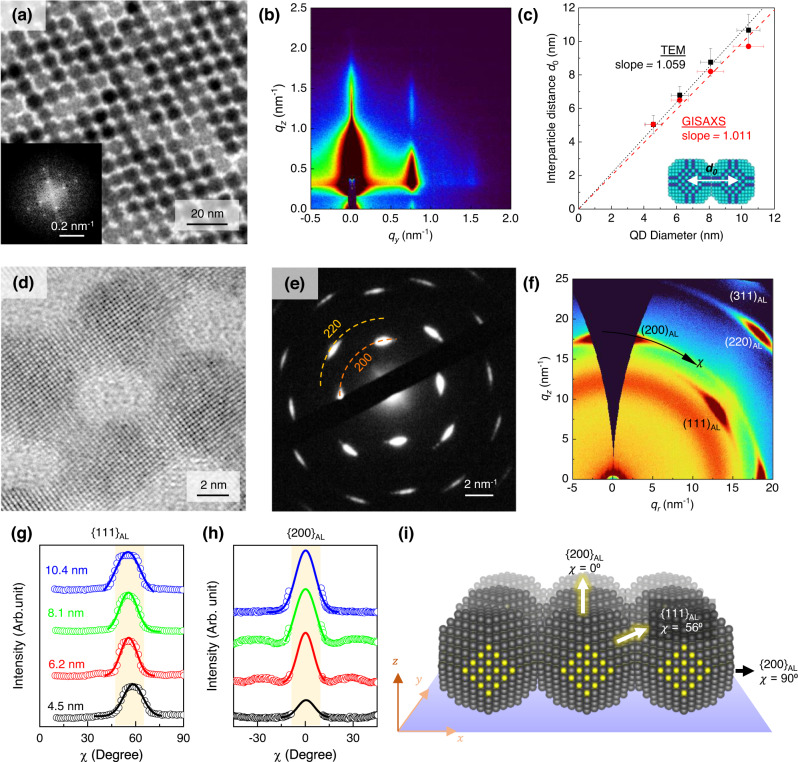
Epitaxially-connected PbS quantum dot superlattices (QD-SL). **a** Transmission electron micrograph (TEM) of the QD-SL containing 8.1 nm diameter PbS QD. (inset) Fast-Fourier transform (FFT) of the micrograph. **b** The grazing incident small-angle X-ray scattering (GISAXS) pattern of the corresponding sample indicates the two-dimensional ordering of the QD monolayer. **c** The plot compares the extracted average diameter of the QDs versus the centre-to-centre (inter-QD) distance from the TEM images (black squares), and GISAXS measurement (red dots) of QD-SLs fabricated from various diameters of QD building blocks. Error bars represent standard deviation. **d** High-resolution TEM shows the epitaxial connection of the QDs and **e** selected area electron diffraction (SAED), demonstrating the PbS atomic orientation in the QD-SLs. It is a conclusion supported by the **f** grazing incident wide-angle scattering (GIWAXS) pattern taken from a much larger sample cross-section. **g** Azimuthal line cut-off (*χ*) on scattered X-ray wavevector (***q***) for {111}_AL_ and **h** {200}_AL_ of the GIWAXS patterns show the consistency of the ordering for all QD diameters. **i** An illustration reconstructing the PbS QD-SL alignment on the substrate showing the atomic lattice orientation relative to the normal surface.

The epitaxially-connected PbS QD-SLs were fabricated using a modified liquid/air assembly (LAA) method that involves the self-assembly of QDs with partial surface ligand-removal and a subsequent process to induce complete attachment of QDs. However, the choice of subphase that induces partial ligand-removal and controls the solvent evaporation rate is crucial. It determines the re-arrangement of QDs at the liquid/air interface from the initial shape (hexagonal) to the denser superlattices. We used dimethyl sulfoxide (DMSO) as the subphase to strip the surface ligand moderately^39,40^. After the PbS QDs suspension (in hexane as the solvent) was completely dried at the subphase of DMSO, ethylenediamine (EDA) solution was injected into the liquid to promote the interfacial fusing among the neighbouring QDs^38,41,42^. Therefore, the entire LAA process resulted in the highly-ordered PbS QDs assembly (Fig. 1a). The LAA process is inclusive for QDs with different sizes ranging from 4.5 nm to 10 nm, with some adjustments on the initial concentration of QDs and ligand coverage demonstrated in this work (Supplementary Notes 1, Supplementary Figs. 1–3).

The morphology of the epitaxially-connected PbS QD-SLs was confirmed using both transmission electron microscopy (TEM) (Fig. 1a) and grazing incident small-angle X-ray scattering (GISAXS) measurement (Fig. 1b), highlighting the orders in both microscopic and macroscopic scales (Supplementary Notes 2). The obtained out-of-plane GISAXS pattern (*q*_*z*_) suggests the formation of a monolayer PbS QDs assembly (Supplementary Fig. 5) on a large scale^39,43,44^. At the same time, the atomic force microscopy (AFM) characterisation further confirmed the formation of monolayer assemblies (Supplementary Fig. 6). The pattern of the PbS QDs assembly exhibits a strong first Bragg peak on the in-plane (*q*_*y*_) axis, e.g. *q*_*y*_ ~ 0.74 nm^−1^ for 8.1 nm QD (Fig. 1b). The position of this first *q*_*y*_ Bragg peak indicates the centre-to-centre distance of the nearest neighbour QDs, information equivalent to the Fourier-transform of the TEM image. Figure 1c shows the relationship between size distribution and centre-to-centre distance of the assemblies built from different QD building block sizes, as confirmed using both TEM and GISAXS. Their linear fits with slopes close to unity suggest that the centre-to-centre distance is equal to the size of the constituent PbS QDs, implying direct connections between QDs without intervening ligand molecules.

The two-dimensional transformation of the PbS QD monolayer assembly from the hexagonal structure to the rhombus superlattice (Supplementary Fig. 7) follows the collective preferred orientation of the atomic lattices of each individual QD. High-resolution TEM (HRTEM) and grazing-incident wide-angle scattering (GIWAXS) measurements revealed that the characteristics of the atomic orientations in the assembly must satisfy two preferential conditions in both the out-of-plane direction (parallel to the surface of the substrate) and the in-plane direction. These two conditions determine the final structure of assemblies. The HRTEM of the epitaxially-connected PbS QD-SLs (Fig. 1d) shows the tendency of the uniformity of the specific atomic lattice orientation where the observed {100} atomic facets are facing up, revealing the other perpendicular {100} facet connection^33^. Figure 1e shows the selected area electron diffraction (SAED) pattern from the area of 250 nm in diameter. The sharp diffraction spots indicate the uniform preferential orientation of the atomic lattice over the scale of observation in the epitaxially connected QD-SL^39,45^.

Furthermore, the GIWAXS characterisation revealed the macroscopic atomic lattice orientation of the epitaxially-connected PbS QD-SL (Fig. 1f). The atomic lattice parameters of PbS QD were indexed by determining the 1D scattering vector (*q*) from azimuthal integration of the intensity (Supplementary Notes 3, Supplementary Fig. 8). The observed bright spots of the X-ray scattering indicate the preferential orientation of the atomic lattice in the epitaxially-connected PbS QDs assembly (Supplementary Fig. 9, for other sizes). The atomic lattice orientations were further quantitively analysed by performing line cut-offs along the azimuthal angle (*χ*) of the specific *q*-value (atomic lattice) from 0° to 90°. The preferential orientation of {111} atomic lattice and {200} atomic lattice of epitaxially-connected PbS QDs assembly with different diameters are plotted in Fig. 1g and h, respectively. The azimuthal line cut-off suggests that the preferential orientation of {111} atomic lattice is at *χ* ~ 56°, while the {200} atomic lattice is at *χ* = 0° (face-up), which satisfies the condition of the collective QDs arrangement illustrated in Fig.  1i^35,46^. Unlike those of epitaxially-connected QD-SL, the SAED and GIWAXS patterns of the OA-capped (Supplementary Fig. 10) and EDT-bridged QDs assemblies (Supplementary Fig. 11) showed ring powder-like patterns, suggesting that the atomic lattice on those assemblies are un-oriented, particularly to the out-of-plane direction of the assembly.

**Fig. 2:**
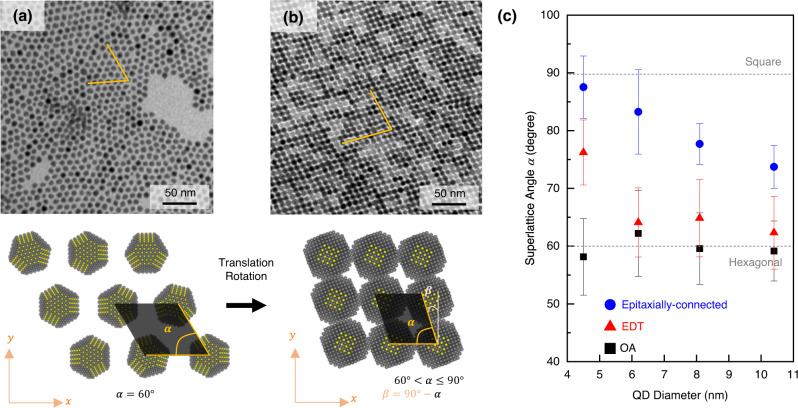
In-plane arrangement of the PbS QD-SLs. **a** TEM image of oleic-acid (OA)-capped PbS QDs showing the 2-dimensional hexagonal structure, and **b** the TEM image of the epitaxially-connected PbS QD-SL. Upon assembly, selective ligand stripping and re-orientation, the QD-SLs transform from hexagonal lattices to quasi-square lattices or rhombic lattices with superlattice angle *α*. **c** Plot comparing the superlattice angle *α* as the function of QD diameters when prepared with different interparticle spacing: epitaxially connected by DMSO + EDA treatment (blue dots); EDT-bridged by ligand exchange (red triangles); or OA-capped (black square). Error bars represent the standard deviation.

In addition to the out-of-plane direction, we found another collective behaviour of the assembly in the in-plane direction. The QD diameter and distance among the neighbouring QDs influence the orientation of the assembly. This collective behaviour is described by the superlattice angle (*α*) parameter. The OA-capped PbS QDs assembly forms the typical hexagonal structure with *α* = 60° (Fig. 2a). All QD diameters always form this hexagonal structure. In contrast, in epitaxially-connected PbS QD-SLs, the *α* value increases close to 90° on the smallest QD (square lattice). As the diameter of the QD building block increases, the superlattices form epitaxially-connected rhombic SLs with *α* value gradually decrease (75° < *α* < 90°) (Fig. 2b). In another case where the interparticle distance is reduced by ligand exchange, i.e. EDT-bridged PbS QDs assembly, only the small QDs (4.5 nm) exhibited the structural re-configuration to form a rhombic lattice with 70° < *α* < 80° (Supplementary Fig. 12). Meanwhile, for larger QDs, the *α* was only increasing slightly but still showed a hexagonal structure. Figure 2c shows the summary of the change in *α* upon the increase of QD size with different inter-QD spaces.

The possible origin of the epitaxially-connected QD-SL formation with α below 90° is the anisotropic rotation of each QD in the in-plane and out-of-plane direction when they are floating on the subphase. The high rate of ligand detachment and the quick connection process do not provide sufficient time for each QD to fully rotate and adjust itself to form a perfect square superlattice (*α* = 90°). Upon transition from FCC to BCC in three-dimensional QD assemblies, the rotation of the individual QDs is known to drive the assembly structure, as observed by the orientation of specific atomic lattice facets^35,47^.

In the demonstrated quasi-2D monolayer of epitaxially-connected QD-SL, the superlattice angle is congruent to the orientation of the atomic lattice of each QD in the in-plane direction. Consequently, the epitaxial connection along {100} facet of the QDs forms an atomic lattice offset^47^. Therefore, not all surface atoms on the {100} facet of the QD will be epitaxially connected to the {100} facet of the neighbouring QD. This offset becomes more significant as the QDs size increases, affecting the true size of the cross-section area of the epitaxial connection among neighbouring QDs. Although larger QD would have a larger area of {100} facet, the smaller *α* value reduces the effective epitaxial cross-section area. It implies that controlling the coupling between QDs by size tuning is not trivial and great care in facet engineering of the truncated cuboctahedral of PbS QD is vital. Such intricate atomic lattice orientation control can only be performed in epitaxially-connected QD-SLs. Although they may form well-ordered assemblies, atomic lattice orientation cannot be achieved in ligand-capped QD assemblies.

### Charge carrier transport measurement

**Fig. 3:**
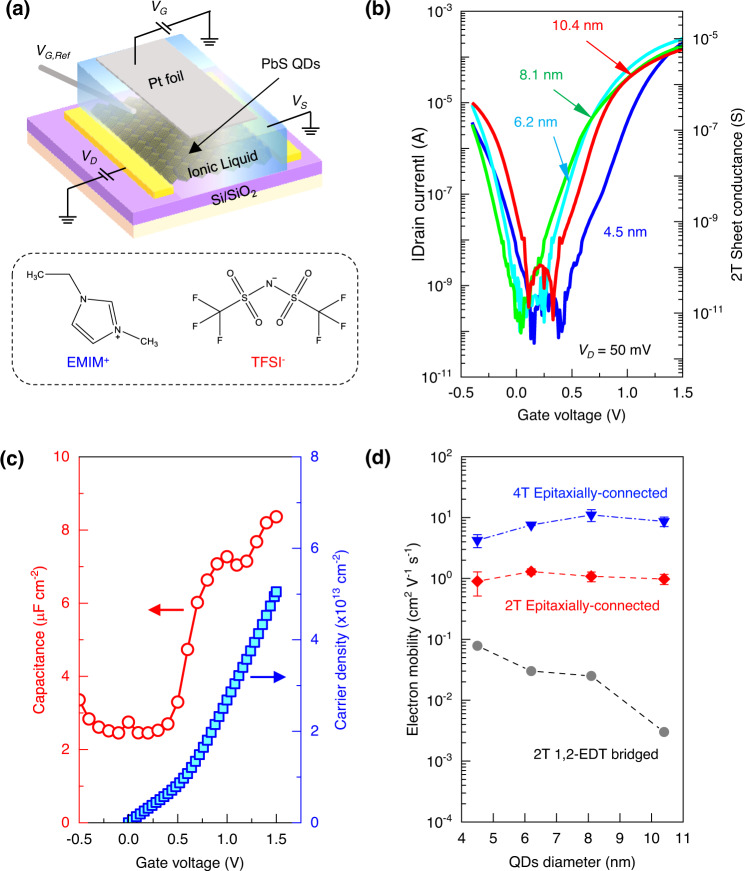
Size-independent mobility in electric-double-layer transistors (EDLT) of PbS epitaxially-connected QD-SLs. **a** A schematic of the two-terminal (2T) EDLT device structure of the PbS QD-SL using EMIM-TFSI ionic liquid as electrolyte gate. **b** *I*_*D*_–*V*_*G*_ transfer characteristics of the QD-SLs prepared from PbS QD with different diameters show ambipolar transport with a well-defined intrinsic region and a high on/off ratio for holes and electrons. **c** The gate-voltage-dependent capacitance value of the EDLT (red circle) and the corresponding accumulated sheet electron density (blue square) were extracted from potentiostatic electrochemical-impedance-spectroscopy (EIS) measurements. **d** Plots of the characteristic electron mobility values of the epitaxially-connected QD-SLs EDLT versus the diameter of the QDs (red diamond) showing weakly size-dependency, in contrast to the conventional (decreasing) size-dependent electron mobility in the ligand-capped QD assemblies (i.e. EDT-bridged PbS QD, grey circle). The four-terminal (4T)-EDLT mobility values of the epitaxially-connected PbS QD-SLs (blue triangle) show higher values than their two-terminal (2T)-EDLT mobility values, with a similar size-independent trend. Error bars represent the standard deviation.

To characterise the electronic properties of epitaxially-connected PbS QD-SLs, we employed the electric-double-layer transistor (EDLT)^48^. Figure 3a shows the typical two-terminal EDLT device where the epitaxially-connected PbS QD-SLs were transferred onto Si/SiO_2_ substrate with a pre-patterned interdigitated-Au electrode (*L* = 10 µm or 20 µm; *W* = 1 cm). The QD-SLs were then covered by ionic liquid EMIM-TFSI [1-Ethyl-3-methylimidazolium bis (trifluoro-methylsulfonyl)-imide], which is capable of accumulating very high carrier density (~10^14^ cm^−2^) and filling the carrier traps^49–51^. Figure 3b shows the *I*_*D*_*–V*_*G*_ transfer characteristics of epitaxially-connected PbS QD-SLs EDLTs formed using various QDs diameters. All the transfer curves were taken from the linear operation (*V*_*D*_ = 50 mV) of the devices (Supplementary Fig. 14). All devices demonstrated ambipolar transport characteristics with high on/off ratio values, reaching ~10^5^ for their electron currents (*V*_*G*_ > 0 V). Well-defined off-current regions between the hole-enhancement and electron-enhancement arms were observed, suggesting the complete filling of the in-gap trap states by the EDLT measurement (Supplementary Fig. 15). These observations also suggest that the epitaxially-connected QD-SLs retain their semiconducting properties.

The transfer characteristics of the EDLTs, with the well-defined insulating region, also allowed us to perform electronic spectroscopy of the QD-SLs^52,53^, confirming the electronic bandgap of the assemblies. Although the QDs are epitaxially connected, the observed size-dependent bandgap values probed by the EDLTs (Supplementary Fig. 20) and the excitonic peak in their absorption spectra confirmed that the quantum confinement nature is preserved (Supplementary Fig. 21).

Having EDLTs built from epitaxially-connected QD-SLs with different diameters allowed us to relate the charge carrier mobility and the size of the building blocks. The charge carrier mobility values of the EDLTs were extracted by first determining the accumulated carrier density. The charge carrier density was determined from the capacitance obtained from electrochemical impedance spectroscopy (EIS) measurement (Supplementary Note 4, Supplementary Fig. 22). EDL gating has a very strong voltage-dependent capacitance, distinctive from solid gate dielectric. The gate-dependent capacitance values of the EDLTs were measured using EIS on each identical device. The *C–V*_*G*_ curve (Fig. 3c) exhibits a plateau of finite capacitance value below the electron accumulation threshold, then increases. It implies that the accumulated carrier initially filled the trap sites before contributing to conduction in the band, suggesting the observed electronic conductance is intrinsic.

The EDLTs of the epitaxially-connected QD-SL demonstrate electron mobility trends distinctive from the conventional ligand-bridged QD assemblies (Fig. 3d, Supplementary Fig. 23). The observation of higher electron mobility in the epitaxially-connected QD-SL than in the state-of-the-art EDT-bridged QD assembly by more than one order of magnitudes is consistent with the expectation^54^. More interestingly, the epitaxially-connected QD-SLs also demonstrate nearly constant electron mobility values against the QD diameter, which displays marked contrast with the current understanding^55,56^. It has been established that the carrier mobility in QD assemblies should have strong size dependency, i.e. the electron mobility decreases by increasing QD diameters, as demonstrated in the EDT-bridged QD assemblies.

We then performed temperature-dependent conductivity measurements of the EDLTs to investigate the localisation and delocalisation of electrons in the epitaxially-connected QD-SLs. The EDLTs allow us to modulate the conductance by significantly changing the chemical potential of the charge carriers. The temperature dependence of the sheet conductance was measured after the EDL induced the electron accumulation at room temperature. When the ionic liquid is frozen, the accumulated electron density persists in the transistor channel^57,58^. The temperature-dependent measurement was performed down to 30 K in a close-loop He cryostat connected to a nitrogen-filled glovebox. Similar to the room-temperature transport measurement, the devices have never been exposed to ambient air or moisture.

**Fig. 4:**
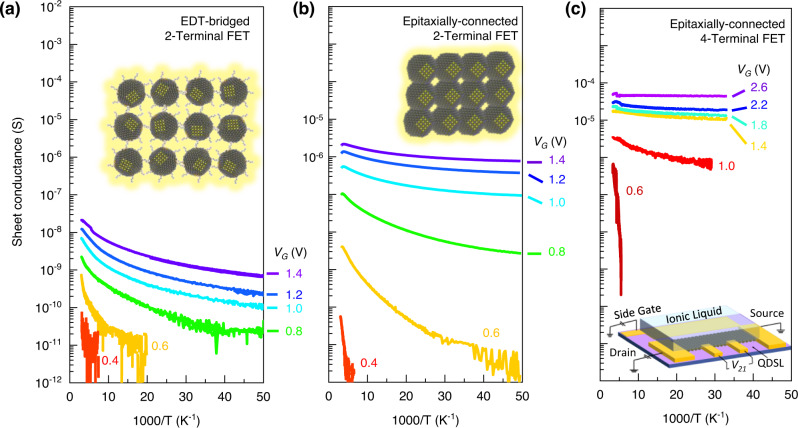
Electron delocalisation in the epitaxially-connected PbS QD-SLs. **a** Temperature-dependent Arrhenius plot of the two-terminal (2T) electron sheet conductance (*G*_*sheet*_) in EDT-bridged PbS QDs assembly under application of different charge carrier density values accumulated by field-induced doping of EDLT. **b** A similar plot of the electron transport in the epitaxially-connected PbS QD-SL shows higher electron conductivity and stronger deviation from Arrhenius-type hopping transport behaviour. **c** The corresponding four-terminal (4T) *G*_*sheet*_ of the EDLT demonstrates even higher intrinsic electron conductance, free from the influence of the significant contact resistance. Upon application of much higher carrier density (*V*_*G*_ > 1.4 V), the electron transport process transformed on the verge of delocalisation (d*G*_*sheet*_/d*T* < 0). (inset) Illustration of the 4T-EDLT device.

Figure 4a, b directly compares the temperature-dependent plot of the electron sheet conductance of the EDT-bridged PbS QD assemblies and the epitaxially-connected PbS QD-SL. They were obtained from two-terminal EDLTs under different applied gate voltage values. Consistent with the previous findings in the room-temperature measurements at atmospheric pressure, the epitaxially-connected QD-SL conductivity is generally two orders of magnitude higher than the EDT-bridged QD assembly. The EDT-bridged QD assembly shows hopping-type behaviour where the conductivity values decrease by temperature. Similarly, epitaxially-connected QD-SL also show a trend of decreasing conductivity by temperature (Fig. 4b), especially at low gate voltage applications. However, the temperature-dependent conductivity trends for the EDT-bridged QD assembly and the epitaxially-connected QD-SL strongly deviate from Arrhenius-type hopping transport behaviour, where the logarithm of the conductivity should be linearly decreasing by the inverse of temperature.

To further elucidate the intrinsic charge carrier transport mechanism of the epitaxially-connected QD-SLs, four-terminal (4T) EDLT measurement is a compulsion. The homogeneity of the QD-SL over a large area makes the fabrication of reliable 4T-EDLTs with adequately large channels possible. The four-terminal sheet conductance, *G*_*sheet*_ = (*L*_*4T*_/*W*)(*I*_*DS*_/*V*_*4T*_), can be measured as a function of the applied gate voltage. The *L*_*4T*_ and *V*_*4T*_ are the distance between the four-terminal voltage probes (20 µm) and the potential difference between these probes, respectively. *G*_*sheet*_ value reaches a value of more than 14 µS at *V*_*G*_ = 1.0 V at *T* = 300 K for the devices prepared using 8.1 nm PbS epitaxially-connected QD-SL (Supplementary Fig. 25b). The highest room-temperature intrinsic electron mobility value deduced from this four-terminal measurement was found to be 13.5 cm^2^ V^−1^ s^−1^ (at *V*_*G*_ = 1.5 V), which is more than one order of magnitude higher than the value obtained from its corresponding two-terminal devices. It suggests that the interface of Au and PbS QDs still generates a high contact resistance value (Supplementary Fig. 27), hampering firm measurement of the intrinsic properties of the QDs. The achieved value of the intrinsic electron mobility, which is among the record high for semiconductor QD assemblies, stems from the large-area ordering of the epitaxially-connected QD-SLs. The extracted mobility trend from the 4T-EDLT measurements agrees well with the size-independent transport characteristics observed in two-terminal (2T) EDLTs of the epitaxially-connected QD-SLs (Fig. 3d).

Figure 4c shows the temperature-dependent of four-terminal sheet conductance of the epitaxially-connected QD-SL. Generally, the conductivity values at high temperature in this device is more than one order of magnitude higher than the values obtained from the two-terminal counterpart. Resembling the two-terminal device, for *V*_*G*_ = 0.6 V, typical semiconducting behaviour is observed following the hopping transport regime. Increasing the applied gate voltage values further deviates the temperature dependence of the conductance away from the Arrhenius behaviour. Starting from *V*_*G*_ = 1.4 V, the low-temperature part of the conductivity trend starts showing nearly constant sheet conductance.

At *V*_*G*_ = 2.6 V, the electron conductance is nearly temperature-independent for the whole measurement, with *dG*_*sheet*_/*dT* slightly having a positive value in the high-temperature region. *G*_*sheet*_ (300 K) and *G*_*sheet*_ (30 K) are 55 µS and 50 µS, respectively, above the quantum conductance (*G*_*Q*_) value, *e*^2^/*h* = 39 µS. Achieving *G*_*sheet*_ above *G*_*Q*_ value is the essential parameter to acquire such an insulator-to-metal transition (IMT)^59^, which has never been observed in semiconducting colloidal QD materials assemblies.

## Discussion

The most striking difference in the electron transport properties between the EDT-bridged PbS QD assemblies and the epitaxially-connected QD-SLs is the size dependency of the electron mobility values (Fig. 3d). In the EDT-bridged PbS assembly, the mobility decreases with increasing their size. The current understanding of such size-dependent mobility is the phonon-assisted hopping transport model^56^, where the electronic hopping between the QDs is assisted by phonons. However, this hopping should get weaker as the diameter of the QDs increases due to the smaller wavefunction overlap^55^. This is because the spillover of the wavefunction is smaller when the QD diameter increases. In sharp contrast, the electron mobility values in the epitaxially-connected QD-SLs show weak dependency against the QD diameter. The differences in the mobility values are within a similar order of magnitude. This observation suggests that the hopping transport mechanism no longer governs their charge carrier transport process.

The controllability of the assembly formation and the fine-tuning of the carrier density by the ionic gating enable us to discuss the charge carrier transport in the QD assemblies. The high carrier density accumulation in the compact and well-ordered monolayer of EDT-bridged QD assemblies shows that the transport mechanism strongly deviates from the nearest-neighbour hopping (NNH) Arrhenius-type behaviour, which is transforming to the variable range hopping (VRH). We then compared possible scenarios of different types of VRH. Within the given temperature range, the transport process fits best with Mott 3D-VRH (Supplementary Fig. 24c). However, we found that the fit also works well with Efros-Skhlovskii (ES) and Mott 2D-VRH and (Supplementary Figs. 24a and 24b, respectively). Rigorous identification of the detailed mechanisms should be made at lower temperatures, e.g., *T* < 20 K^60^, which was not covered in the current study and thus is left for a future study. In the case of epitaxially-connected QD-SLs, similar observation is only found at low carrier density at *V*_*G*_ below 1 V (Supplementary Fig. 24g). The steeper temperature dependence of the VRH-like behaviour in the EDT-bridged PbS QDs than the epitaxially-connected QD-SLs indicate that more significant disorder remains due to the non-coherence of the QD orientation, despite the uniform inter-QD distance.

The intrinsic electron transport in the epitaxially-connected QD-SLs transport, as probed by 4-terminal measurements, exhibit distinct behaviour. Upon application of a higher gate voltage of 1.4 V ≤ *V*_*G*_ ≤ 2.2 V, the conductivity becomes almost temperature independent. This behaviour is well fitted by the Mott-VRH (Supplementary Fig. 28a) as well as by the 2D weak localisation, or Anderson localisation mechanism (Supplementary Fig. 28b). The observation of possible weak localisation and *T*-independent conductivity suggests that the electron transport in the epitaxially-connected QD-SLs is not predominantly governed by the hopping between the individual QD anymore but is determined by the band-like transport with remaining weak disorder. It may not contradict the size-independent mobility in epitaxially-connected QD-SLs.

The observation of temperature-independent conductance towards a finite zero-degree conductance with values higher than the quantum conductance *e*^2^/*h* at very high carrier density suggests an early indication of delocalised (metallic) behaviour. A conductance value below *e*^2^/*h* indicates that the electron mean-free-path is shorter than the Fermi wavelength, so quantum interference becomes a dominant feature in electron diffusion. In recent years, some theoretical framework has been pioneered to anticipate the possibility of realising insulator-to-metal transition in QD-SLs^12,61^. For semiconductor QD-SL that are epitaxially connected at touching radius *ρ*, the insulator-to-metal transition can occur at a critical carrier density value of ${n}_{c}{\rho }^{3}\, \approx \, 0.3g$, where *g* is the number of degeneracies of conduction band minima^12^. In the case of lead-chalcogenide, including PbS, the number of state degeneracies is 4^59,62,63^. In our epitaxially-connected QD-SL, constructed from 8.1 nm PbS QD, the epitaxy touching radius *ρ* was measured at around 2.3 nm (Supplementary Fig. 29). Consequently, the estimated critical carrier density value, *n*_*c*_, to achieve the transition is around 2.1 × 10^13^ cm^−2^. Considering the structure of the epitaxially-connected assembly with the given QD size, we estimated the QDs density as about 1.5 × 10^12^ dot cm^−2^. Hence, this number of dots gives us the charge carrier density value of ~14 electrons/dot, sufficient to fill the lowest quantised energy state in the conduction band on PbS QDs. This value was reached in the present experiment by applying *V*_*G*_ of more than 1.0 V.

Surpassing the theoretical value of the critical carrier density for the insulator-to-metal transition can explain the observation of the nearly temperature-independent conductivity when we applied a larger gate voltage. Notably, the carrier density obtained here is in the same order as the previous work on doped plasma-synthesised ZnO nanocrystals, where the insulator-to-metal transition was also observed^10^. As the gate voltage was applied above 1.5 V, we obtained carrier density accumulation for more than 30 electrons/dot. It indicates that transport on the higher quantised energy level was achieved. It also suggests that the electron transport in the QD-SL starts to become more delocalised on this energy level.

Achieving insulator-to-metal transition and knowing the corresponding numbers of accumulated carrier in the epitaxially-connected QDs, we were able to estimate the carrier mobility values from the quantum conductance and the carrier density^12^. This estimated electron mobility is in a good agreement with the value obtained from 4T-EDLT measurements (see Supplementary Note 5C).

The above observation unambiguously proved that the epitaxial connection of neighbouring QDs and their orientational order are essential to realising the metallic state of QD assemblies. The importance of the orientational order can also explain the origin of the size-independent mobility values in epitaxially-connected QD SL system. Since the insulator-to-metallic transition goes from Mott VRH through either Mott or Anderson transitions, minimising residual disorders is vital to realising true metallic states. To ultimately realise and exploit true metallic states in this PbS epitaxially-connected QD-SL, further reduction of the surface-related electron traps, chemical doping, and further enhancement for field-induced doping by utilising more capacitive electrolytes are highly anticipated.

In conclusion, our work demonstrated that epitaxially-connected QD-SLs of semiconducting compound (PbS) could achieve insulator-to-metal transition by tuning the carrier density via field-induced doping utilising ionic liquid gating. Optimised attachment process through the step-by-step selective ligand stripping process ensures the formation of large-area well-oriented epitaxial assemblies. The control of the size of the attachment cross-section, superlattice angle, and the electronic coupling of the QD-SLs allowed the minimisation of disorders in the “attached but confined” system, promoting charge carrier delocalisation. Demonstrating carrier delocalisation in the QD-SLs would be advantageous for further exploring the QD assemblies as designer materials with the potential observation of emergent phenomena and advancing practical device developments. Furthermore, it may also pave the better way to realise new kinds of 2D giant superlattice, formed by the QD as the giant atoms through the solution process, complementary to the established twisted stack 2D material system.

## Methods

### Materials

After synthesis protocol customisation, high-quality lead sulphide (PbS) QDs with various diameters were tested and procured from Quantum Solutions (www.quantum-solutions.com). Dimethyl sulfoxide (DMSO, anhydrous, 99%, Sigma-Aldrich), methanol (anhydrous, 99%, Sigma-Aldrich), acetonitrile (anhydrous, 99%, Sigma-Aldrich), 1,2-ethanedithiol (EDT, Sigma-Aldrich), ethylenediamine (EDA, Nacalai Tesque), 1-Ethyl-3-methylimidazolium bis(trifluoro-methylsulfonyl)imide (EMIM-TFSI, Kanto Chemical Co., Inc.) were procured as solvents, subphases, and ionic liquid gates, which were always stored inside N_2_ gloveboxes.

### Preparation of epitaxially-connected PbS QDs superstructure

The superlattice assembly of PbS QDs was prepared using the liquid/air interfacial method (LAA)^39,50^. DMSO was used as the subphase in these experiments instead of acetonitrile. 2 ml DMSO was poured into a (2 × 2 × 1.5) cm^3^ PTFE bath. Then, 20 µl of PbS QD solution (1–2 mg mL^−1^, in anhydrous hexane) was dropped onto the DMSO subphase. The PTFE bath was then covered by slide glass immediately while the QD solution spread, suppressing the hexane evaporation rate. This condition was maintained for 30–60 min to allow the partial ligand stripping of oleic acid from the surface of QDs by DMSO. Subsequently, 20 µl of EDA solution (1 M, in DMSO) was gently injected into the subphase. After keeping the condition for 5 min, the PbS QDs assembly was collected by solid substrate or TEM grid.

### TEM, HRTEM, HAADF-STEM, and SAED characterisation

Transmission electron microscope (TEM) images were collected using a JEM-1230 apparatus (JEOL) operated at 80 kV. Selected area electron diffraction (SAED) and high-resolution TEM (HR-TEM) images were taken using JEM-2100F/SP (JEOL) operated at 200 kV. High-angle annular dark-field scanning TEM (HAADF-STEM) images were captured using Talos F200X (Thermo Fischer). The SAED images were taken on 0.06 µm^2^ of observation area with magnification at 100,000× and 400,000× using a camera length of 80 cm of JEM-2100F/SP (JEOL). The inter-QD distance and the QD diameters were analysed using ImageJ (National Institutes of Health, USA) (>100 data points each and magnification of 400,000×). Before TEM measurement, the prepared QD assembly on the TEM grid was baked at 100 °C for 1 h in the nitrogen glovebox.

### GISAXS/GIWAXS measurements

The GISAXS and GIWAXS measurements were conducted at the BL38B1 beamline of the RIKEN SPring-8 synchrotron radiation facility (Hyogo, Japan) using 12.4 keV monochromatic X-ray (*λ* = 1.0 Å). The X-ray beam was produced by a bending magnet at the 8 GeV storage ring. It is monochromatised using an unsymmetrical Si(111) double crystal monochromator, and an Rh courted bend cylindrical focusing mirror. An array of PILATUS 3X 2 M detectors (Dectris) with 1475 × 1679 pixels (pixel size of 172 × 172 µm^2^) was used to record the 2D scattering patterns. It was adjusted to a sample-to-detector distance of 2.548 m and 0.285 m for GISAXS and GIWAXS experiments, respectively, as calibrated by the silver behenate standard. The X-ray beam was collimated to a spot size at the sample of 150 µm × 150 µm. It gave a typical beam size of 700 µm × 150 µm at the sample position with a camera length of 2.548 m). The grazing incident angle of the X-ray was tuned from 0.10° to 0.30°. The QD-SL assemblies were prepared on bare SiO_2_/Si substrate, identical to those used for device fabrication. Identical samples were used for both GISAXS and GIWAXS measurements. All GISAXS and GIWAXS data were collected upon 10 s of exposure per image. The collected data were analysed using FIT2D (European Synchrotron Radiation Facility, ESRF)^64^ and MATLAB®-based GIXSGUI software suite^65^.

### Device fabrication and measurement

All field-effect transistors were fabricated on SiO_2_/Si substrates with lithographically patterned electrodes. Maskless UV lithography (D-light DL1000RS), which has a writing resolution of 1 µm, was utilised to write the lithography pattern on AZ1500 photoresist deposited on 300 nm thermally grown SiO_2_ on Si wafer, with hexamethyldisilazane (HMDS) was used as the priming layer. As electrodes, 30 nm Au was thermally evaporated with a 10 nm evaporated Ti adhesion layer. Afterwards, a standard lift-off process was performed using acetone. Before the QD-SL transfer, the pre-patterned substrates were further cleaned, including plasma cleaning. The two-terminal devices, consisting of interdigitated electrode patterns, have a total channel width of 1 cm and a channel length of 10 µm or 20 µm. The four-terminal (4T) devices have a channel width of 400 µm, a channel length of 25 µm or 50 µm, and a 10 µm or 20 µm distance between the voltage probes. In the case of the 4T device, designed for side-gate EDLT, a 100 nm SiO_2_ passivation layer is deposited to protect the vias on the substrate.

After the QD-SL transfer process, the samples were baked at 100 °C for 1 h. Subsequently, the ionic liquid (EMIM-TFSI) was dropped onto the PbS QD-SL channel. For two-terminal devices, Pt foil was then placed as the gate electrode.

Room temperature transport was measured in the dark using a low-noise probe station inside the N_2_-filled glovebox and connected to a Semiconductor Device Analyzer (Keysight B1500A). The transfer characteristic measurements were performed under a *V*_*G*_ sweep rate of 50 mV s^−1^. The range of applied *V*_*G*_ values was −1 V to 1.5 V, within the electrochemical windows of the EMIM-TFSI ionic liquid. The low-temperature transport was measured using a close-loop 4 K vacuum GM cryocooler (Ulvac Cryogenics), which can cool down its sample stage to 10 K. This cryostat is connected to a dry N_2_-filled glovebox (ALS Technology), specially designed for device measurement. Programs involving a temperature controller (Lakeshore LS335), a Semiconductor Parameter Analyser (B1500A, Keysight) and a nanovoltmeter (34420a, Keysight) instruments were used to perform the transport measurement.

The capacitance of the EDLTs (PbS QDs/IL/Pt foil structure) was measured using electrochemical impedance spectroscopy (EIS) in two-electrode potentiostat mode (VersaSTAT 4, AMETEK Scientific Instruments). The measurement was performed using AC potential of *V*_*RMS*_ = 10 mV with frequency scanned from 100 kHz to 0.5 Hz. The device was subjected to variations of applied *V*_*G*_. The applied *V*_*G*_ values were within the respective electrochemical window of the ionic liquids. The interval between the applied *V*_*G*_ was 0.1 V. Gate-voltage-dependent impedance spectra were then obtained, from which the capacitance and the accumulated carrier density can be deduced^48,57^.

## Supplementary information

Supplementary Information

Peer Review File

## Data Availability

Source data are provided with this paper.

## Source data

Source Data
