# Supplementary material for: Enabling metallic behaviour in two-dimensional superlattice of semiconductor colloidal quantum dots
Source: Nat Commun. 2023 May 26;14:2670. doi: 10.1038/s41467-023-38216-y (PMC10220219; doi:10.1038/s41467-023-38216-y)
Supplement: Supplementary file 1 — Supplementary Information [file 41467_2023_38216_MOESM1_ESM.pdf]

Supplementary Information:

Enabling Metallic Behaviour in Two-Dimensional Superlattice of Semiconductor Colloidal Quantum Dots

Ricky Dwi Septianto,[1,2] *Retno Miranti,*[1] *Tomoka Kikitsu,*[1] *Takaaki Hikima,*[3] *Daisuke Hashizume,*[1] *Nobuhiro Matsushita,*[2] *Yoshihiro Iwasa,*[1,4] and *Satria Zulkarnaen Bisri,*[1,2,5]*

[1]RIKEN Center for Emergent Matter Science (CEMS), 2-1 Hirosawa, Wako, Saitama 351-0198, Japan

[2]Department of Materials Science and Engineering, Tokyo Institute of Technology, 2-12-1 Ookayama, Meguro, Tokyo 152-8550, Japan

[3]RIKEN SPring-8 Center, 1-1-1 Kouto, Sayo, Hyogo, 679-5198, Japan

[4]Quantum Phase Electronic Center (QPEC) and Department of Applied Physics, The University of Tokyo, 7-3-1 Hongo, Bunkyo-ku, Tokyo 113-8656, Japan

[5]Department of Applied Physics and Chemical Engineering, Tokyo University of Agriculture and Technology, 2-24-16 Nakacho, Koganei, Tokyo 184-8588, Japan

* Correspondence to: satria-bisri@go.tuat.ac.jp

Supplementary Note 1: PbS colloidal quantum dots

A. Lead Sulphide (PbS) Colloidal QDs

In this work, we used PbS QDs with different diameters labelled as 4.5 nm, 6.2 nm, 8.1 nm, and 10.4 nm, where the size is determined by measuring the diameter from TEM images, as shown in Supplementary Fig. 1. The size distribution was collected from more than 100 particles in each sample. The optical properties of the corresponding size were evaluated by UV-VIS-NIR absorption spectroscopy, and the absorbance spectrum was recorded in Supplementary Fig. 2. The PbS QDs with diameters of 4.5 nm, 6.2 nm, 8.1 nm, and 10.4 nm exhibit well-defined excitonic peaks and valleys with high peak-to-valley ratios. The associated excitonic peaks are located at 1295 nm, 1584 nm, 1865 nm, and 2050 nm, respectively.

B. Elemental Analysis

Rutherford Backscattering (RBS) measurement was employed to measure the ratio of Pb atom and S in PbS QDs with different sizes (Supplementary Fig. 3). The measurement was conducted at RIKEN Nishina Center for Accelerator-Based Science (RNC) using a Pelletron 5SDH2 (National Electrostatics Corp., Middleton, USA) with a $^{4}He^{++}$-ion beam source (2.2 MeV) with a 5 mm beam size. The detector was placed at 165° of backscattering angle. The RBS samples were prepared using OA-capped PbS QDs (original state) via the LAA method on acetonitrile without any ligand exchange or removal.

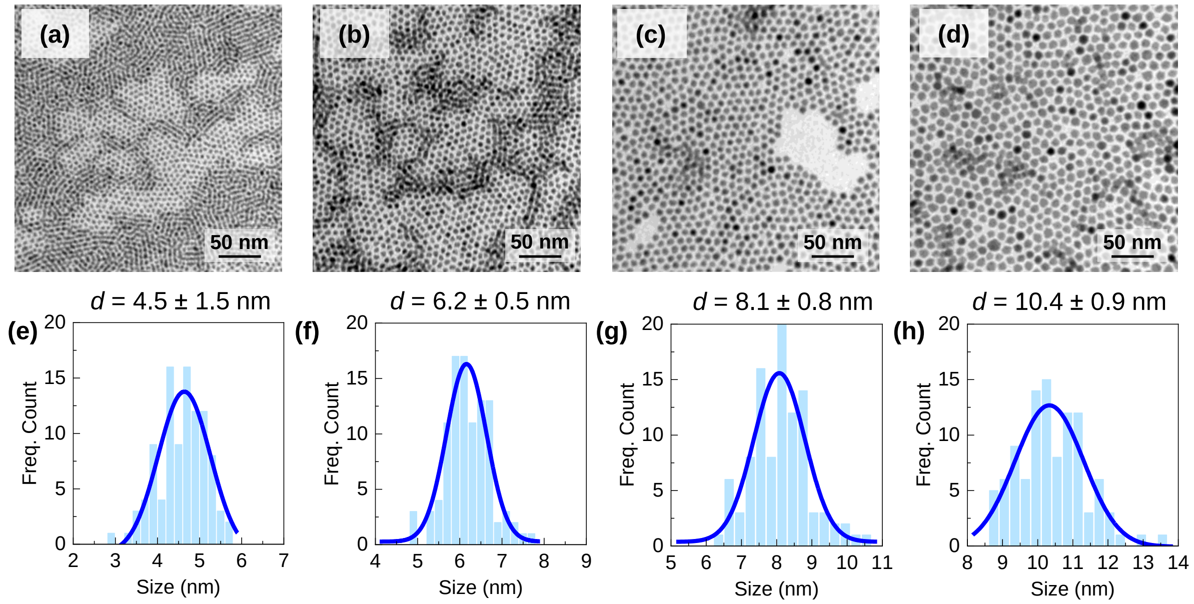

Supplementary Figure 1 | TEM images of PbS QDs with different size (a) 4.5 nm, (b) 6.2 nm, (c) 8.1 nm, and (d) 10.4 nm. (e)-(h) are the corresponding histogram size distribution of each size, respectively.

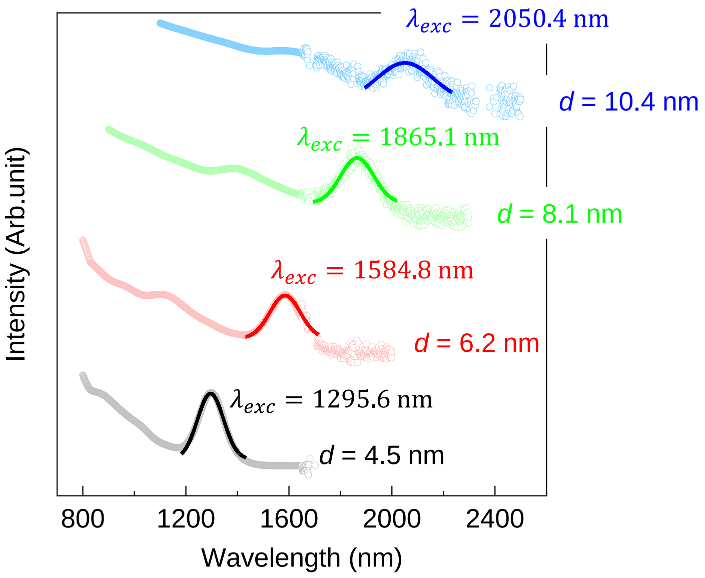

Supplementary Figure 2 | The absorption spectra of PbS QDs with different diameters show well-defined excitonic peaks and valleys.

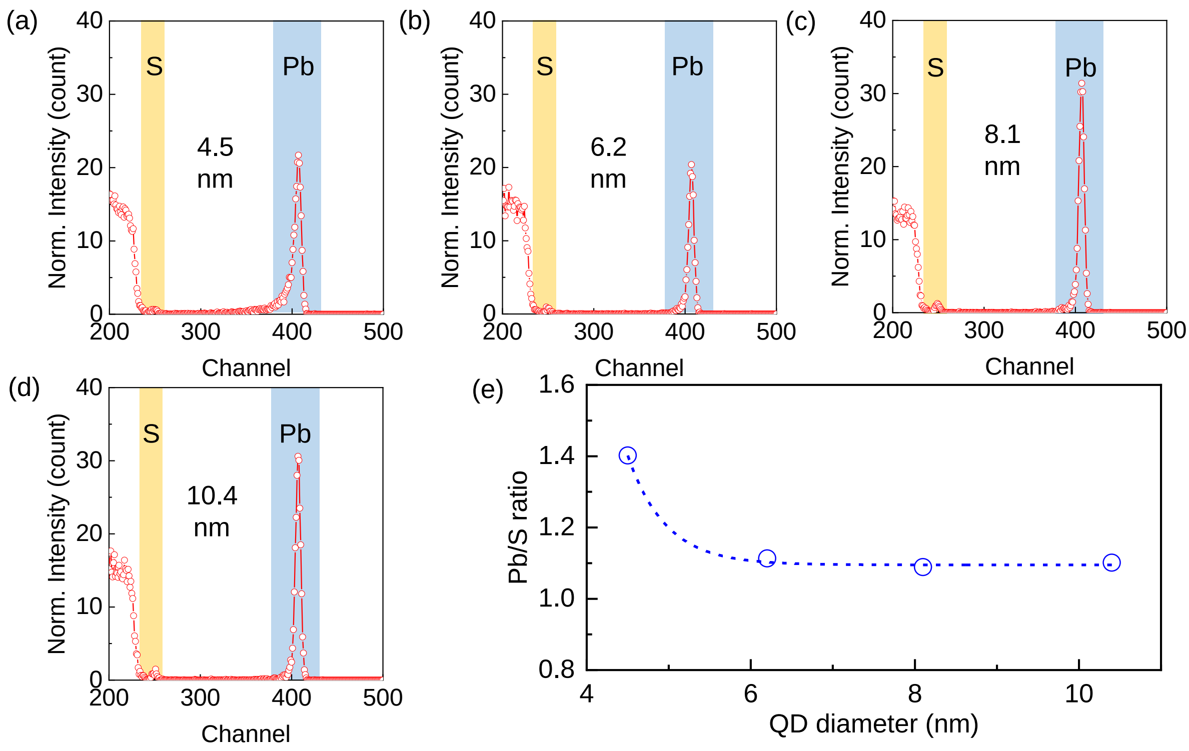

Supplementary Figure 3 | Elemental analysis of PbS QDs by Rutherford Backscattering (RBS) measurement. The RBS profile of PbS QDs with diameters of (a) 4.5 nm, (b) 6.2 nm, (c) 8.1 nm, and (d) 10.4 nm. (e) The deduced Pb to S ratio over the PbS QDs with different size variations.

Supplementary Note 2: Determination of the centre-to-centre distance among QDs in the assemblies and superlattices

The centre-to-centre distances among QDs were evaluated from TEM images and GISAXS pattern analysis. From the TEM image analysis, we determined the distance of the nearest neighbour QDs, which depends on the geometry of the assembly. For epitaxially-connected QDs with rhombic shape, demonstrated by all PbS QDs with different diameters (Supplementary Figs. 4a-4d**)**, the nearest neighbour distance among QDs was defined by the direction of the connected facet. In this case, one QD is connected to the other four QDs through four sides (facet) connection, as illustrated by the circle and the yellow line in Supplementary Fig. 4c. By measuring more than 100 lines for each size, the distributions of the centre-to-centre distance of the QDs are presented in Supplementary Figs. 4e-4h.

On the other hand, OA-capped and EDT-bridged PbS QDs assemblies form hexagonal arrangements (Supplementary Figs. 4h and 4i, respectively). One QDs has six nearest neighbours, as illustrated by the circle and yellow lines in each TEM image. The distribution of centre-to-centre distance is depicted in the corresponding histogram.

We also analysed the parameters belonging to OA-capped and EDT-bridged PbS QD assemblies with four different variations of QD diameters. The summary of the centre-to-centre distance among QDs with different spacings and shapes (OA-capped, EDT-bridged, and epitaxially-connected PbS QDs assembly) is presented in Supplementary Fig. 4j. The intercept of linear fitting of epitaxially-connected PbS QD-SLs converges to the origin. Meanwhile, the slopes of linear fittings of the EDT-bridged and OA-capped QD assemblies are equal to the slope obtained from the fitting of the epitaxially-connected PbS QD-SLs (slope value close to unity). Nevertheless, the intercepts of the EDT-bridged and OA-capped PbS QD assemblies in the *y*-axis are ~0.5 nm and ~2.5 nm, respectively. These values are comparable to the length of 1,2-ethanedithiol and oleic acid ligand molecules.[1,2]

The centre-to-centre distance among QDs in the epitaxially-connected PbS QD-SLs was further confirmed by GISAXS measurement. The distance was determined from the first Bragg peak of the x-ray scattering pattern (Supplementary Figs. 5a-5d). The position of the peak reflects the distance (or spacing) between two centres of neighbouring QDs d_0 (Supplementary Fig. 5e) through the following relation in Supplementary Equation (1),[3]

$$d_0 = \frac{2\pi}{q_0} \quad (1)$$

The first Bragg peak of scattered x-ray was determined by integrating intensity along the y-axis between q_z = 0 nm^{-1} and q_z = 1 nm^{-1}. The integration results for all QD assemblies are plotted in Supplementary Fig. 5f. The extracted values of the centre-to-centre distance are also shown in each plot. The centre-to-centre distance in the epitaxially-connected PbS QD-SLs from both TEM images and GISAXS data analyses were plotted and compared in Fig. 1c.

In addition, The out-of-plane q_z in the first Bragg peak also contains information about the layer thickness. The intensity profile along the out-of-plane direction of the first Bragg peak is displayed in Supplementary Fig. 5g. For all assemblies with different sizes, the q_z profile displayed a sudden increase of intensity (q_z ~ 0.2 - 0.3 nm^{-1}) and then gradually decayed. This behaviour can be associated with the formation of a single monolayer.[3,4]

The thicknesses of the assemblies were also confirmed by atomic force microscopy (AFM) characterisation (Supplementary Fig. 6). The images was obtained using Hitachi 5100-N in dynamic force microscopy (DFM) mode . The thickness profiles were taken by referencing the surface of the SiO_2 substrate. The obtained thickness values of the assemblies are about the diameter of the corresponding PbS QDs, suggesting the formation of monolayer assemblies. It is consistant with the interpretations of TEM images and out-of-plane analyses of GISAXS patterns.

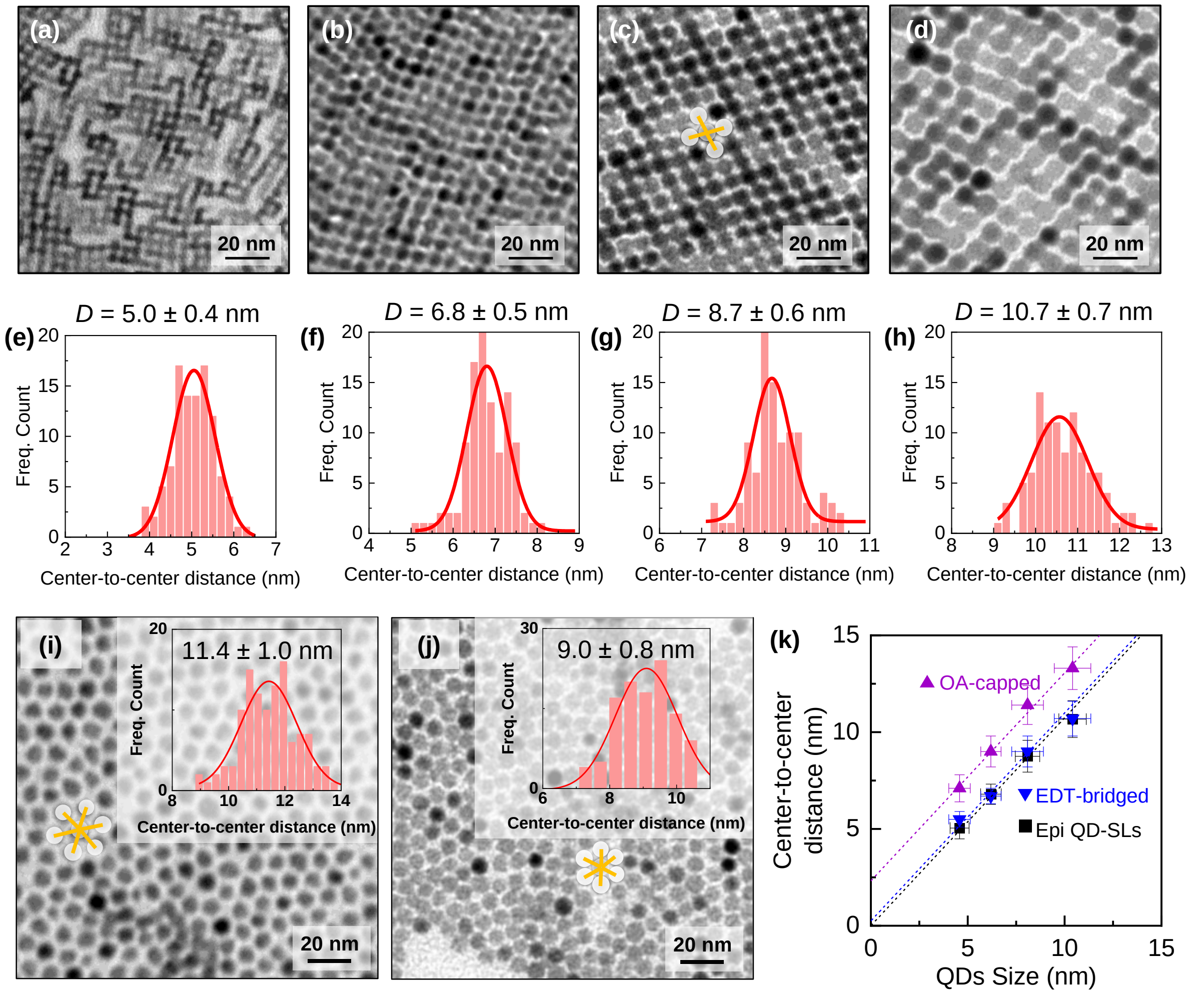

Supplementary Figure 4 | TEM images of epitaxially-connected PbS QD-SLs with diameters of (a) 4.5 nm, (b) 6.2 nm, (c) 8.1 nm, and (d) 10.4 nm. The epitaxially-connected PbS QD SLs show 4 nearest neighbours where the centre-to-centre distance among QDs is determined. The corresponding value is plotted in (e)-(h) from smallest to largest size. (i) and (j) shows the TEM images of OA-capped and EDT-bridged PbS QDs assembly with hexagonal structure, respectively. The centre-to-centre distance among QDs is defined from the six nearest neighbours, and distribution is plotted in the inset figure of each TEM image. The relation between centre-to-centre distance and QDs size is shown in (k). The horizontal and vertical error bars represent the standard deviation.

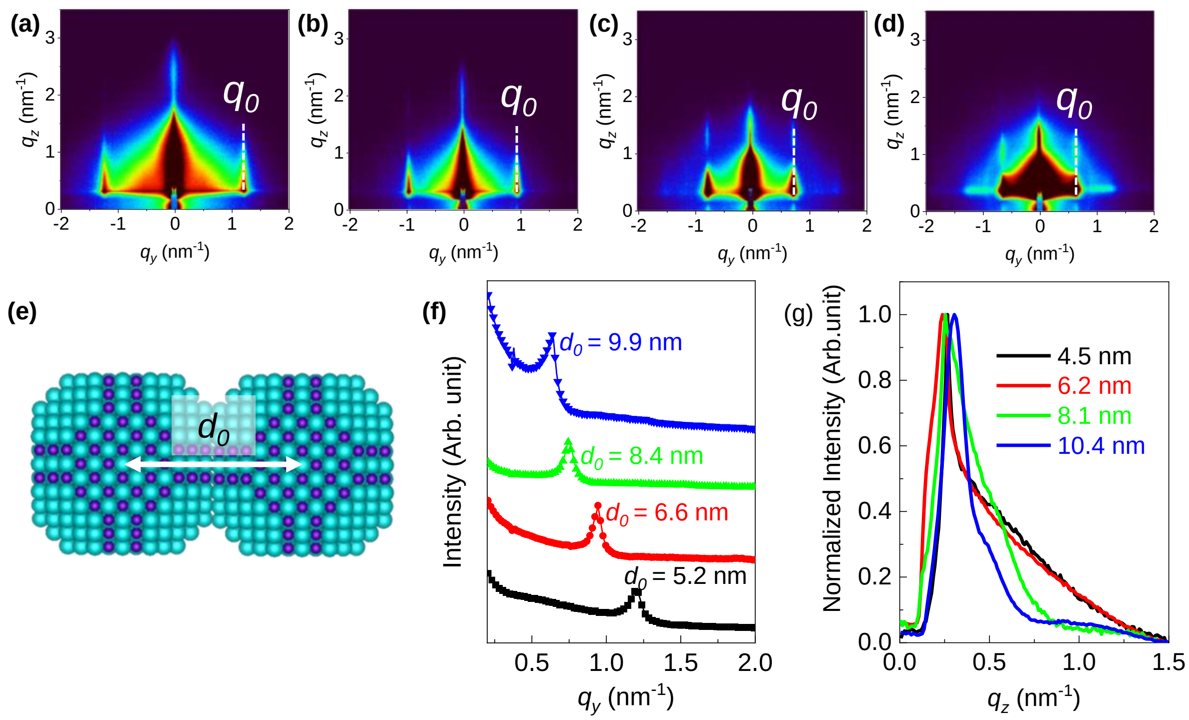

Supplementary Figure 5 | Grazing-Incident Small-Angle X-ray Scattering (GIWAXS) pattern of epitaxially-connected PbS QD-SLs with diameters of (a) 4.5 nm, (b) 6.2 nm, (c) 8.1 nm, and (d) 10.4 nm. (e) illustration of centre-to-centre distance among QDs determined from the scattered x-ray wavevector q_0 of GISAXS data. (f) the in-plane q_y integration of intensity to determine the QDs spacing. (g) the corresponding out-of-plane q_z integration of intensity at the first Bragg peak from GISAXS data.

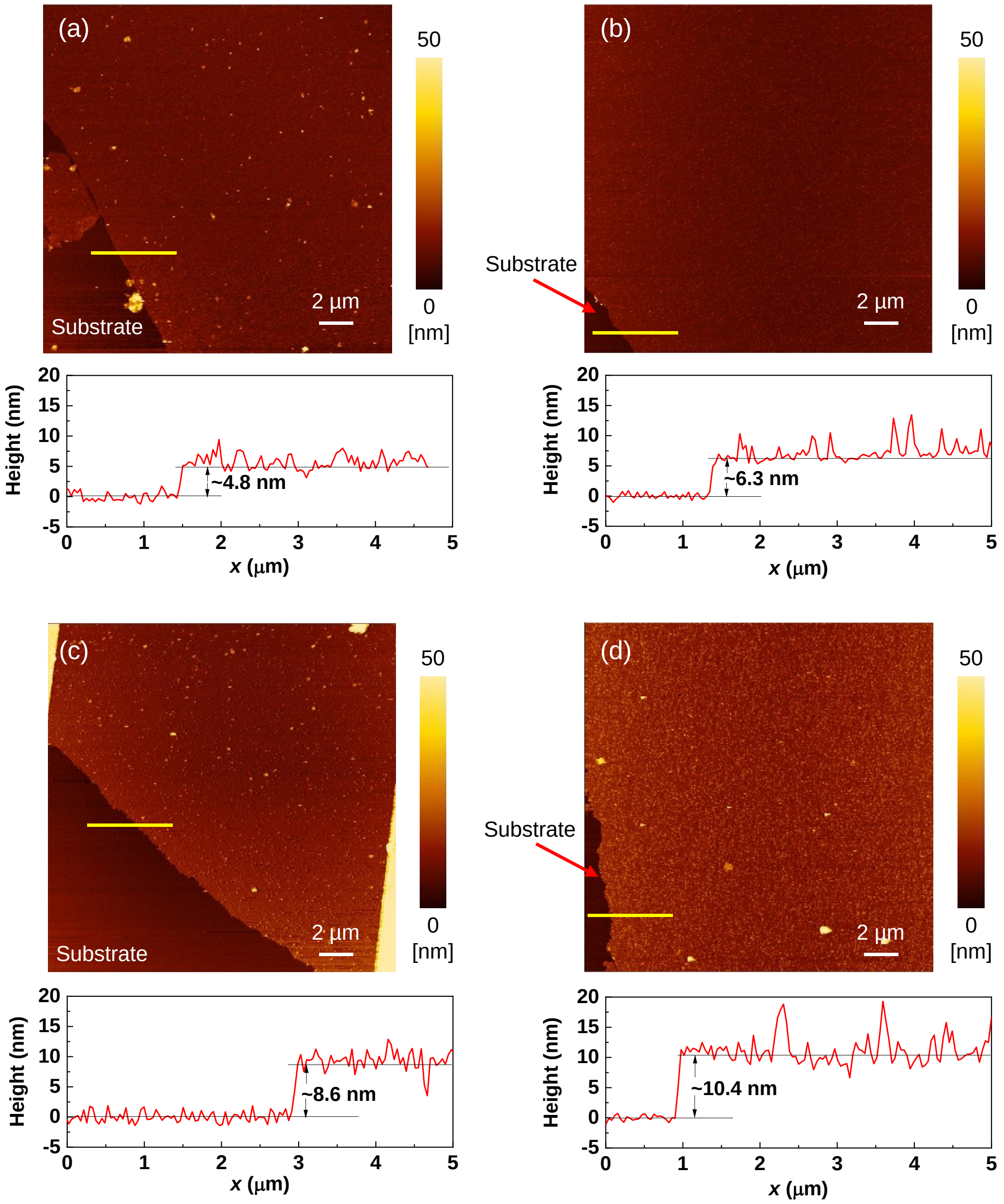

Supplementary Figure 6 | Atomic force microscopy (AFM) images of epitaxially-connected PbS QD-SLs assemblies. The AFM measurements capture monolayer assemblies built from (a) 4.5 nm, (b) 6.2 nm, (c) 8.1 nm, and (d) 10.4 nm diameters of PbS QDs. The corresponding thickness profile is provided below each image by referencing to the surface of the SiO_2 substrates.

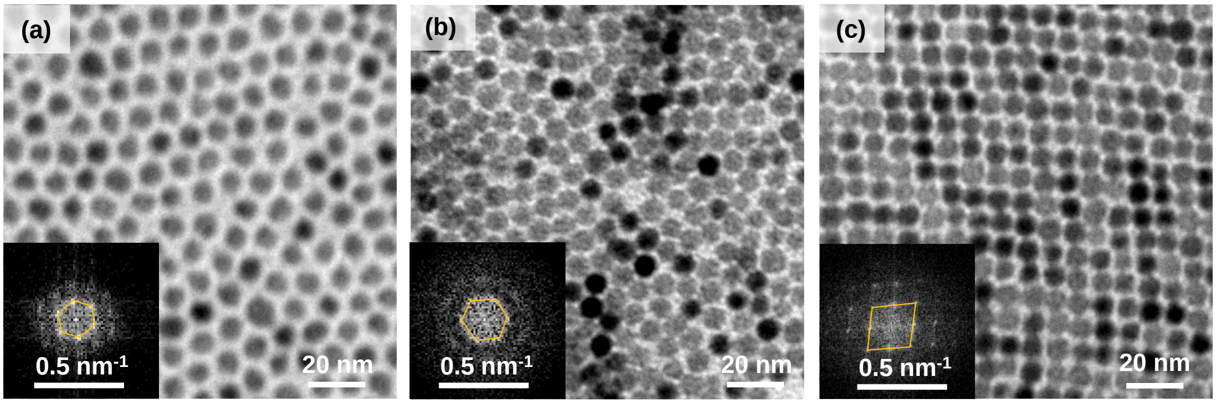

Supplementary Figure 7 | TEM images of (a) OA-capped PbS QDs assembly, showing initial hexagonal shape. (b) Upon ligand exchange using a shorter molecule (i.e., EDT-bridged PbS QDs), the assembly shows a similar hexagonal structure, but the volume shrinks. (c) Controlled ligand stripping transforms the assembly into a rhombus shape of the epitaxially-connected PbS QD-SLs. (insets) Fast Fourier Transform (FFT) of the TEM images. The images are taken from the assemblies of QD with a diameter of 8.1 nm.

Supplementary Note 3: Assembly and Atomic lattice orientation

A. Out-of-plane orientation

GIWAXS data were analysed using FIT2D (European Synchrotron Radiation Facility, ESRF) and MATLAB-based GIXGUI softwares.[5] First, the collected data were corrected from the Ewald sphere (Supplementary Fig. 8a). In order to index the pattern of scattered x-ray, the azimuthal intensity integration was performed in the OA-capped PbS QDs for all sizes, as shown by the shaded area in Supplementary Fig. 8a. The integration intensity of the GIWAXS data transforms from the 2D pattern into a 1D pattern (q). The spline baseline correction was performed in the 1D data, and the corrected versions are plotted in Supplementary Fig. 8b for all PbS QDs. The peak in the 1D data was then matched with d_{hkl} of PbS bulk as the reference. The atomic lattice {111}, {200}, {220}, {311} was found at q ~ 18.3 nm^{-1}, 21.1 nm^{-1}, 29.7 nm^{-1}, and 34.7 nm^{-1} which corresponds to d_{hkl} ~3.4 Å, 3.0 Å, 2.1 Å, and 1.8 Å, respectively. The $\{200\}_{AL}$ further represents {100}, while $\{220\}_{AL}$ is indicating {110} facet. The information of the scattered x-ray wavevector of each atomic lattice, particularly $\{111\}_{AL}$ and $\{200\}_{AL}$, was used further to analyse the orientation of the atomic lattice relative to the substrate (out-of-plane direction).

The orientation of atomic lattice in the epitaxially-connected PbS QD-SLs was confirmed by selected area electron diffraction (SAED) from high-resolution TEM mode and GIWAXS data analysis. The extended SAED and GIWAXS data for all sizes of PbS QDs are displayed in Supplementary Fig. 9. The bright spot electron diffraction patterns were obtained for all samples, and they became more significant in the larger QDs. The atomic lattice is indexed by measuring the distance of the diffraction spots relative to the centre to obtain the lattice spacing d_{hkl}, as shown in Supplementary Figs. 9e-9h.

Furthermore, to determine the atomic orientation of the epitaxially-connected PbS QD-SLs from GIWAXS data, azimuthal line cut-off was performed on the specific scattered x-ray wavevector (**q**) for $\{111\}_{AL}$ and $\{200\}_{AL}$. Here we define 0º as the positive y-axis direction, with the angle incre asing clockwise to the positive x-axis direction, which is 90º. The 0º is associated with the direction parallel to the substrate surface. Otherwise, the azimuthal angle 90º is perpendicular to the substrate surface. The complete data sets of GIWAXS patterns from epitaxially-connected PbS QD SLs with different diameters are depicted in Supplementary Figs. 9i-9l. Like SAED, the GIWAXS data show bright spotted scattered x-ray associated with the

orientation of atomic lattice to the specific direction. The azimuthal line cut-off of the GIWAXS data for $\{111\}_{AL}$ and $\{200\}_{AL}$ are displayed in the main text Figs. 1g and 1h, respectively. The $\{111\}_{AL}$ forms ~56° relative to the substrate, and the extension data (Gaussian fitting) of $\{200\}_{AL}$ shows a peak at 0° where this condition satisfies the reflection of {100} facet face up.

In contrast to epitaxially-connected PbS QD-SLs, both SAED and GIWAXS measurements of OA-capped and EDT-bridged PbS QDs assembly show powder ring-like patterns. These observations suggest that the individual QDs assemble without any specific orientation of the atomic lattice. The data sets of SAED and GIWAXS patterns of OA-capped and EDT-bridged PbS QDs assemblies are presented in Supplementary Figs. 10 and 11, respectively.

B. In-plane Orientation

The assembly orientation in the in-plane direction is determined by superlattice angle α. The superlattice angle α is defined as the smallest angle formed by the three nearest neighbouring QDs, as illustrated in Supplementary Fig. 12. The hexagonal structure shows a superlattice angle α = 60° (Supplementary Fig. 12a), while a perfect square lattice has a superlattice angle α = 90° (Supplementary Fig. 12c). We define the 2D rhombic structure of the assembly as a condition when the superlattice angle α is between 60° and 90° (Supplementary Fig. 12b). The measurement of superlattice angle α was conducted to more than 100 measured points from each TEM image of OA-capped (Supplementary Figs. 13a-13d), EDT-bridged (Supplementary Figs. 13e-13h), and epitaxially-connected PbS QD-SLs (Supplementary Figs. 13i-13l). The histogram of measured superlattice angle α distribution is plotted at the bottom of the corresponding TEM image. The average value of superlattice angle α is plotted in Fig. 2c, main text.

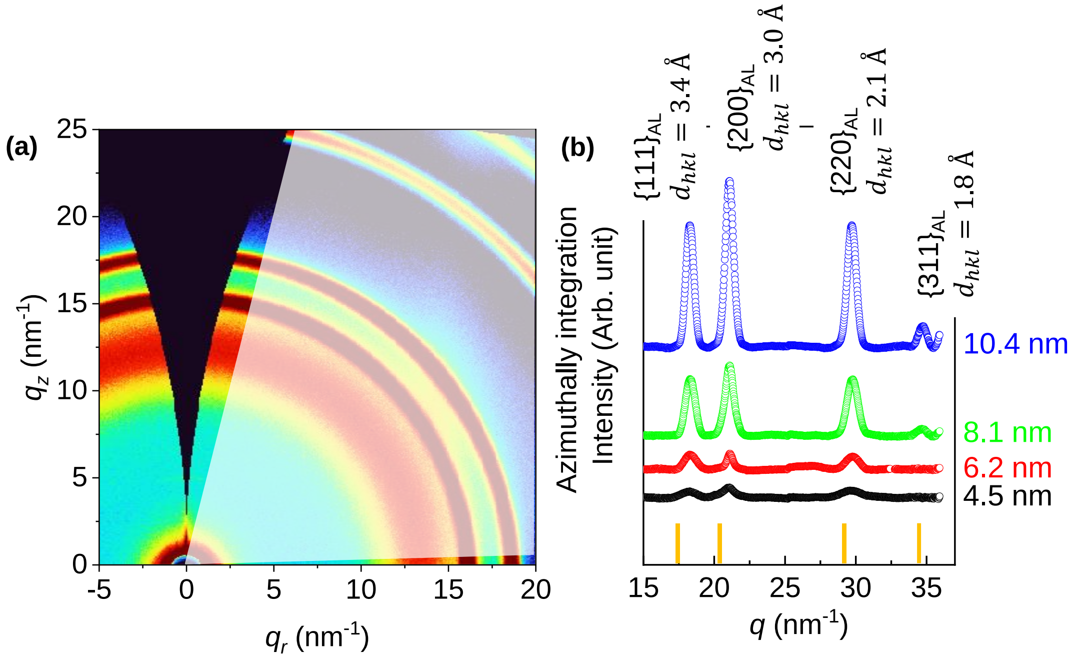

Supplementary Figure 8 | Grazing Incident Wide Angle X-ray Scattering (GIWAXS) pattern index taken from OA-capped PbS QD assembly. (a) Azimuthal integration of GIWAXS pattern over the shaded area. (b) The 1D transformation of the GIWAXS pattern obtained from angular integration shows the peak at specific scattered x-ray wavevector q. The peak positions match those of the bulk PbS crystal.[6]

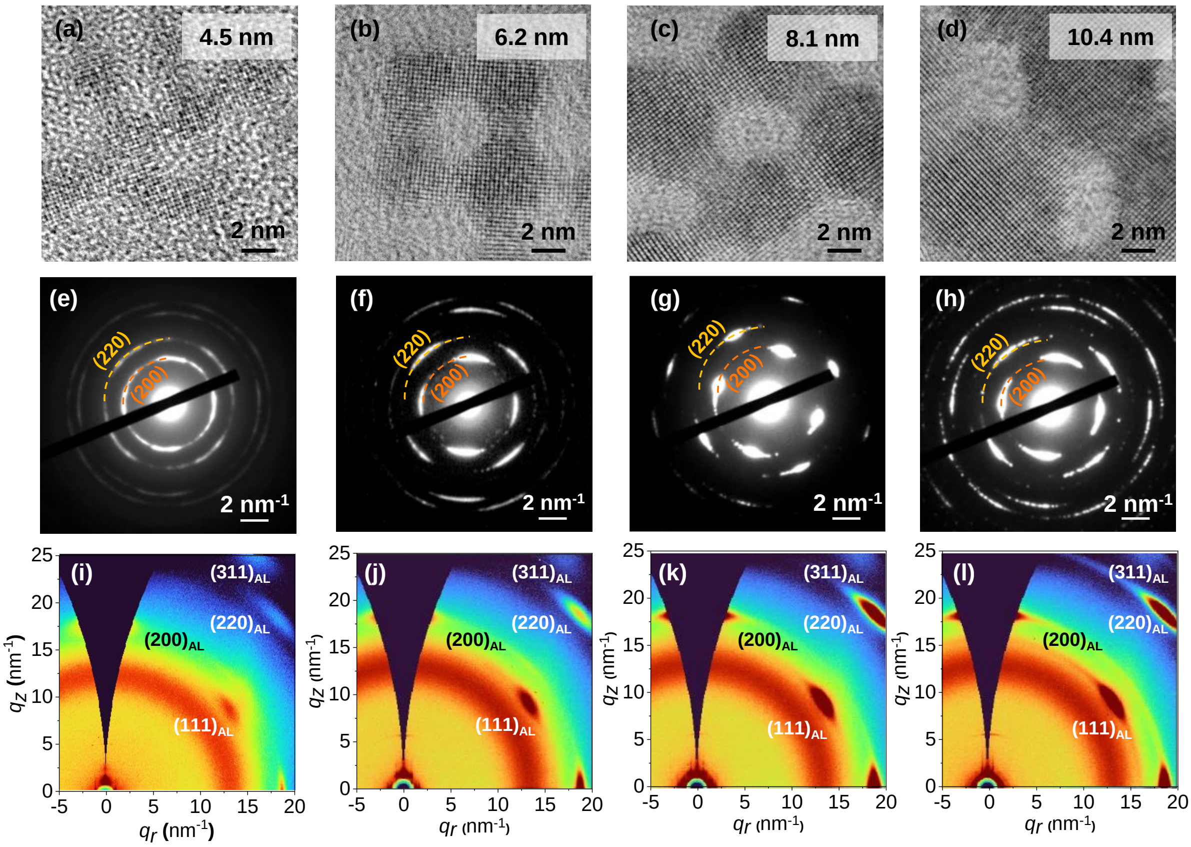

Supplementary Figure 9 | Atomic lattice orientation in epitaxially-connected PbS QD-SLs. (a)-(d) High-resolution TEM images, (e)-(h) selected area electron diffraction of the corresponding HR-TEM, (i)-(l) GIWAXS pattern of the superlattices. The diameters of PbS QDs are 4.5 nm, 6.2 nm, 8.1 nm, and 10.4 nm from the left to the right images.

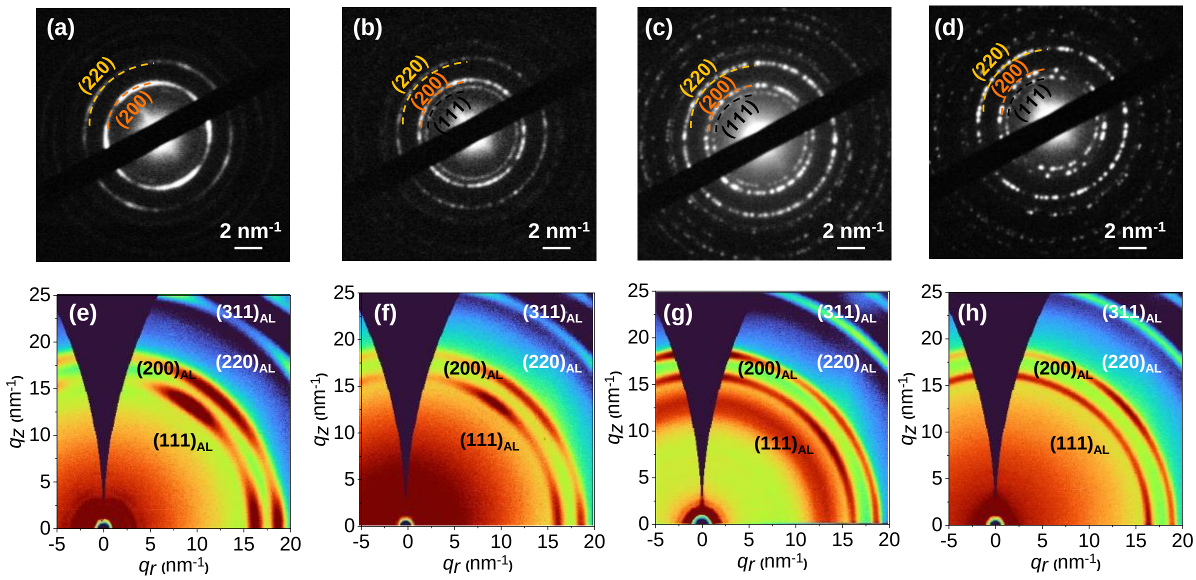

Supplementary Figure 10 | Atomic lattice orientation in oleic acid (OA)-capped PbS QDs assembly. (a)-(d) Selected area electron diffraction (SAED), (e)-(h) GIWAXS pattern of the assemblies. The diameters of PbS QDs are 4.5 nm, 6.2 nm, 8.1 nm, and 10.4 nm from the left to the right images.

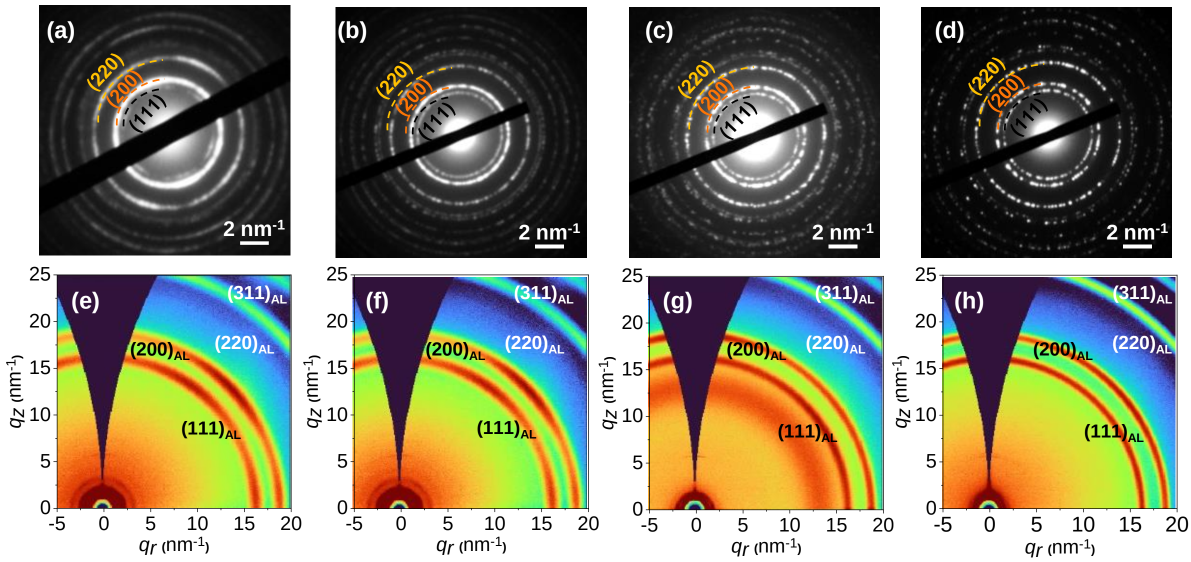

Supplementary Figure 11 | Atomic lattice orientation in 1,2-ethanedithiol (EDT)-bridged PbS QDs assembly. (a)-(d) Selected area electron diffraction (SAED), (e)-(h) GIWAXS pattern of the assemblies. The diameters of PbS QDs are 4.5 nm, 6.2 nm, 8.1 nm, and 10.4 nm from the left to the right images.

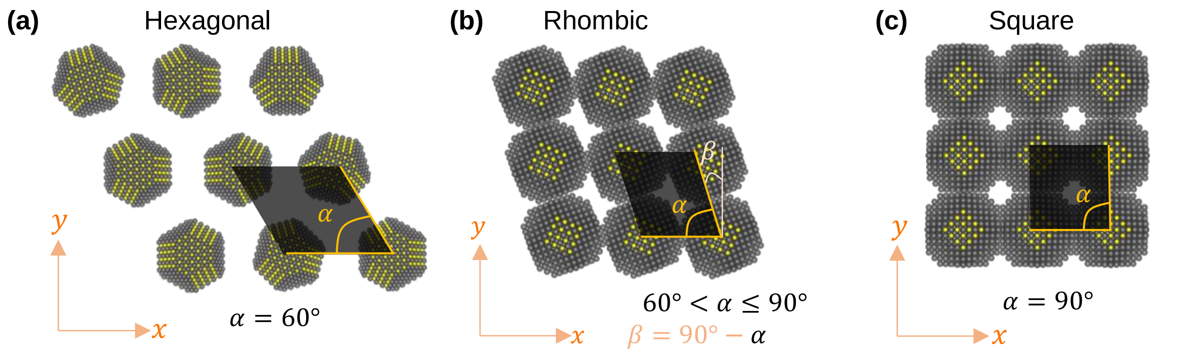

Supplementary Figure 12 | Assembly and atomic lattice orientations in the in-plane direction. Illustration of (a) hexagonal, (b) rhombic, and (c) square shape of QDs assembly where the atomic lattice orientation is following the superlattice angle α, and the tilted atomic lattice angle β is demonstrated in rhombic shape relative to the square shape as reference.

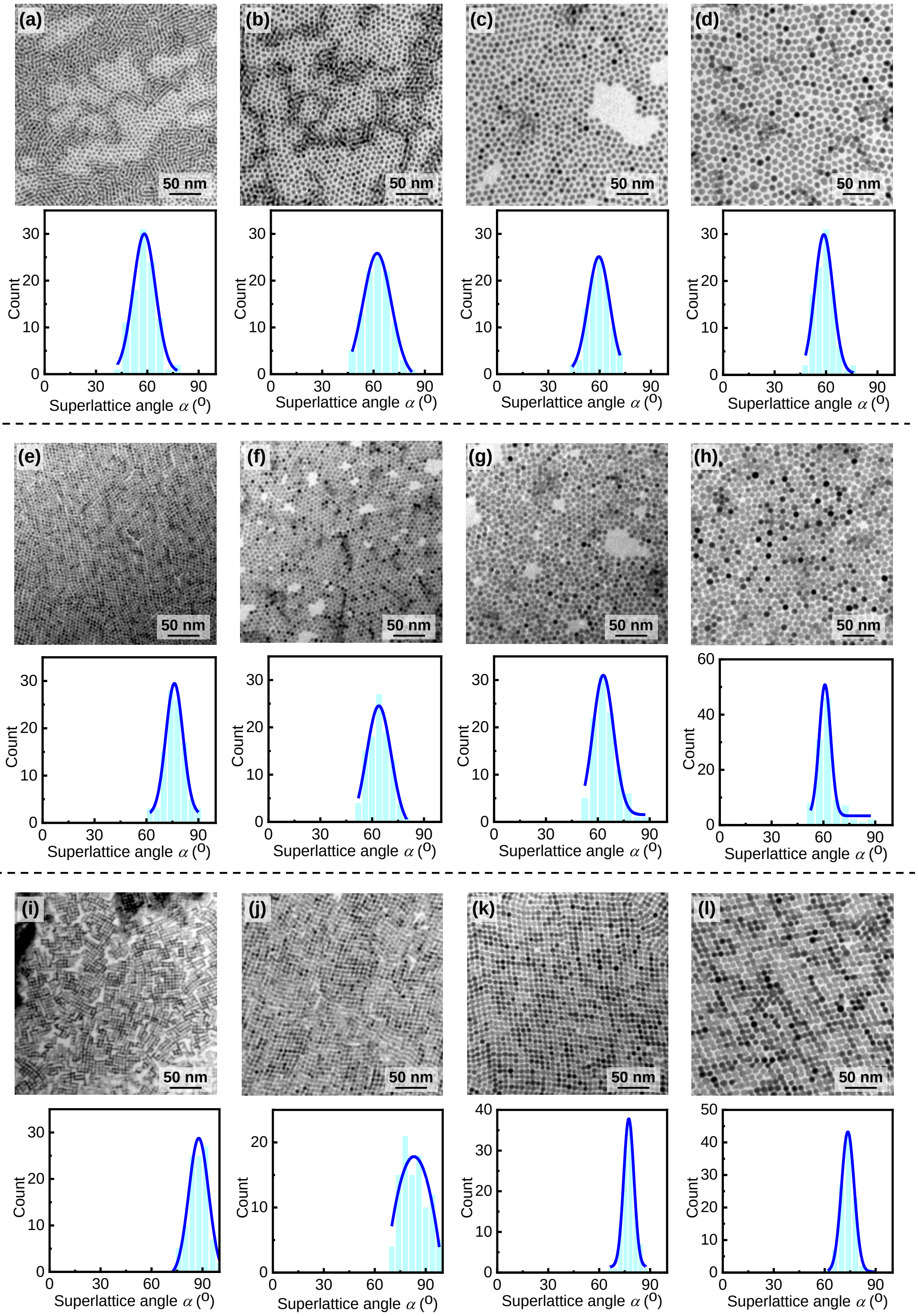

Supplementary Figure 13 | Assembly and atomic lattice orientation in the in-plane direction. The TEM images and the deduced superlattice angle α distribution of (a)-(d) OA-capped PbS QD assemblies, (e)-(h) EDT-bridged PbS QD assemblies, and (i)-(l) epitaxially-connected PbS QD-SLs. The diameters of PbS QDs are from 4.5 nm to 10.4 nm from the left to the right images.

Supplementary Note 4: Room Temperature Electronic Transport Measurement

A. Transport Measurement using FET

The room temperature electronic transport measurements were performed using a two-terminal FET configuration. The FETs are characterised under two measurement modes: I_D-V_D output and I_D-V_G (or V_{ref}) transfer characteristics. All I_D-V_G transfer characteristics were taken from the linear regime to exclude the influence of the minority carriers in mobility determination. The I_D-V_D output characteristics of epitaxially-connected PbS QD-SLs (Supplementary Figs. 14a-14d) were used to determine the linear regime. The *n*-channel (electron enhancement scan) characteristics are plotted as blue lines ($V_G \geq 0$ and $V_D \geq 0$), while the *p*-channel (hole enhancement scan) characteristics are plotted as red lines ($V_G \leq 0$ and $V_D \leq 0$). For the carrier mobility determination, we only focused on the *n*-channel measurement. The linear regime on the *n*-channel is found at $V_D \leq 0.2$ V. Therefore, all I_D-V_G transfer curves presented here were taken at $V_D = 0.05$ V.

Supplementary Figs. 15a-15d replot the transfer I_D-V_G (V_{ref}) of the epitaxially-connected PbS QD-SLs of different diameters, both in logarithmic and linear scales. All devices showed small hysteresis of the forward-backwards scans, indicating an effective gating modulation. The ON/OFF modulation ratio is defined as the ON current value (at maximum V_{ref}) divided by the lowest OFF current. We obtained the highest ON/OFF ratio values of more than 10^5 (Supplementary Fig. 15e).

Furthermore, the subthreshold swing (*SS*) values were extracted from the logarithmic plot of I_D-V_G transfer characteristics in the subthreshold region (the region between off current and threshold voltage) using the following Supplementary Equation (2),

$$SS = \left(\frac{\partial \log(I_D)}{\partial V_G}\right)^{-1} \tag{2}$$

Minimum *SS* values, close to the limit of trap-less charge carrier transport (~60 mV dec^{-1}), were obtained for all samples (Supplementary Fig. 15e, bottom panel).[7] These observations indicate an effective filling of the trap states by ionic liquid gating.[8] Consequently, the intrinsic properties of QD-size-dependent charge carrier transport can be fairly evaluated.

For a fair comparison, the FETs of the EDT-bridged PbS QDs assemblies were also measured under identical measurement parameters. The I_D-V_D output and I_D-V_G transfer characteristics of the devices are shown in Supplementary Figs. 17 and 18, respectively.

The ionic liquid gating technique can be employed to determine the edge of the electronic state (valence and conduction band-edge) due to the high capacitance (geometrical capacitance, C_G) of the ionic liquid.[9] The band-edge (or electronic bandgap) of the semiconductor can be extracted by determining the threshold voltage of the p- and n-channel. We extracted the threshold voltage from the linear intercept of transconductance ($g_m = \partial I_D / \partial V_{G,Ref}$) slope with the x-axis (Supplementary Fig. 20a).[10] From these threshold voltage determinations for hole and electron accumulations, we can define the electronic bandgap of the probed semiconductor as the difference between the threshold voltage of p- and n-channel ($e\Delta V_{th} = eV_{th,e} - eV_{th,h}$) (Supplementary Fig. 20b). The probed electronic bandgap showed size-dependency where the larger size of QDs shows narrower bandgap (Supplementary Fig. 20c). This determination revealed that quantum confinement effect of the QDs is preserved although the QDs are epitaxially-connected. It is also confirmed by the corresponding absorption spectra (Supplementary Fig. 21), where the excitonic peak still can be clearly observed.

B. Capacitance Measurement and Electron Mobility Determination

Unlike solid-state dielectric gating, the capacitance value on the EDLT is not constant for the entire applied gate voltage range. EDLT capacitance increases as the applied gate voltage increase, which means that the charge carrier accumulation is not linearly proportional to the gate voltage. Consequently, the calculation of charge carrier mobility could be overestimated when any assumption of constant capacitance value is used. In order to determine the charge carrier mobility, the accumulated charge carrier density should be precisely deduced by measuring the gate-dependent capacitance of the system using electrochemical-impedance-spectroscopy (EIS) measurement. The EIS was performed in potentiostat mode on the identical devices we used for FET measurement. 10 mV AC voltage (RMS) was applied between the gate and PbS QDs under variable frequencies ranging from 10 kHz to 0.05 Hz, while a DC gate voltage was also applied. The measured impedance Z consists of the real (Z') and imaginary (Z") parts. The areal capacitance value was deduced from the imaginary part of impedance (Z") using the following Supplementary Equation (3),[11]

$$C = \frac{1}{A} \cdot \frac{(-1)}{2\pi f Z''} \tag{3}$$

where A is the active area of PbS QDs covered by ionic liquid and f is the frequency of the AC voltage. The gate voltage was applied from -1 V to 1.5 V. The properties of the measurements are plotted as bode-phase (Supplementary Fig. 22a) and C-f (Supplementary Fig. 22b). We determined the capacitance value at 1 Hz, a frequency value equivalent to the sweeping rate of gate voltage used in FET transport measurement. For instance, Supplementary Fig. 22c (left y-axis) showed the capacitance value of the EDT-bridged PbS QD assemblies (blue dots) and the epitaxially-connected PbS QD-SLs (red dots). Both were assembled from QD with diameters of 8.1 nm.

Furthermore, the gate-dependent accumulated charge carrier density (n) can be determined from the gate voltage-dependent capacitance using Supplementary Equation (4)

$$n(V_G) = \frac{1}{e} \cdot \int_0^{V_G} C(V_G) \cdot dV_G \tag{4}$$

Supplementary Fig. 22c (right y-axis) shows the deduced gate-dependent electron density.

The electron mobility μ was then determined from the measured conductance and estimated electron density, Supplementary Equation (5),[8]

$$\mu = \frac{L_{CH}}{e \cdot W_{CH} \cdot V_{DS}} \cdot \frac{I_D}{n} \tag{5}$$

where L_{CH} is channel length, W_{CH} is channel width, and V_{DS} is the drain-source voltage. I_D and n are the measured drain current of the FET and the deduced accumulated electron density from the EIS measurement, respectively. Supplementary Fig. 22d shows the charge-density-dependent electron mobility obtained from two different kinds of assemblies.

Supplementary Fig. 23 compares the characteristics of electron mobility at different applied gate voltage values of FETs built from QD with different diameters. Figure 3d (main text) only plots the highest mobility values representing each device at the highest applied gate voltage.

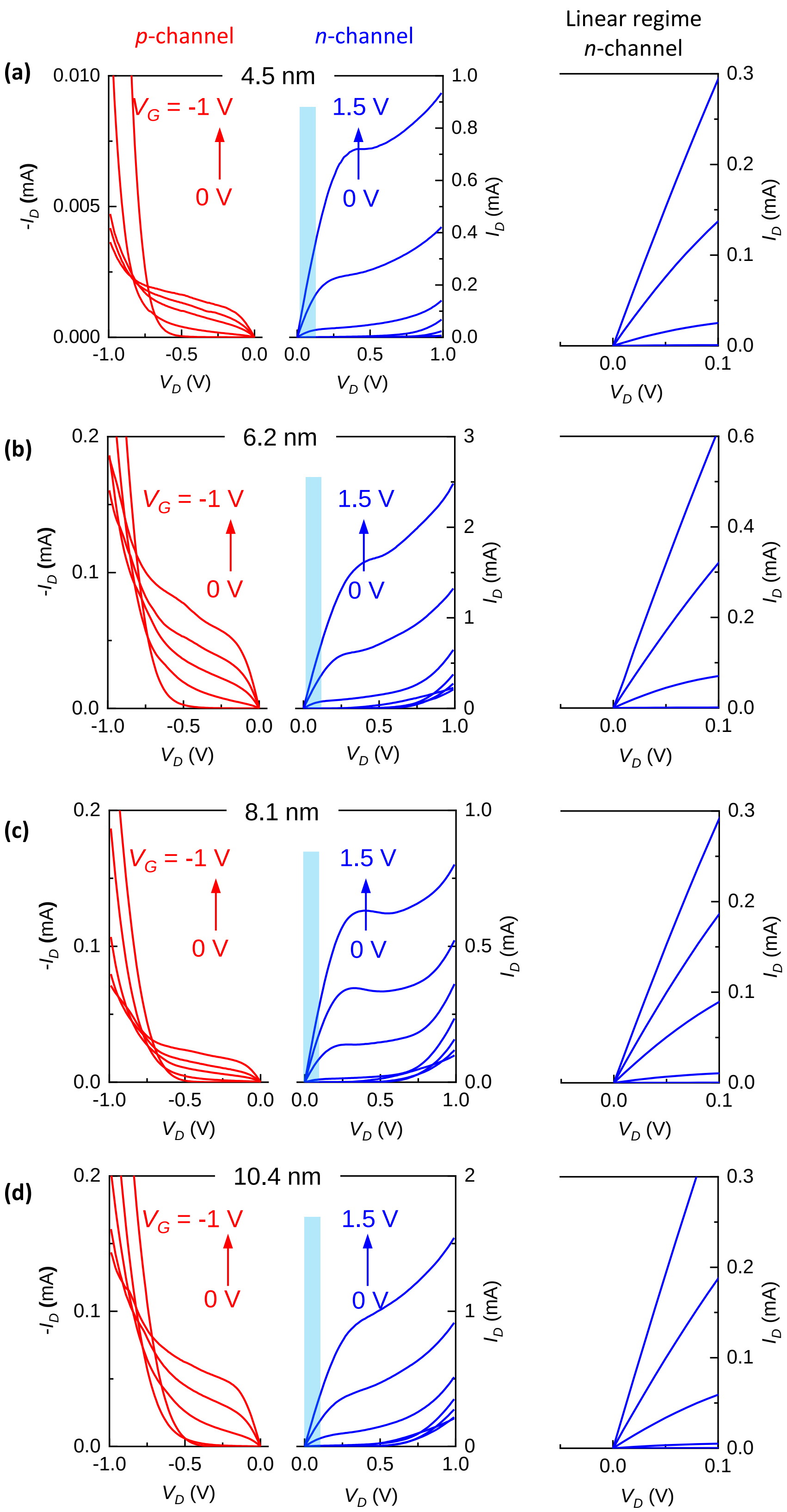

Supplementary Figure 14 | I_D-V_D output characteristics of epitaxially-connected PbS QD-SLs ionic liquid gating FET with QDs size of (a) 4.5 nm, (b) 6.2 nm, (c) 8.1 nm, and (d) 10.4 nm. The rightmost panels display the linear regime of the n-channel operation.

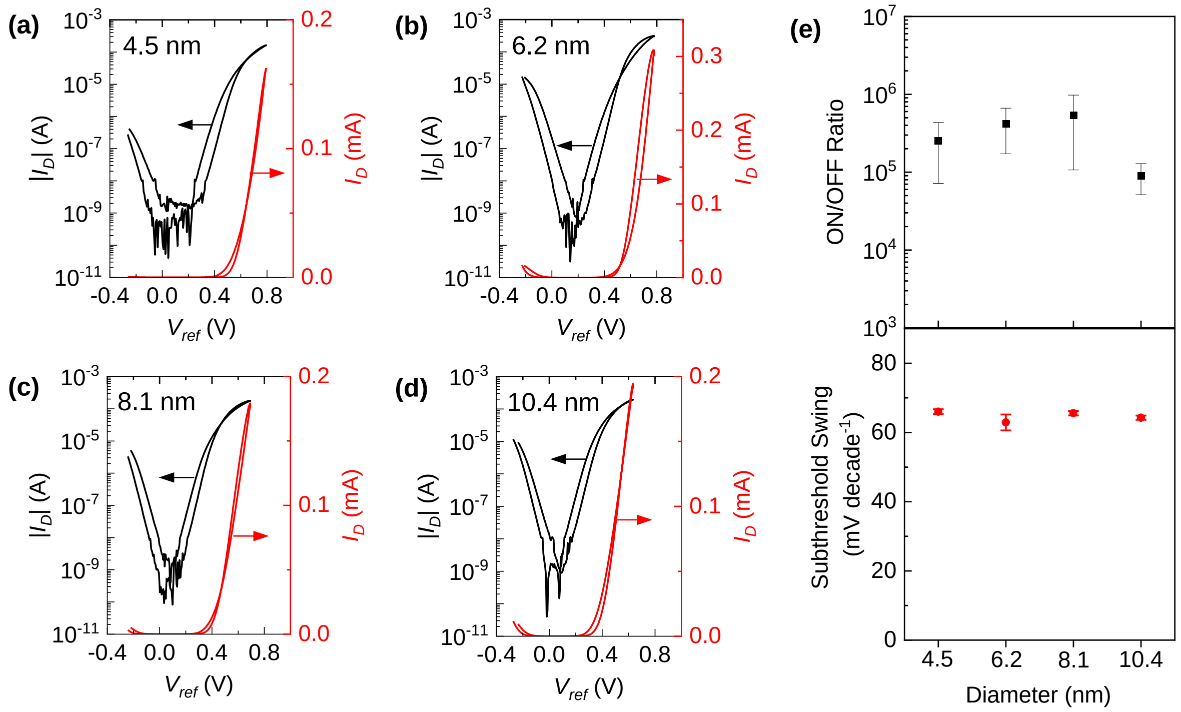

Supplementary Figure 15 | *I_D-V_G* transfer characteristics of epitaxially-connected PbS QD-SLs ionic liquid gating FET with QDs size of (a) 4.5 nm, (b) 6.2 nm, (c) 8.1 nm, and (d) 10.4 nm. The term V_{ref} (measured reference voltage) substitutes the V_G in ionic liquid gating transfer characteristics to better represent the actual carrier accumulation. The transfer characteristics were obtained from the linear regime (V_D = 50 mV). (e) The ON/OFF ratio values (top panel) were obtained from the ratio between on current value and off current value, and the subthreshold swing values (bottom panel) were obtained from linear fitting of the I_D (logarithmic plot) at the vicinity of the subthreshold regime. The vertical error bars represent standard deviation.

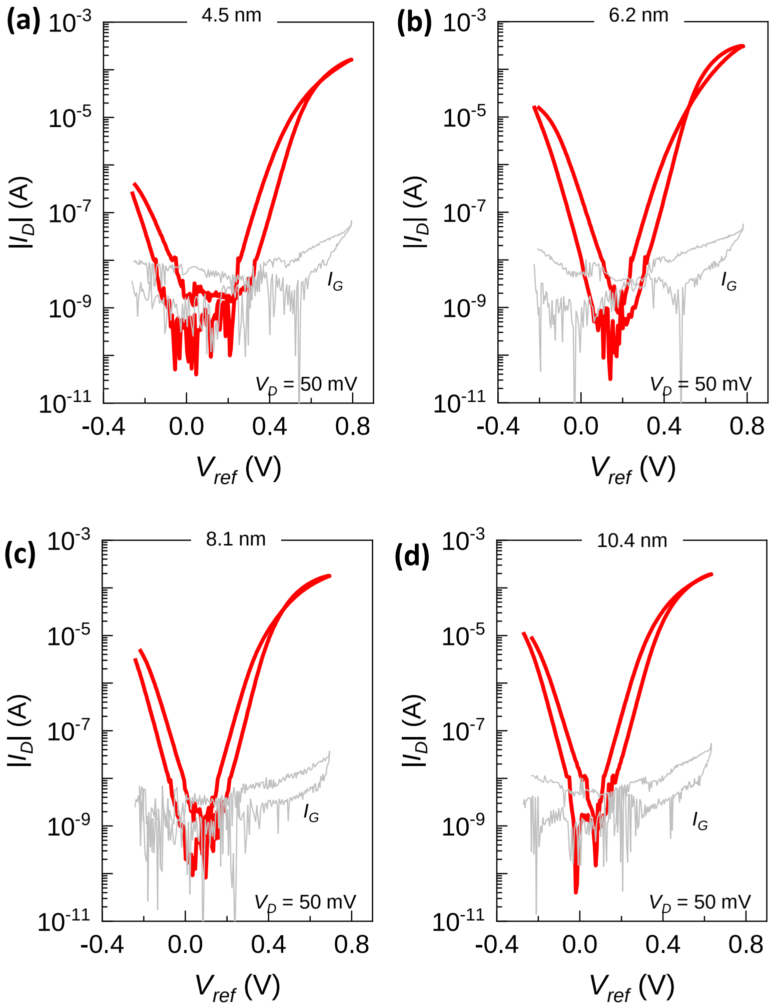

Supplementary Figure 16 | Details of I_D-V_G transfer characteristics and leak current comparison of the epitaxially-connected QD-SL FETs. The logarithmic plot of I_D-V_G and the corresponding leak current (I_G) level (in grey line) of epitaxially-connected PbS QD-SLs under ionic liquid gating with size (a) 4.5 nm, (b) 6.2 nm, (c) 8.1 nm, and (d) 10.4 nm.

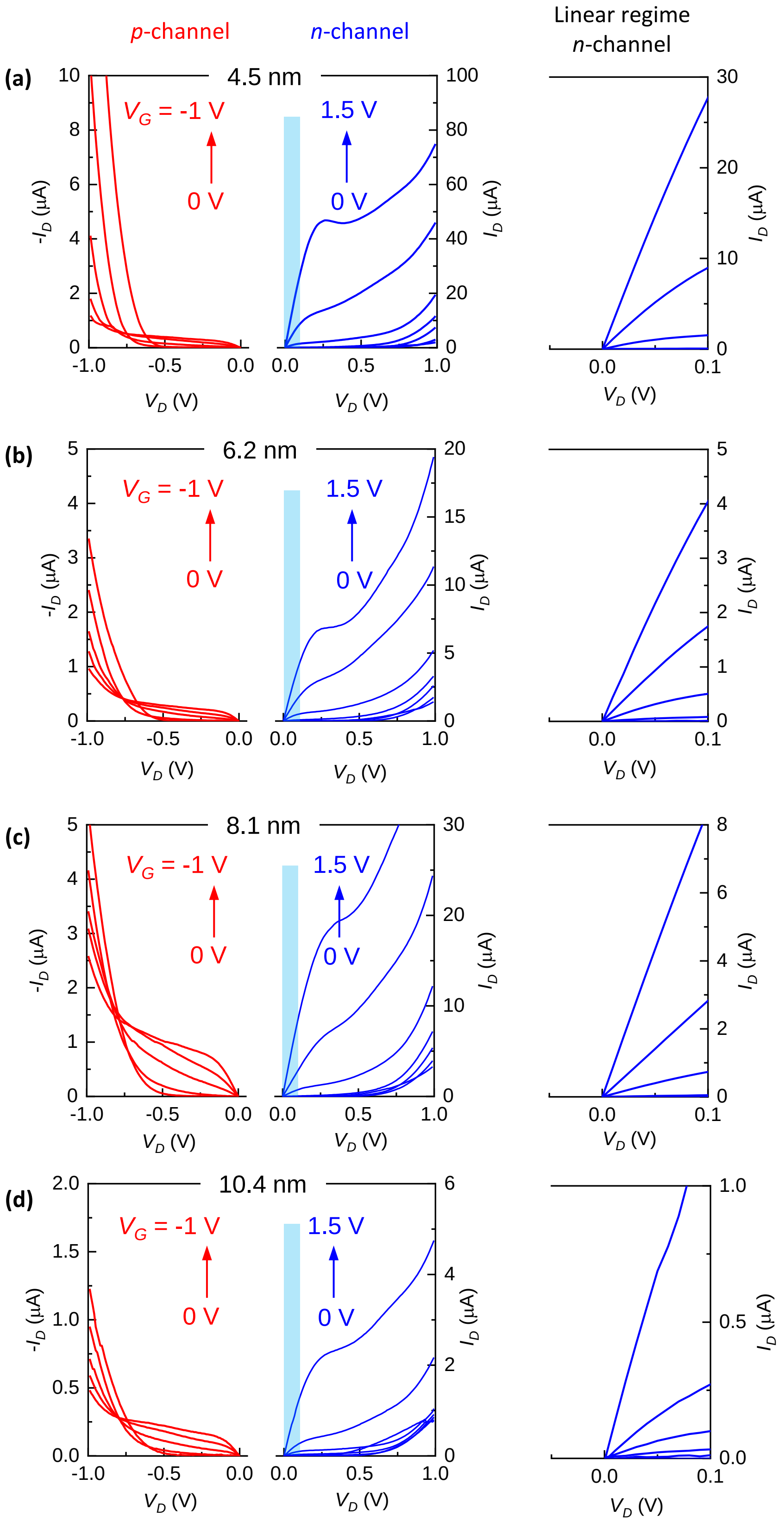

Supplementary Figure 17 | I_D-V_D output characteristics of 1,2-Ethanedithiol (EDT)-capped PbS QDs assembly ionic liquid gating FET with QD diameters of (a) 4.5 nm, (b) 6.2 nm, (c) 8.1 nm, and (d) 10.4 nm. The rightmost panels display the linear regime of the n-channel operation.

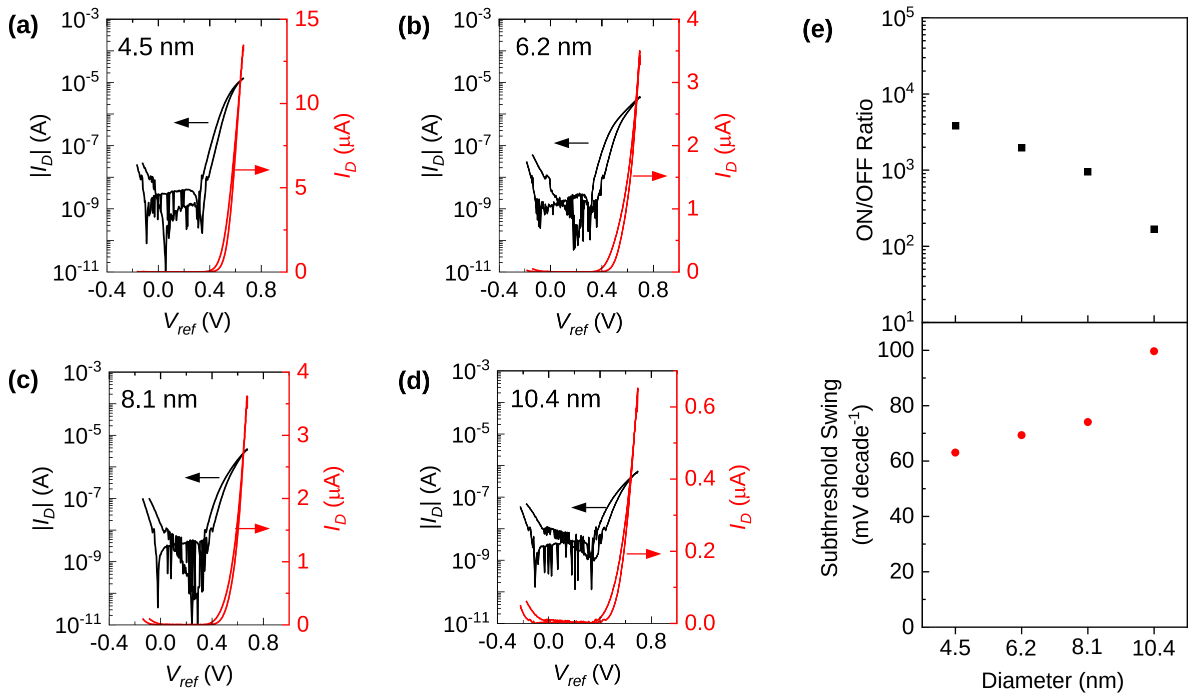

Supplementary Figure 18 | I_D-V_G transfer characteristics of 1,2-Ethanedithiol (EDT)-bridged PbS QDs assembly ionic liquid gating FET with QDs size of (a) 4.5 nm, (b) 6.2 nm, (c) 8.1 nm, and (d) 10.4 nm. The transfer characteristics were obtained from the linear regime (V_D = 50 mV). (e) The ON/OFF ratio values (top panel) were obtained from the ratio between on current value and off current value, and the subthreshold swing values (bottom panel) were obtained from linear fitting of the I_D (logarithmic plot) at the vicinity of the subthreshold regime. The vertical error bars represent standard deviation.

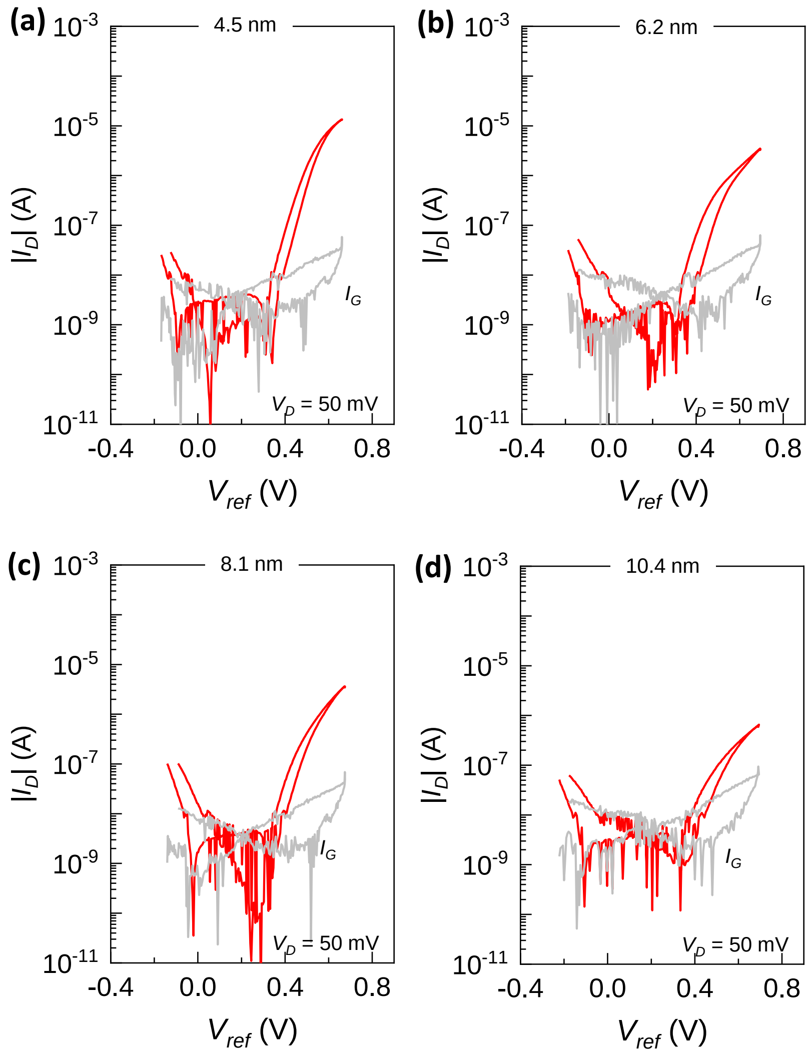

Supplementary Figure 19 | Details of I_D-V_G transfer characteristics and leak current comparison of the ligand-connected QD assembly FETs. The logarithmic plot of I_D-V_G and the corresponding leak current (I_G) level (in grey line) of short ligand EDT-bridged PbS QD superlattice assembly under ionic liquid gating with size (a) 4.5 nm, (b) 6.2 nm, (c) 8.1 nm, and (d) 10.4 nm.

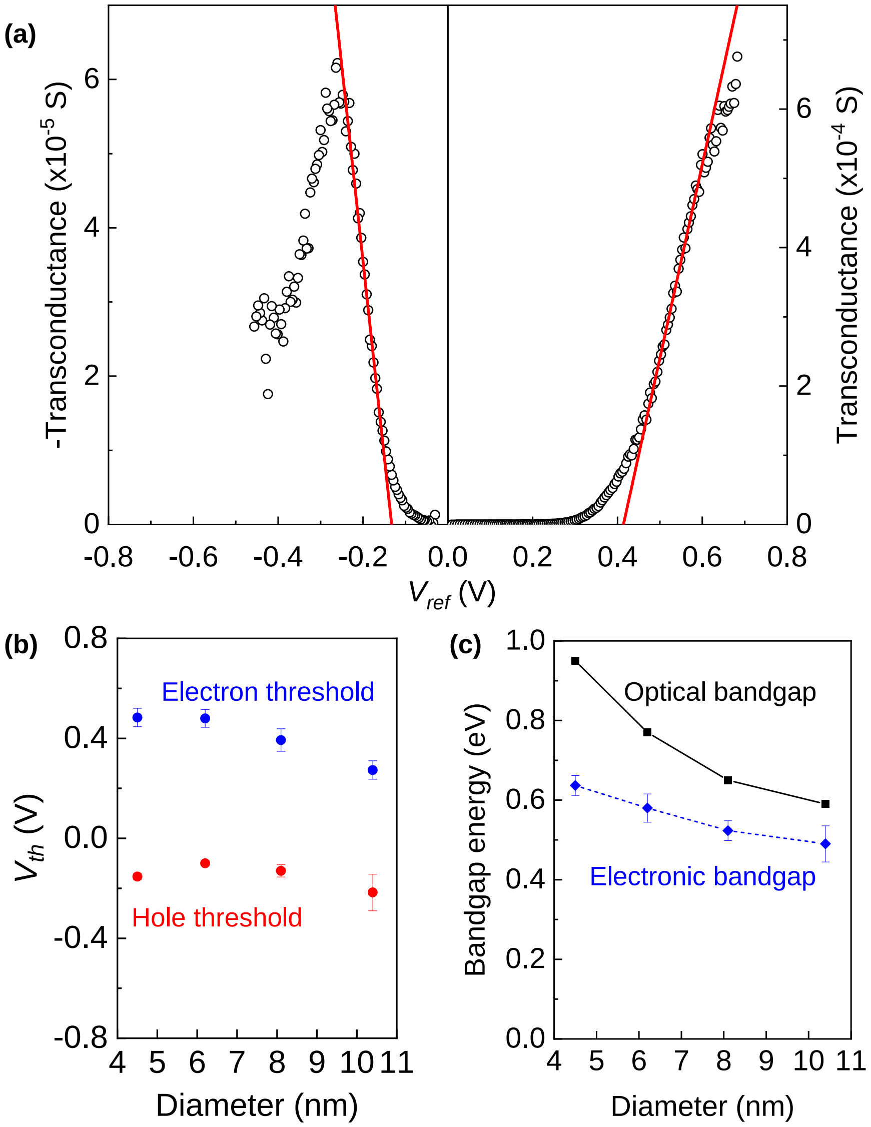

Supplementary Figure 20 | Electronic bandgap and threshold voltage determination of epitaxially-connected PbS QD-SLs. (a) The threshold voltage is determined from the intercept of linear fitting of the transconductance g_m at the *x*-axis. (b) The plot of electron and hole threshold voltage, and (c) the comparison of electronic bandgap from threshold voltage and optical bandgap from absorption spectra measurement. The error bars on threshold voltage and electronic bandgap represent the standard deviation.

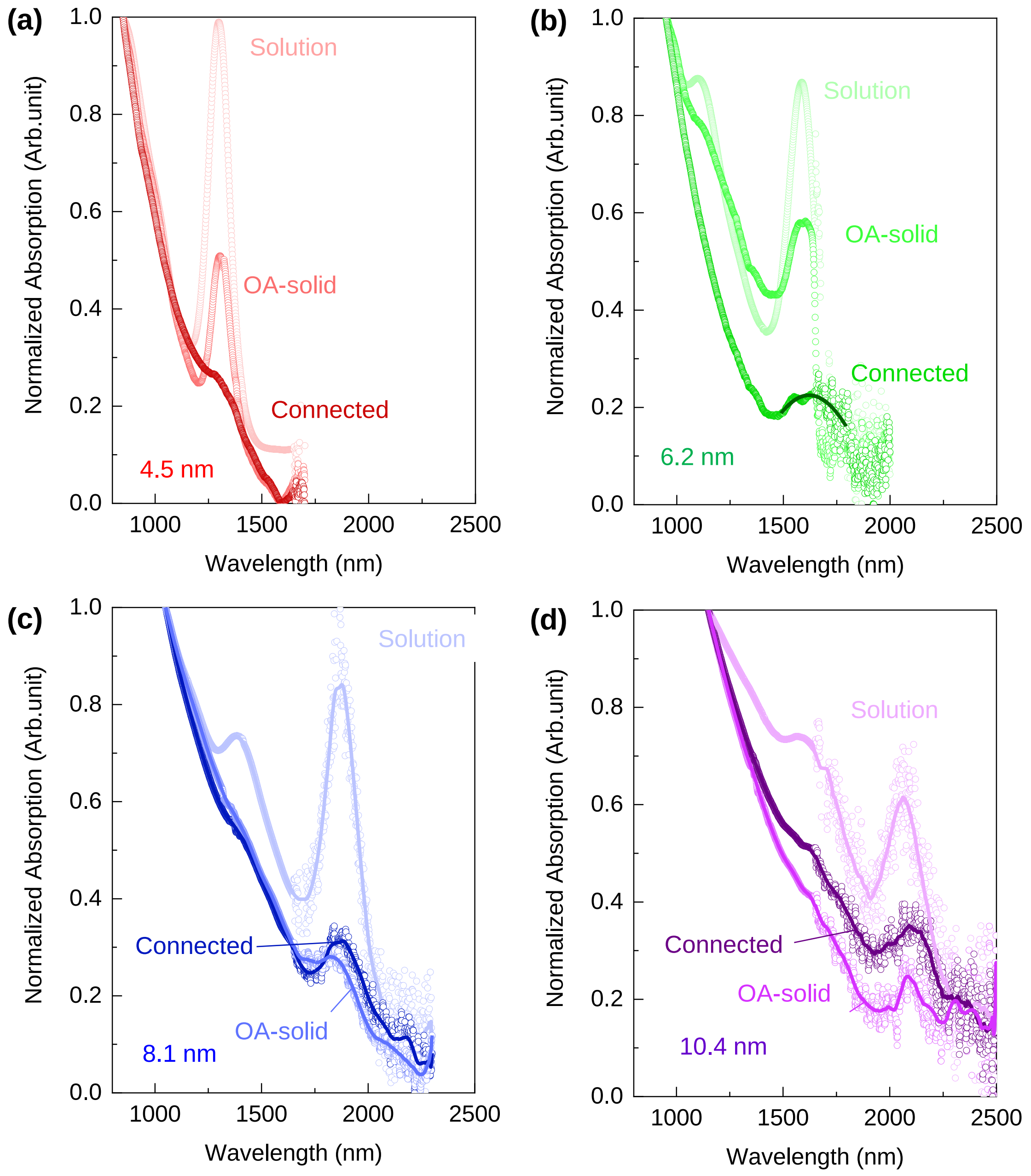

Supplementary Figure 21 | Absorption spectra of epitaxially-connected PbS QD-SLs with QD diameters of (a) 4.5 nm, (b) 6.2 nm, (c) 8.1 nm, and (d) 10.4 nm in comparison to the absorption of their solutions and their oleic acid-capped QD assemblies. Both the epitaxially-connected QD-SL samples and oleic-acid capped QD assembly samples were prepared as one single monolayer. The absorption spectra in the region above 1650 nm is noisier due to the difference of the sensitivity and the background noise of the detectors in the spectrophotometer. A cooled InGaAs detector was used for the measurement below 1650 nm and a PbS detector was used for the measurement above 1650 nm.

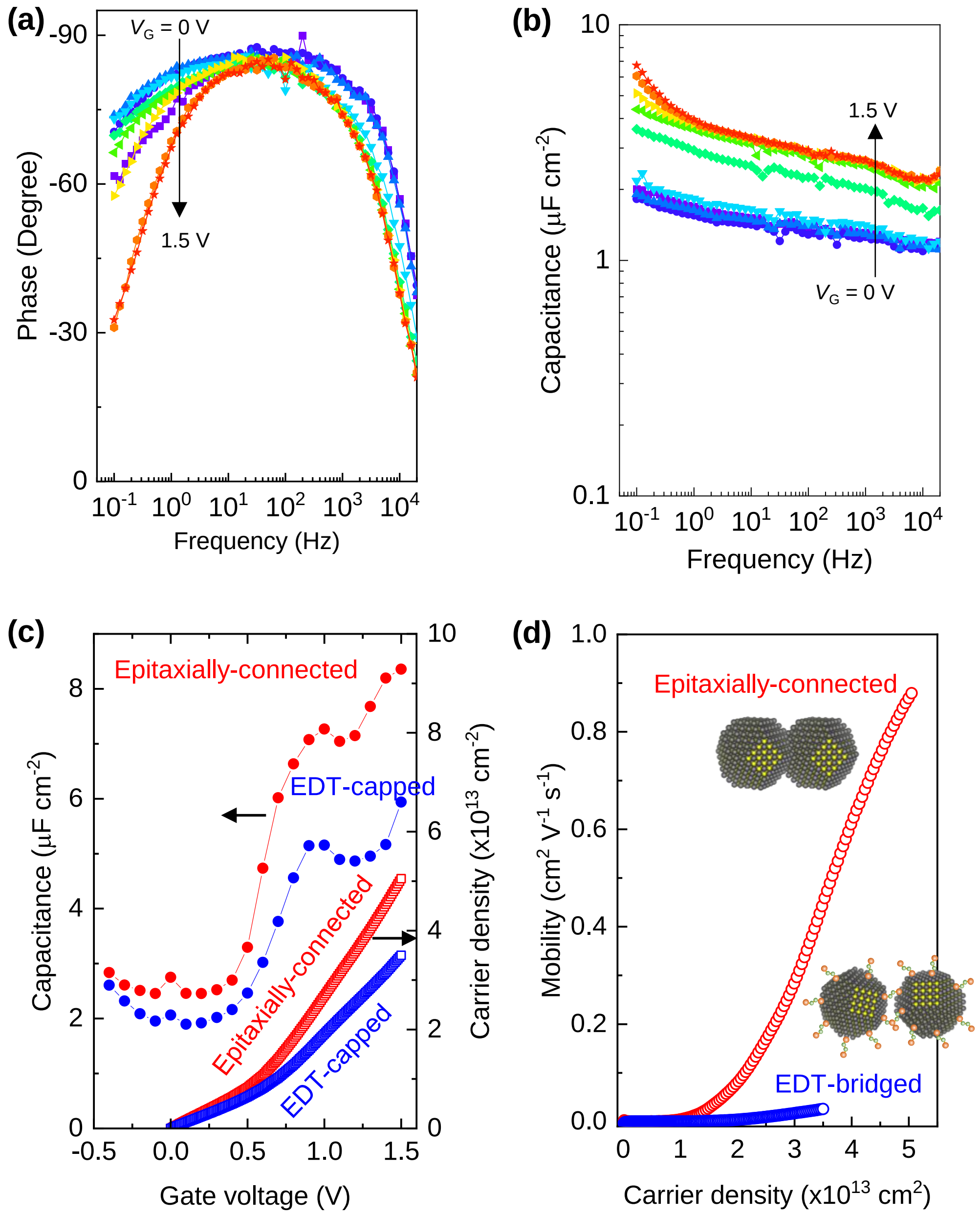

Supplementary Figure 22 | Capacitance measurement and determination of charge carrier accumulation density. (a) Bode-phase and (b) *C-f* profile of PbS QDs/ionic liquid/platinum sandwich system. The profiles were obtained from the electrochemical impedance spectroscopy (EIS) measurement. (c) The corresponding capacitance value as a function of applied gate voltage for epitaxially-connected and EDT-bridged PbS QDs assembly. The capacitance is deduced from *C-f* profile at 1 Hz AC voltage. The carrier accumulation is determined by capacitance integration over gate voltage from 0 V to 1.5 V for electron accumulation. (d) Plots of the obtained electron mobility as a function of the accumulated electron in both EDT-bridged and epitaxially-connected PbS QD-SLs.

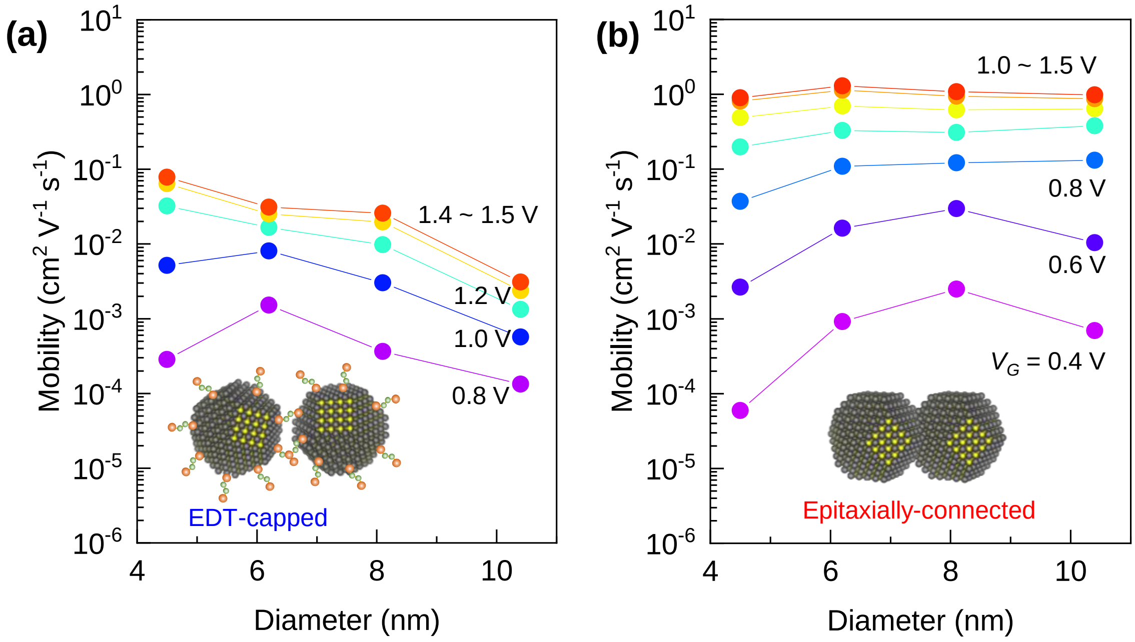

Supplementary Figure 23 | Gate-dependent electron mobility of QD assemblies built from different QD diameters. Plots of the characteristic electron mobility values of the (a) EDT-bridged PbS QDs assembly and (b) Epitaxially-connected PbS QD-SLs, which were built from different QD diameters, upon application of different gate voltage.

Supplementary Note 5: On Temperature-Dependent Electronic Transport Measurement

A. Variable Range Hopping (VRH)

The analysis of temperature-dependent conductance is performed by considering the variable-range-hopping (VRH) transport mechanism. The conductance as the function of the temperature follows the power law, and it is expressed by Supplementary Equation (6)

$$G(T) = G_0 \exp\left[-\left(\frac{T_0}{T}\right)^{1/d+1}\right] \quad (6)$$

where d = 2 and 3 denote the dimensionality of the Mott VRH for 2D and 3D systems, respectively. On the other hand, d = 1 denotes Efros-Shklovskii (ES) VRH that considers electron-electron interactions. To begin the analysis, we plotted the measured temperature-dependent conductance in the form of logarithmic conductance versus $T^{1/d+1}$ for all possible d values (d = 1, 2, and 3). Supplementary Fig. 24 shows the series of 2D and 3D Mott VRH as well as the ES-VRH plots for two-terminal FET of EDT-bridged PbS QDs assembly (Supplementary Figs. 24a-24c) and the epitaxially-connected PbS QD-SLs (Supplementary Figs. 24e-24g). The temperature-dependent conductance plots fit better towards the 3D Mott VRH (d = 3; $\propto T^{-1/4}$). The R-square of the linear fitting of (ln$G$ vs $T^{-1/4}$) is close to unity for 3D Mott VRH (Supplementary Figs. 24d and 24h). From the VRH analysis, we found that in the epitaxially connected PbS QD SLs, the slope of ln$G$ against $T^{-1/4}$ gets lower as the increase of applied V_G.

B. Insulator-to-Metal Transition (IMT): Mott transition or Anderson transition

To elucidate further intrinsic characteristics and to obtain insights into the charge transport mechanism in the epitaxially-connected PbS QDs, the contact resistance effect of the device should be suppressed. Therefore, further analysis is performed on the four-terminal FET (4T-FET) results. The I_D-V_G transfer characteristics of the 4T-FETs of the epitaxially-connected PbS QD-SLs are shown in Supplementary Fig. 25 and 26 (the characteristic of contact resistance is plotted in Supplementary Fig. 27). The gate-dependent electron mobility values were determined using Supplementary Equation (5)**,** in which the V_{4T} (potential difference of two voltage probes between the drain and source electrodes) is used instead of the applied V_{DS}.

The insulator-to-metal transitions are phenomena in the transport mechanism which can be generally driven by either disorder of the system (weak localisation/Anderson localisation) or the strong correlation that led to the formation of the Coulomb gap (Mott localisation). So far, the transport measurement of the epitaxially-connected PbS QD-SLs indicates that the temperature-dependent conductance may be described as 3D Mott VRH. The plot of sheet resistance (the inverse of sheet conductance) versus $T^{-1/4}$ of the 4T-FET can also be described very well with such an approach (Supplementary Fig. 28a). Further analysis of the existence of disorder-type localisation, which might lead to Anderson localisation (transition), is also considered. The characteristics of disorder-driven localisation (Anderson transition) can be described by the scaling of the sheet resistance against ln$T$, following Supplementary Equation (7) below,

$$R_{sheet}(T) \propto \ln(T) \tag{7}$$

Hence, the perfect match of the characteristics of our epitaxially-connected PbS QD SLs on ln$T$ scaling, shown in Supplementary Fig. 28b, might also indicate the occurrence of weak localisation/Anderson localisation in the vicinity of the transition.

C. Metallic Electron Mobility Estimation at The Vicinity of Insulator-to-Metal Transition

Achieving insulator-to-metal transition in the PbS QD-SLs is an important milestone, in which the number of carrier density surpass a required threshold and the conductance starts surpassing the quantum conductance (G_Q). Low-temperature mobility in the vicinity of insulator-to-metal transition can be estimated from the quantum conductance and the number of the critical carrier density required for the transition to occur (n_c).[12] For epitaxially-connected nanocrystals with diameter d and facial connection radius ρ, the low-temperature metallic mobility (μ_{LT}) can be estimated as:[12]

$$\mu_{LT} = \frac{3^{5/3}}{2\pi^{2/3}} \frac{e}{\hbar} \frac{\rho^2}{g^{2/3} n^{1/3} d} \tag{8}$$

where n is carrier density and g is the number of degeneracy (for PbS system, g is 4)[13]. For epitaxially-connected QD-SL built from PbS QD building block with d = 8.1 nm and average facet connection diameter 2ρ = 4.5 nm, at carrier density $n = n_C \approx 9.8 \times 10^{19}$ cm^{-3}, the estimated

electron mobility is 12.4 cm^2 V^{-1} s^{-1}. At $n = 3.5 \times 10^{20}$ cm^{-3}, the mobility is predicted to be 8.15 cm^2 V^{-1} s^{-1}. This estimated electron mobility values agree with the value obtained from the 4T-FET measurement.

This agreement signifies the importance of evaluating the charge carrier mobility analysis of QD assembly system from transport measurements, including FETs, to check the sensibility of the obtained values.

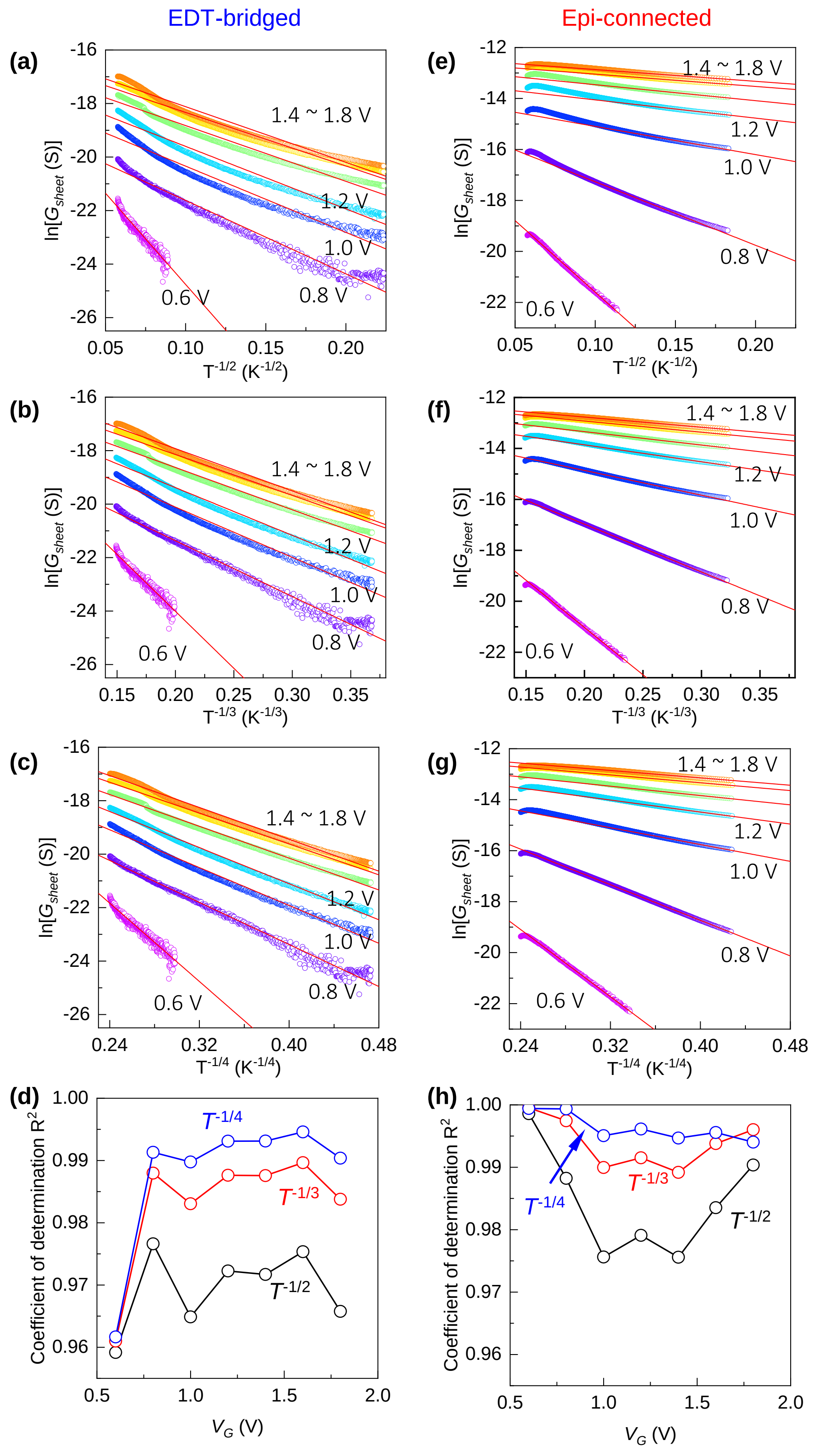

Supplementary Figure 24 | Variable-range hopping (VRH) analyses. (a) Fitting of the temperature-dependent conductance trend of the EDT-bridged PbS QD assembly against the Efros-Shklovskii (ES) VRH with $T^{-1/2}$, (b) 2D Mott VRH with $T^{-1/3}$, and (c) 3D Mott VRH with $T^{-1/4}$. (d) The corresponding R-square parameters of the fittings. (e) Fitting of the temperature-

dependent conductance trend of the epitaxially-connected QD-SL against the Efros-Shklovskii (ES) VRH with $T^{-1/2}$, (f) 2D Mott VRH with $T^{-1/3}$, and (g) 3D Mott VRH with $T^{-1/4}$, as well as (h) the corresponding comparison of the R-square parameters of the fittings.

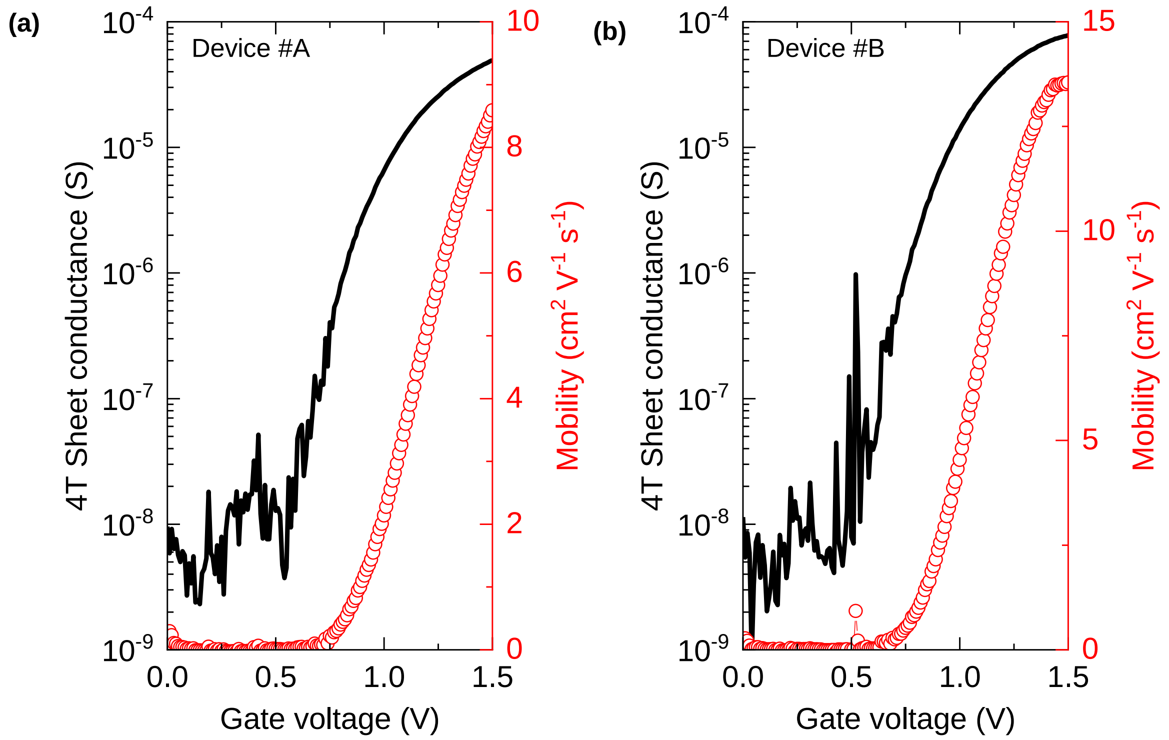

Supplementary Figure 25 | Gate-Modulated Sheet conductance (G_{sheet}-V_G) and electron mobility (μ-V_G) characteristics of the epitaxially-connected PbS QD-SL four-terminal EDLTs obtained from two of the measured devices, labelled as (a) Device A and (b) Device B. The diameter of PbS QD used in those devices is 8.1 nm.

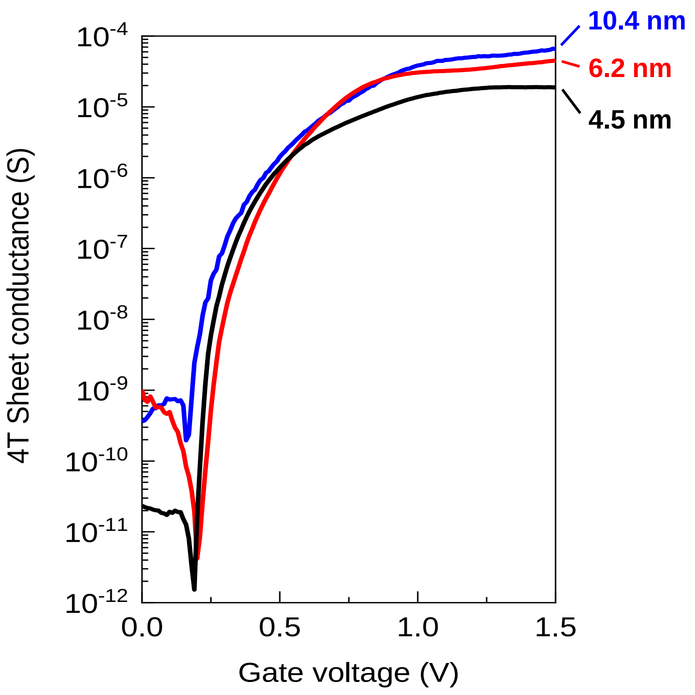

Supplementary Figure 26 | Gate-modulated sheet conductance (G_{sheet}-V_G) characteristics obtained from 4-terminal ionic-liquid-gating FETs of epitaxially-connected PbS QD-SLs built from three different nanocrystal diameters: 4.5 nm (black line), 6.2 nm (red line), and 10.4 nm (blue line).

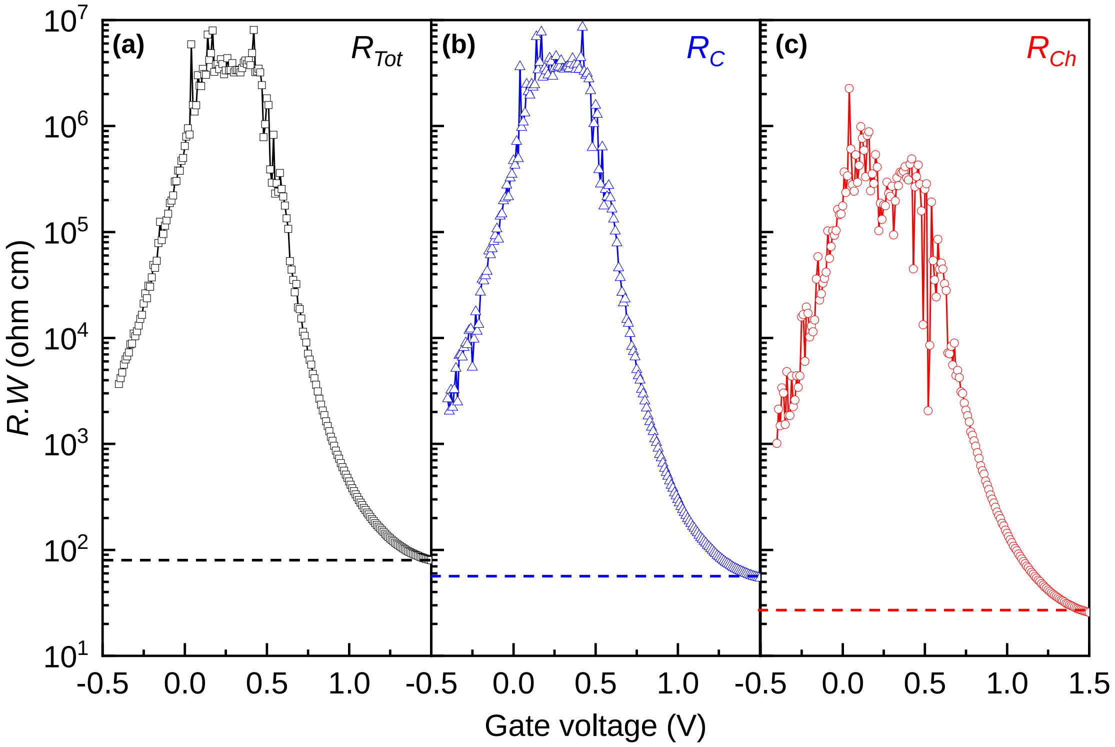

Supplementary Figure 27 | Contact resistance of the four-terminal EDLT. The comparison of the (a) total resistance (R_{Tot}) of the device between the source and drain, (b) the measured contact resistance (R_C), and (c) the measured intrinsic channel resistance (R_{Ch}) of the epitaxially-connected PbS QD-SLs of the channel with channel width *W*. The plots belong to the depicted device B in Supplementary Fig. 25b.

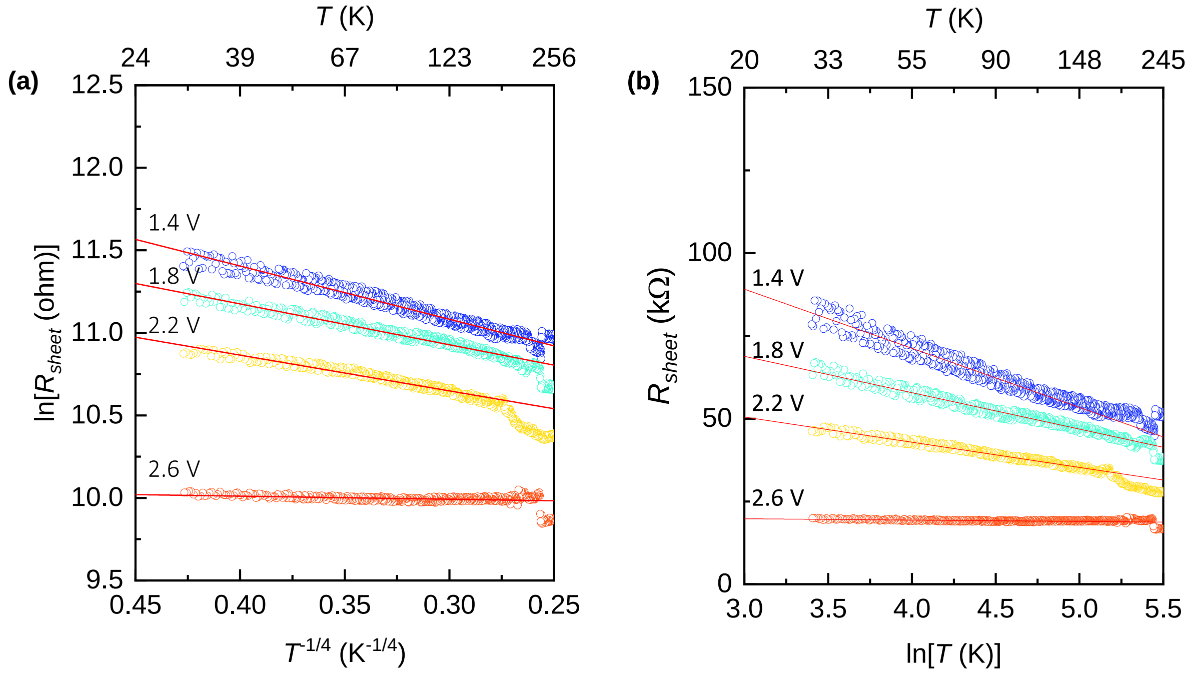

Supplementary Figure 28 | Metal-to-insulator transition in epi-connected PbS QD SLs. (a) Mott-VRH plot of logarithmic sheet resistance (ln[R_{sheet}]) vs. $T^{-1/4}$ and (b) the temperature scaling, ln(T), of the R_{sheet} near the insulator-to-metal transition (1.4 V < V_G < 2.2 V).

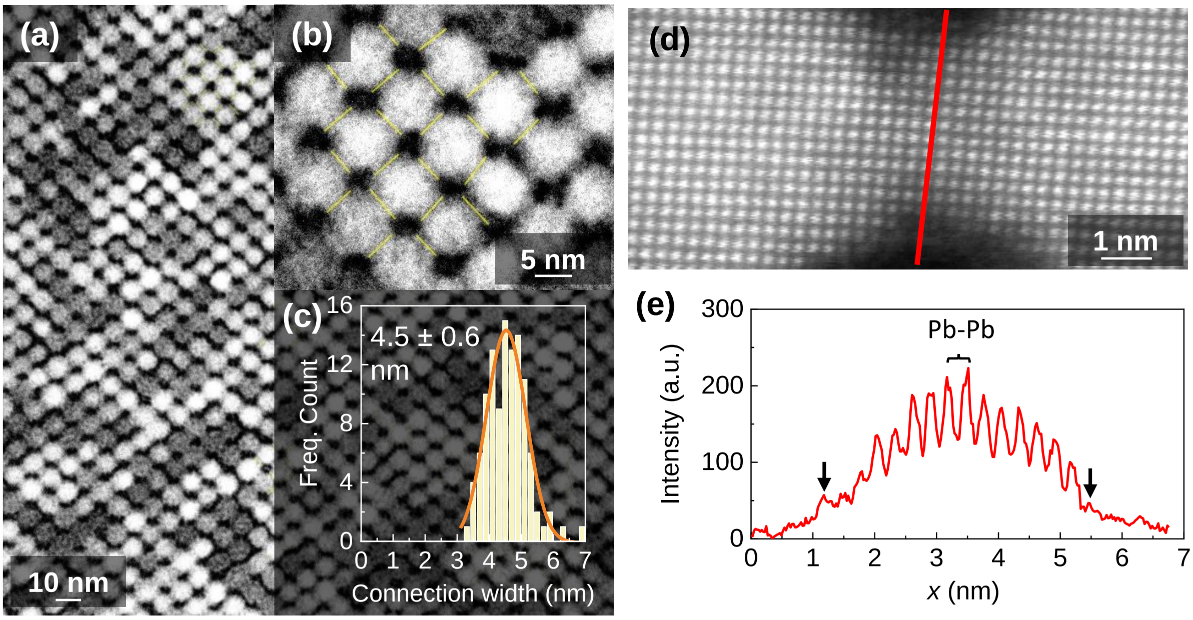

Supplementary Figure 29 | Connection width of the epitaxially-connected PbS QD-SL facet. (a) Invert of a TEM image of the epitaxially-connected PbS QD-SL with QD diameter of 8.1 nm. (b) The corresponding magnified image with pointers of the epitaxial connection cross-sections. (c) The width distribution of the epitaxial connection. (d) The high resolution of the dark field STEM (obtained using Talos-F200X TEM) is also used to resolve the (e) epitaxial connection width more precisely. Two arrows denote the edges of the connection. Both measurements show that the diameter of the epitaxial connection cross-section is approximately 4.5 nm.

Supplementary References

1. Nugraha, M. I. *et al.* Strain-Modulated Charge Transport in Flexible PbS Nanocrystal Field-Effect Transistors. *Adv. Electron. Mater.* **3**, 1600360 (2017).
2. Septianto, R. D. *et al.* On-demand tuning of charge accumulation and carrier mobility in quantum dot solids for electron transport and energy storage devices. *NPG Asia Mater.* **12**, 33 (2020).
3. Balazs, D. M., Dunbar, T. A., Smilgies, D. M. & Hanrath, T. Coupled Dynamics of Colloidal Nanoparticle Spreading and Self-Assembly at a Fluid-Fluid Interface. *Langmuir* **36**, 6106–6115 (2020).
4. Smilgies, D. M., Heitsch, A. T. & Korgel, B. A. Stacking of Hexagonal Nanocrystal Layers during Langmuir–Blodgett Deposition. *J. Phys. Chem. B* **116**, 6017–6026 (2012).
5. Jiang, Z. GIXSGUI : a MATLAB toolbox for grazing-incidence X-ray scattering data visualization and reduction, and indexing of buried three-dimensional periodic nanostructured films. *J. Appl. Crystallogr.* **48**, 917–926 (2015).
6. Weidman, M. C., Smilgies, D.-M. & Tisdale, W. A. Kinetics of the self-assembly of nanocrystal superlattices measured by real-time in situ X-ray scattering. *Nat. Mater.* **15**, 775–781 (2016).
7. Sze, S. M. & Ng, K. K. *Physics of Semiconductor Devices*. (John Wiley & Sons, 2007).
8. Bisri, S. Z., Shimizu, S., Nakano, M. & Iwasa, Y. Endeavor of Iontronics: From Fundamentals to Applications of Ion-Controlled Electronics. *Adv. Mater.* **29**, 1607054 (2017).
9. Gutiérrez-Lezama, I., Ubrig, N., Ponomarev, E. & Morpurgo, A. F. Ionic gate spectroscopy of 2D semiconductors. *Nat. Rev. Phys.* **3**, 508–519 (2021).
10. Tsuno, M. *et al.* Physically-based threshold voltage determination for MOSFET's of all gate lengths. *IEEE Trans. Electron Devices* **46**, 1429–1434 (1999).
11. Yuan, H. *et al.* Electrostatic and Electrochemical Nature of Liquid-Gated Electric-Double-Layer Transistors Based on Oxide Semiconductors. *J. Am. Chem. Soc.* **132**, 18402–18407 (2010).
12. Chen, T. *et al.* Metal-insulator transition in films of doped semiconductor nanocrystals. *Nat. Mater.* **15**, 299–303 (2016).
13. Guyot-Sionnest, P. Electrical Transport in Colloidal Quantum Dot Films. *J. Phys. Chem. Lett.* **3**, 1169–1175 (2012).
