# Supplementary material for: Enabling metallic behaviour in two-dimensional superlattice of semiconductor colloidal quantum dots
Source: Nat Commun. 2023 May 26;14:2670. doi: 10.1038/s41467-023-38216-y (PMC10220219; doi:10.1038/s41467-023-38216-y)
Supplement: Supplementary file 2 — Peer Review File [file 41467_2023_38216_MOESM2_ESM.pdf]

nature portfolio

Peer Review File

Enabling Metallic Behaviour in Two-Dimensional Superlattice of Semiconductor Colloidal Quantum Dots

Open Access This file is licensed under a Creative Commons Attribution 4.0 International License, which permits use, sharing, adaptation, distribution and reproduction in any medium or format, as long as you give appropriate credit to the original author(s) and the source, provide a link to the Creative Commons license, and indicate if changes were made. In the cases where the authors are anonymous, such as is the case for the reports of anonymous peer reviewers, author attribution should be to 'Anonymous Referee' followed by a clear attribution to the source work. The images or other third party material in this file are included in the article's Creative Commons license, unless indicated otherwise in a credit line to the material. If material is not included in the article's Creative Commons license and your intended use is not permitted by statutory regulation or exceeds the permitted use, you will need to obtain permission directly from the copyright holder. To view a copy of this license, visit http://creativecommons.org/licenses/by/4.0/.

REVIEWER COMMENTS

Reviewer #1 (Remarks to the Author):

This paper studies highly-ordered 2-dimensional arrays of colloidal PbS quantum dots with epitaxial connections. Getting such a system was not easy and authors overcame many barriers. The use of room temperature ion liquid allowed authors to continuously tune electron concentration by the gate voltage, study the insulator-metal transition and reach high mobility over 10 cm2. In insulating phase they see variable range conductivity and claim that it can be described by 3D Mott variable range hopping law with power 1/4. This an interesting and important paper and I suggest to publish it in Nature Communications after authors respond to my comment below.

I wonder why the authors talk about 3D variable range Mott conductivity with power 1/4, while their system is two-dimensional? For 2D Mott theory gives power 1/3 and Efros-Shklovskii theory gives power 1/2. In Fig. 4c of this paper for 4 -terminal samples there is no convincing data which allows to discriminate between 1/3 and 1/2. To get such data authors should go to lower temperatures T < 20K as this was done in 3D epitaxial nanocrystal arrays where the power 1/2 predicted by Efros- Shklovskii theory in 3D was observed in the very large dynamic range of resistances (see Ref. 10 and B. L. Greenberg, et all Nano Lett. 2017, 17, 4634–4642). This 3D observation makes likely that in the authors 2D samples Efros-Shklovskii power 1/2 works as well. Efros-Shklovskii power 1/2 observed in many 2D system including insulating phases with localized by disorder electrons in Quantum Hall Effect. References to such data can be found in Physical Review B48, 11167 (1993).

Reviewer #2 (Remarks to the Author):

This paper is a careful work on the superlattices of PbS quantum dots, epitaxially connected. The authors do an excellent structural characterization, and also an excellent electrical characterization. This is not easy, and the proper expertise is brought to the topic. I particularly appreciated that the authors did a careful job, measuring the actual gate voltage dependent capacitance of the film rather than assuming a constant value.

Overall, I recommend publication as is, although I have numerous comments below.

Comments:

1) In my opinion, the most interesting result is that the mobility is independent of particle size. To me, this strongly indicates that the transport is coherent across particles. Although not surprising that it should be, I do not think that this result has been reported by others before. The authors also used 4 probe measurements to counter the effect of film inhomogeneity and this allows them to extract larger

values of the mobility of ~ 10cm2/Vs which is quite decent for PbS SL system, while not exceptional for quantum dots. It would be nice to verify that the constant mobility with size is retained in the 4 probe setup (it seems only the 8.1nm was measured in that setup).

2) The discussion of Mott vs ES hopping at low doping level is not very strongly supported by the data, because the fits are over a small range and all about equally precise. This should be acknowledged so that others do not think that this is an ok standard of proof.

3) The metal insulator transition discussion is based on the prior work by Kortshagen and Shklovskii ref. 59 which explored similar MIT with highly doped Si nanocrystal films and later ZnO nanocrystal films. Order is not required at all and the main theoretical basis for the transition is that the interparticle conductance reaches the quantum conductance. Having many channels, and many electrons, is the key. This is how the doping threshold is derived. From the quantum conductance and the number of carriers, the author can extract the mobility, as shown in ref. 59, and it would be interesting to see if this is similar to the experimental mobility.

4) The same idea was quantitatively used before to explain why metal nanoparticle films can be readily metallic, in contrast to quantum dots. The challenge of reaching the MIT is when the density of state is small. As already pointed in "Electrical transport in colloidal quantum dot films" 2013, which is not being referenced, it is increasingly difficult to reach the quantum conductance with a small number of channels (e.g. for state-resolved transport). In the conclusion, the authors state that "the minimization of disorders in the attached but confined system, promotes charge carrier delocalization". I would argue that minimizing disorder is not as critical as they claim, and some evidence of the contrary would be good. Can the author add a discussion whether order is required.

5) It would be useful to give the number of electrons per dot at the MIT. With their reported charge density and dot sizes, and for one monolayer of SL, I get about 15 electrons/dots to reach the MIT for 6 nm dots. This is a filled 1S band and more. Yet, the authors do not see a modulation of the conductance indicating a filled band. Can they discuss why?

6) Upon the very reducing gate potential, can the authors confirm that the system can still be reversibly brought back to a lower gate potential, and confirm that the effect is not polluted by the formation of metallic Pb?

7) Line 215: "the observed size- 10 216 dependent bandgap values probed by the EDLTs (Fig. S17) and excitonic peak in their 217 absorption spectra confirmed that quantum confinement nature is preserved (Fig. S18" I would comment that, while it is true that quantum confinement is preserved, the authors are not confirming 3D confinement (but 2D is left). Second, I am 100% certain that the "bandgap values" cannot be obtained from EDLT in the way described in the SI. This refers to Fig S17: This figure purports to show that the gap of the quantum dots can be extracted by simply taking a linear line going to zero, and match its slope approximately to the slope of the transport curve. Prior work showed that this is totally misleading because, on a log scale, there is no break at all. The same data should indeed be plotted on a log scale, and the authors will see that both sides of the transconductance vs reference are simple Arrhenius lines with a 1/kT slope, and that there is no "threshold" and no information retrievable about the gap. In fact, the only way to get an information on the gap is to actually extract the density of states, and this should give a bigger gap including the Coulomb charging energy. This error was previously made by the last author.

Reviewer #3 (Remarks to the Author):

The manuscript by Bisri and coauthors reports the observation of metallic transport from epitaxially connected PbS quantum dot (QD) assemblies. QDs with intriguing quantum mechanical characteristics have been exploited for optoelectronic device applications (especially the light-emission or light absorption devices). Although significant progress has been made in field (even commercial products based on QDs are available), the electrical transport characteristics of QDs, through the assemblies of the materials, remain interior, which is known to be limited by inefficient hopping transport steps of charge carriers between the physically separated semiconductor crystals. This may serve as one of critical bottlenecks hampering the enhancement of device performances. The authors claim that by forming epitaxially connected superlattice of QDs (that can minimize orientational disorder among the QDs and increase the electronic overlapping between the neighboring localized states) and gating the superlattice with electrolyte (that can induce extremely high density of charge carriers to fill the sub-states impeding transport), metallic transport behavior is observed at high carrier density condition. This is a very important achievement in the field, and the manuscript is well-written. However, due to the following concerns, I cannot suggest its publication to Nature Communications at this point. If they are addressed carefully, I believe the results have good potential for publication in Nature Communications.

1. Typically, metallic transport (through delocalized electronic states limited by scattering) is signatured with negative conductivity vs. temperature correlation, whereas insulator transport (through series of localized states assisted by thermal energy) follows positive conductivity vs. temperature correlation. The authors clearly showed that the highly negative conductivity vs. temperature correlation at low carrier density conditions (small VGs) gradually approaches to a smaller value (close to zero) by inducing high carrier density (larger VGs) to the epitaxially-connected PbS QD superlattices. However, I am not fully convinced if the metallic transport is attained as the conductivity at the highest carrier density condition follows rather temperature-independent characteristics (or zero conductivity vs. temperature correlation), and explanation is needed.

2. Also, metallic transport, the carrier mobility (different from conductivity) should be hampered by increasing charge carrier density (at low charge density conditions where insulator-type transport occurs through disordered states, increasing the charge carrier density helps filling the substates and thus can facilitate transport). The carrier density dependence of charge carrier mobility should also be confirmed.

3. I believe this part is the core finding of the manuscript and additional experiment to support the formation of the extended, delocalized state for transport at high carrier density conditions should be

given. Here is my suggestion. Epitaxially-connected PbS QD superlattices exhibits insulator-type transport (thermally activated transport with positive conductivity vs. temperature correlation) at the low carrier density conditions, which are in their “attached-but-confined” (therefore localized) electronic state. At these conditions, the confinement of the electronic states was confirmed using UV-Vis-NIR absorbance measurement showing characteristic excitonic absorbance feature (Figure S18), which is good. At high carrier density conditions, the same superlattices now gated with electrolyte follow metallic transport indicating that the formation of delocalized state. This would now be “attached-and-delocalized” state - if I tweak the term the authors used. This should lead to apparent change in the electronic states of the superlattice, and the change should be observable through spectroelectrochemical measurement (monitor the UV-Vis-NIR spectrum while applying potentials to the ionic liquid gated devices). See Science 2003 (10.1126/science.1084424). The in-situ measurement should be readily done from the combinations of the setup the authors already have (spectroscopy and electrical input/readouts).

4. Another concern is about the influence of QD size on transport. It was found that the size of QDs constructing the epitaxially-connected superlattice does not affect the transport characteristics. This is different from previous results (as already stated in the manuscript). The author should explain the why the difference occurs for the epitaxially connected PbS QDs (compared to EDT-bridged ones).

5. In fact, the size-independent mobility values shown in Figure 3d is confusing. Clearly, the current levels of the transfer characteristics shown in Figure 3b and the output characteristics in Figure S13 are all different or size-dependent. How would the mobility values extracted from these size-dependent raw data be size-independent. This seems counterintuitive.

6. Moreover, the authors have already confirmed that the contact resistance is a critical problem in the devices they tested. (Compare Figure 4b and 4c). If this is the case, the mobility value should be extracted with 4-probe measurement (instead of 2-probe measurement done in Figure 3b) to exclude any influence of the contacts in estimating mobility.

7. If the mobility and thereby the associated transport characteristics are size-independent, the temperature-dependence of the mobility for epitaxially-connected PbS QD superlattice should be also confirmed. They should be size-independent too. This needs to be definitely confirmed.

8. The description determining the hopping model from in Figure S21 is hardly convincing. The raw temperature dependent conductivity data can be fitted to different models following different temperature dependence. The authors determined that the transport follows the 3D Mott VRH model, simply based on the R^2 values of the fit. Even if the R^2 values for the fitting based on the 3D Mott VRH model shows the largest value among the series (Figure S21 d and h), all these values are in the range between 0.96 and 1.00 (the 2D VRH model fitting yields R^2 values between 0.99 and 1.00).

Under this situation, I am not fully convinced if one can determine the transport model from the method based on R^2 values.

9. All the study in the manuscript was done by analyzing the electron transport. PbS QDs follow ambipolar transport. Is there any reason why the study was done for electron transport only? Would there be different findings if the study was done based on hole transport?

10. If the superlattice is made of monolayer thick PbS QDs which with diameter of 4-10 nm, it means that the 4-10 nm film is the channel which is placed on top of 40 nm thick electrode (Au 30 nm and Ti 10 nm) in the given bottom contact device structure. In this case, is the electrode really the Au or is it the Ti? Also weren't there any issues with leakage current? (The gate current should be also plotted along with the transfer characteristics to clarify this concern).

11. In page 6 and 7, the authors stated that the superlattice with uniform preferential orientation is on a large scale of 20 micrometers. Can the authors show SEM images of the 20 micrometer superlattice?

12. Why was the 4-probe measurement done using side-gate EDLT? Is there a chance that the charge accumulation in the superlattice be different when using side-gate compared to using conventional top-gate?

13. The capacitance value will be given from the equipment and this value would be divided by the area. I am curious how the authors determined the area for obtaining specific capacitance values.

Point-by-point responses to reviewers
NCOMMS-22-37189A
"Enabling Metallic Behaviour in Two-Dimensional Superlattice of Semiconductor Colloidal Quantum Dots"

Response to Reviewer 1:

COMMENT #1:
This paper studies highly-ordered 2-dimensional arrays of colloidal PbS quantum dots with epitaxial connections. Getting such a system was not easy, and the authors overcame many barriers. The use of room temperature ion liquid allowed authors to continuously tune electron concentration by the gate voltage, study the insulator-metal transition and reach high mobility over 10 cm2. In insulating phase they see variable range conductivity and claim that it can be described by 3D Mott variable range hopping law with power 1/4. This an interesting and important paper and I suggest to publish it in Nature Communications after authors respond to my comment below.

Answer:

We are glad to receive the reviewer`s appreciation regarding the challenges of carrying out this work. Indeed, we found out that by carefully preparing the epitaxially-connected QD-SLs, we can establish a way to investigate the charge carrier transport reliably. Therefore, we are pleased by the reviewer's comments on the importance of our work. We would like to respond reviewer`s further comments as follows.

COMMENT#2:
I wonder why the authors talk about 3D variable range Mott conductivity with power 1/4, while their system is two-dimensional? For 2D Mott theory gives power 1/3 and Efros-Shklovskii theory gives power 1/2.

Answer:

We understand the reviewer`s concern regarding our discussion on the 3D variable range Mott conductivity with a power of 1/4. Indeed, the samples that we worked on are the

monolayer QD assemblies, which resemble two-dimensional systems. However, we also realised that the actual thickness of the monolayer depends on the diameter of the QD building blocks that we used, which range from 4.5 nm to 10.4 nm, while the temperature-dependent measurement was performed on the samples with the QD diameter of 8.1 nm.

Before we concluded that we discussed more 3D Mott-VRH, we compared all models 3D and 2D Mott-VRH with power 1/4 and 1/3, respectively, and ES-VRH with power 1/2, shown in the supplementary information, Supplementary Notes 5a and Supplementary Fig. 24. By comparing all the models side-by-side, in terms of linear fitting results, the power 1/4 gives us the highest coefficient of determination (R^2) value which means that the data arranged in $(1/T)^{1/4}$ fit well to the equation. Although the power 1/3 and 1/2 show the R^2 of more than 0.96, they are adequate to be chosen, especially on the epitaxially-connected structure. Therefore, to make a more suitable explanation regarding the mechanism, we clarify our statement in the main text about the VRH. It can be found in the Discussion section, paragraph 2 (pages 13-14), as shown below.

The original paragraph,

"The controllability of the assembly formation and the fine-tuning of the carrier density by the ionic gating enable us to discuss the charge carrier transport in the QD assemblies. The high carrier density accumulation in the compact well-ordered monolayer of EDT-bridged QD assemblies shows that the transport mechanism strongly deviates from the nearest-neighbour hopping (NNH) Arrhenius-type behaviour. We then compared possible scenarios of different types of variable range hopping (VRH). Within the given temperature range, the transport process fits best with Mott 3D-VRH (Supplementary Fig. 24c) while largely deviating from Efros-Skhlovskii (ES) VRH ("$T^{-1/2}$). Due to the high carrier density accumulation by the ionic gating, there is no observable transition from NNH.[30] The observation of Mott 3D-VRH ("$T^{-1/4}$) indicates that significant disorder remains due to the non-coherence of the QD orientation in the EDT-bridged PbS QDs. However, they can achieve highly ordered assembly with uniform inter-QD distance."

was revised to become

"The controllability of the assembly formation and the fine-tuning of the carrier density by the ionic gating enable us to discuss the charge carrier transport in the QD assemblies. The high carrier density accumulation in the compact and well-ordered monolayer of EDT-bridged QD assemblies shows that the transport mechanism strongly deviates from the nearest-neighbour hopping (NNH) Arrhenius-type behaviour, which is transforming to the variable range hopping (VRH). We then compared possible scenarios of different types of VRH.

Within the given temperature range, the transport process fits best with Mott 3D-VRH (Supplementary Fig. 24c). However, we found that the fit also works well with Efros-Skhlovskii (ES) and Mott 2D-VRH and (Supplementary Figs. 24a and 24b, respectively). Rigorous identification of the hopping mechanisms should be made at lower temperatures, e.g., $T < 20$K,[59] which was not covered in the current study and thus is left for a future study. The steeper temperature dependence and the Mott-VRH-like behaviour indicate that significant disorder remains due to the non-coherence of the QD orientation in the EDT-bridged PbS QDs, despite the uniform inter-QD distance."

COMMENT#3:

In Fig. 4c of this paper for 4 -terminal samples there is no convincing data which allows to discriminate between 1/3 and 1/2. To get such data authors should go to lower temperatures T < 20K as this was done in 3D epitaxial nanocrystal arrays where the power 1/2 predicted by Efros- Shklovskii theory in 3D was observed in the very large dynamic range of resistances (see Ref. 10 and B. L. Greenberg, et all Nano Lett. 2017, 17, 4634–4642). This 3D observation makes likely that in the authors 2D samples Efros-Shklovskii power 1/2 works as well. Efros-Shklovskii power 1/2 observed in many 2D system including insulating phases with localised by disorder electrons in Quantum Hall Effect. References to such data can be found in Physical Review B48, 11167 (1993).

Answer:

As the reviewer pointed out, we indeed realise that at this moment, we still have difficulties in discriminating the power factor of the VRH analyses for being 1/4, 1/3 and 1/2 from the current low-temperature measurement. Hence, to address this situation, we revised the discussion about transport mechanisms in the main text, as shown in answer to the previous comment.

Response to Reviewer 2:

COMMENT #1:

This paper is a careful work on the superlattices of PbS quantum dots, epitaxially connected. The authors do an excellent structural characterisation, and also an excellent electrical characterisation. This is not easy, and the proper expertise is brought to the topic. I particularly appreciated that the authors did a careful job, measuring the actual gate voltage dependent capacitance of the film rather than assuming a constant value.

Answer:

We thank the reviewer for appreciating our work and for understanding the challenges.

COMMENT #2:

Overall, I recommend publication as is, although I have numerous comments below.

Answer:

We are glad to read the reviewer`s recommendation. Furthermore, the reviewer also provided comments that will become essential references for further studies in this research field. We would like to address the reviewer`s comments carefully based on our current understanding. Nevertheless, we directly follow some suggestions that can be performed immediately to improve our current manuscript.

COMMENT #3:

In my opinion, the most interesting result is that the mobility is independent of particle size. To me, this strongly indicates that the transport is coherent across particles. Although not surprising that it should be, I do not think that this result has been reported by others before. The authors also used 4 probe measurements to counter the effect of film inhomogeneity, and this allows them to extract larger values of the mobility of ~10 cm2/Vs which is quite decent for PbS SL system, while not exceptional for quantum dots. It would be nice to verify that the constant mobility with size is retained in the 4-probe setup (it seems only the 8.1nm was measured in that setup).

Answer:

We appreciate the reviewer`s comment and suggestion. Indeed we initially focused on working only with a 2-terminal FET configuration. It was motivated by the present condition

that the 2-terminal configuration is still the primary benchmark for analysing the charge carrier transport of the electronic devices in the colloidal QDs community. Most of the existing works utilising FETs (either a solid gate or electrolyte gate) to report the carrier mobility values of holes, electrons, or both were of a 2-terminal configuration. By utilising 2-terminal FET devices, we could directly compare our results with the existing literature.

We realised that to understand better the charge carrier transport properties of the QD assemblies, performing 4-terminal FET measurements would be crucial. By removing the influence of contact resistance, we would obtain the fundamental intrinsic properties important for our purpose. Therefore, we also performed the 4-terminal measurements.

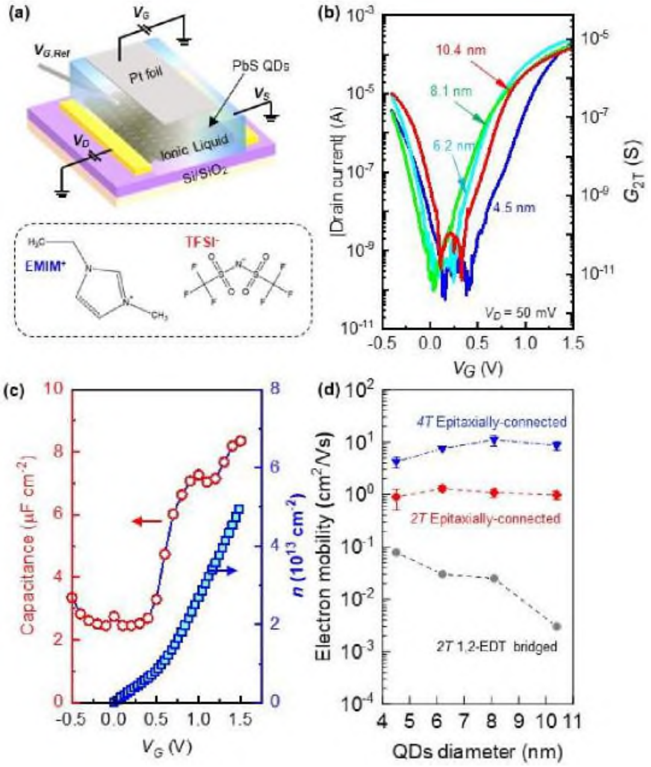

Figure 3 | Size-Independent Mobility in Electric-Double-Layer Transistors (EDLT) of PbS epitaxially-connected QD-SLs. (a) A schematic of the two-terminal EDLT device structure of the PbS QD-SL using EMIM-TFSI ionic liquid as electrolyte gate. **(b)** I_D-V_G transfer characteristics of the QD-SLs prepared from PbS QD with different diameters show ambipolar transport with a well-defined intrinsic region and a high on/off ratio for holes and electrons. **(c)** The gate-voltage-dependent capacitance value of the EDLT (red circle) and the corresponding accumulated sheet electron density (blue square) were extracted from potentiostatic electrochemical-impedance-spectroscopy (EIS) measurements. **(d)** Plots of the characteristic electron mobility values of the epitaxially-connected QD-SLs EDLT versus the diameter of the QDs (red diamond) showing size-independent values, in contrast to the conventional (decreasing) size-dependent electron mobility in the ligand-capped QD assemblies (i.e. EDT-capped PbS QD, black circle), the 4-terminal FET mobility of epitaxially-connected PbS QD-SLs (blue triangle) show the similar behaviour to the corresponding 2-terminal with higher mobility value.

Following the reviewer`s suggestion, in the revised manuscript, we also presented the QD-diameter-dependent electron mobility values of the QDSLs that were probed using 4-

terminal FET measurements. Both the 4-terminal FET and the 2-terminal FET measurements are consistent, and we still observed the constant mobility values. Obviously, the values extracted from the 4-terminal FET measurements are larger than those obtained from 2-terminal FET measurements since the influence of the contact resistance has been removed.

In the manuscript, we modified Fig. 3d also to contain the 4-terminal FET mobility. We also added the 4-terminal sheet conductance to Supplementary Fig. 26.

COMMENT #4:

The discussion of Mott vs ES hopping at low doping level is not very strongly supported by the data, because the fits are over a small range and all about equally precise. This should be acknowledged so that others do not think that this is an ok standard of proof.

Answer:

We agree with the reviewer that the discussion of Mott vs ES hopping at a low doping level is still limited, stemming from the relatively narrow temperature (1/T) range. There were still two challenges that we still encountered. The first is that at a very low doping level (low applied gate voltage $V_G < 0.8$ V) and low temperature, the conductance values have already become very low and are starting to compete with the noise level of our setup. One possible way is to enlarge our device channel size, which will create another problem. The second challenge is related to the limitation of our cryostat system, which is connected to the glovebox to maintain device stability. At this point, it cannot perform measurements below 30K reliably.

Nevertheless, we agree that to make a more comprehensive discussion about the transport mechanism at a low doping level, Mott vs ES-VRH comparison, performing measurements at a much lower temperature is essential [e.g. *ACS Nano* 4, 5211-5216 (2010)]. It will be a subject for our next endeavour, either by improving the performance of our glovebox-connected cryostat; or by stabilising our devices so that we can transfer them safely to the other cryostats that can perform much lower temperature measurements without any alteration of their electronic properties due to oxygen or moisture exposure. Therefore, to make a more suitable explanation regarding the mechanism, we clarify our statement in the main text about the VRH. It can be found in the Discussion section, paragraph 2 (pages 13-14).

The original paragraph,

"The controllability of the assembly formation and the fine-tuning of the carrier density by the ionic gating enable us to discuss the charge carrier transport in the QD assemblies. The high carrier density accumulation in the compact well-ordered monolayer of

EDT-bridged QD assemblies shows that the transport mechanism strongly deviates from the nearest-neighbour hopping (NNH) Arrhenius-type behaviour. We then compared possible scenarios of different types of variable range hopping (VRH). Within the given temperature range, the transport process fits best with Mott 3D-VRH (Supplementary Fig. 24c) while largely deviating from Efros-Skhlovskii (ES) VRH ("$T^{-1/2}$). Due to the high carrier density accumulation by the ionic gating, there is no observable transition from NNH.[30] The observation of Mott 3D-VRH ("$T^{-1/4}$) indicates that significant disorder remains due to the non-coherence of the QD orientation in the EDT-bridged PbS QDs. However, they can achieve highly ordered assembly with uniform inter-QD distance."

was modified to become

"The controllability of the assembly formation and the fine-tuning of the carrier density by the ionic gating enable us to discuss the charge carrier transport in the QD assemblies. The high carrier density accumulation in the compact and well-ordered monolayer of EDT-bridged QD assemblies shows that the transport mechanism strongly deviates from the nearest-neighbour hopping (NNH) Arrhenius-type behaviour, which is transforming to the variable range hopping (VRH). We then compared possible scenarios of different types of VRH. Within the given temperature range, the transport process fits best with Mott 3D-VRH (Supplementary Fig. 24c). However, we found that the fit also works well with Efros-Skhlovskii (ES) and Mott 2D-VRH and (Supplementary Figs. 24a and 24b, respectively). Rigorous identification of the hopping mechanisms should be made at lower temperatures, e.g., $T <$ 20K,[59] which was not covered in the current study and thus is left for a future study. The steeper temperature dependence and the Mott-VRH-like behaviour indicate that significant disorder remains due to the non-coherence of the QD orientation in the EDT-bridged PbS QDs, despite the uniform inter-QD distance."

COMMENT #5:

The metal insulator transition discussion is based on the prior work by Kortshagen and Shklovskii ref. 59 which explored similar MIT with highly doped Si nanocrystal films and later ZnO nanocrystal films. Order is not required at all and the main theoretical basis for the transition is that the interparticle conductance reaches the quantum conductance. Having many channels, and many electrons, is the key. This is how the doping threshold is derived. From the quantum conductance and the number of carriers, the author can extract the mobility, as shown in ref. 59, and it would be interesting to see if this is similar to the experimental mobility.

Answer:

The reviewer raised an interesting proposition. Indeed, we discuss the possible origin of MIT in the epitaxially-connected PbS QDs assembly based on the previous work of Shklovskii and Kortshagen. As explained in the manuscript, by utilising the established theoretical framework, we can estimate the critical carrier concentration for the epitaxially-connected QDs. For the epitaxially-connected QDSLs that utilise 8.1 nm diameter QD, the estimated critical carrier density value, $n_{C,}$ is around 9.8×10^{19} cm^{-3} (or equivalent to 2.1×10^{13} cm^{-2}), which can be achieved by applying gate voltage for more than 1.1 V. At 1.5 V of V_G, we obtained the carrier density about 5×10^{13} cm^{-2} or equivalent to 3.5×10^{20} cm^{-3} (it can be varying from sample to sample).

Nevertheless, we are interested in the reviewer's suggestion to predict the charge carrier mobility from the quantum conductance and the number of carriers. The formula in ref. 59 mentioned that the mobility of epitaxially-connected QDs in the metallic state could be defined as:

$$= \frac{3\,5\ /3}{2\,2\ \ /3} \ \frac{2}{\hbar\ \ 2\,/\,3\ 1\,/\,3}$$

At carrier density $n = n_C \approx 9.8 \times 10^{19}$ cm^{-3}, the estimated electron mobility is 12.4 cm$_2$ V^{-1} s^{-1}. At $n = 3.5 \times 10^{20}$ cm^{-3}, the mobility is predicted to be 8.15 cm$_2$ V^{-1} s^{-1}. To some extent, this estimated electron mobility value agrees with the value obtained from the 4-terminal FET measurement.

We believe such control proposed by the reviewer is also necessary to check the sensibility of any reported carrier mobility values in the literature and in many future studies. Therefore, we added hint regarding this comparison in page 16 and a corresponding discussion in the Supplementary Note 5C highlighting its importance.

COMMENT #6:

The same idea was quantitatively used before to explain why metal nanoparticle films can be readily metallic, in contrast to quantum dots. The challenge of reaching the MIT is when the density of state is small. As already pointed in "Electrical transport in colloidal quantum dot films" 2013, which is not being referenced, it is increasingly difficult to reach the quantum conductance with a small number of channels (e.g. for state-resolved transport). In the conclusion, the authors state that "the minimisation of disorders in the attached but confined system, promotes charge carrier delocalisation". I would argue that minimising disorder is not as critical as they claim, and some evidence of the contrary would be good. Can the author add a discussion whether order is required.

Answer:

Thank you for the reviewer's comment. It is true that if we stick to the theoretical perspective derived by Shklovskii and team, the MIT in semiconductor nanocrystals can be achieved when we can provide more channels (by making connections) and sufficiently high carrier density. However, according to this point of view, the facet connection is crucial for achieving the quantum conductance (or metallic state). Quantum conductance can be realised if we have a well-defined facet connection. For instance, we can compare two connected QDs systems, without and with an order, as illustrated in Fig. R1 below. The ordered system (Fig. R1b) provides more homogenous and well-defined facet connections, by which the critical carrier density can be estimated to achieve metallicity in QDs assembly. In contrast, it will not be the case for the "random" assembly of QDs. The comparison of electronic properties in ordered and disordered assemblies of QDs has been shown in our previous work [*Nanoscale* 11, 20467-20474 (2019)]. Over-stripping the ligands from the QDs during the assembly process would destroy the assembly orders but the QDs can make contacts randomly. In this condition, the conductance values of the assembly are much lower than that of the well-ordered square-lattice assembly, even if they were still not well-connected.

Another essential point to be noted is the unique condition of lead-chalcogenide QDs (e.g. PbS and PbSe). In the assemblies of these QD compounds, both the superlattice orders and the atomic lattice are coupled, which would immediately happen when the assemblies are epitaxially connected. On the other hand, the assemblies without coherent atomic lattice order are typically obtained if there are spaces among the QDs, usually created by the existence of ligand capping or ligand bridge surrounding the nanocrystals, e.g. EDT-bridged PbS QDs assembly. We cannot controllably obtain the superlattice condition where the QDs are epitaxially connected but without atomic ordering in the lead-chalcogenide system.

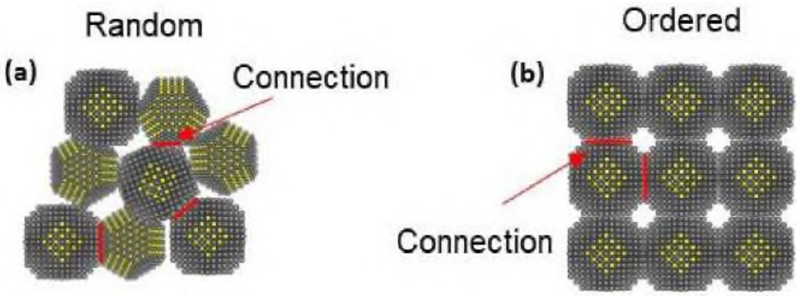

Figure R1 | Illustration of QDs assemblies. (a) The connected QDs with disorder and un-oriented assembly, (b) the connected QD with highly ordered and atomically-oriented, showing well define connection facet.

COMMENT #7:

It would be useful to give the number of electrons per dot at the MIT. With their reported charge density and dot sizes, and for one monolayer of SL, I get about 15 electrons/dots to

reach the MIT for 6 nm dots. This is a filled 1S band and more. Yet, the authors do not see a modulation of the conductance indicating a filled band. Can they discuss why?

Answer:

We thank the reviewer for suggesting giving the number of electrons per dot at the insulator-to-metal transition. The temperature-dependent measurement was performed using PbS QDs with a diameter of 8.1 nm. Considering the size of QDs and the assembly structure of epitaxially-connected, we obtained the gate-dependent number of electrons per dot as shown in Fig. R2 below. Considering the 4-fold degeneracy of the PbS QD, at V_G = 1.5 V or more, will provide us with more than 30 electrons/QDs sufficient to fill the energy state of $1S_e$ and $1P_e$.

Although we obtained a considerable amount of electron density using ionic-gating FET, we could not clearly observe the signature of band-filling in the transport measurement, similar to what had been demonstrated in mercury chalcogenides [*Nature Mater.* 19, 323-329 (2020); *J. Phys. Chem. Lett.* 11, 2303–2307 (2020)] and cadmium chalcogenides [*Science* 300, 1277-1280 (2003)] QDs. The large numbers of electrons, and the rapid increase of carrier density upon increasing the gate voltage, create a situation where the charge carrier scattering becomes dominant. This situation could hamper the observation of the quantised energy level in the transport measurement.

Since the signature of the band-filling is not that clear in the current epitaxially connected QDSLs, we performed a different approach to investigate the band-filling. Instead of observing the quantised level through a "dynamic" charge carrier via transfer characteristics measurement, we observed the band-filling occurrence through "static-charging" using EIS measurements. For example, the epitaxially-connected QDSLs with 8.1 nm diameter PbS QDs shows "static-charging" behaviour as shown in Fig. 3c and Supplementary Fig. S22c. All the samples with different QD diameters showed similar characteristics where a "peak-valley" can be observed. These occurrences were also observed for both the epitaxially-connected QD-SLs and the EDT-bridged QD assemblies.

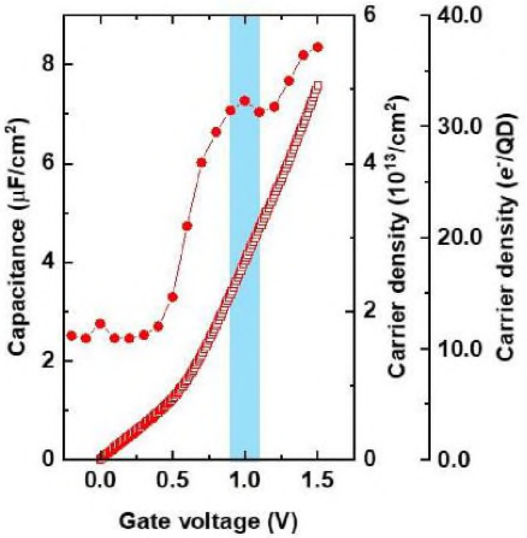

Figure R2 | The determination of charge carrier density from the measured capacitance using EIS measurement from epitaxially-connected PbS QD-SLs with 8.1 nm size.

To satisfy the reviewer's suggestion, we added some information related to the carrier density per QD in the Discussion section of the main text, paragraph 4, pages 14-15:

"... For semiconductor QD-SL that are epitaxially connected at touching radius *ρ*, the insulator-to-metal transition can occur at a critical carrier density value of $_{C}P^{3} \approx 0.3g$, where *g* is the number of degeneracies of conduction band minima.[12] In the case of lead-chalcogenide, including PbS, the number of state degeneracies is [4.62,63] In our epitaxially-connected QD-SL, constructed from 8.1 nm PbS QD, the epitaxy touching radius *ρ* was measured at around 2.3 nm (Supplementary Fig. 29). Consequently, the estimated critical carrier density value, *Tic*, to achieve the transition is around 2.1×10^{13} cm^{-2}. Considering the structure of the epitaxially-connected assembly with the given QD size, we estimated the QDs density as about 1.5×10^{12} dot cm^{-2}. Hence, this number of dots gives us the charge carrier density value of ~14 electrons/dot, sufficient to fill the lowest quantised energy state in the conduction band on PbS QDs. This value was reached in the present experiment by applying V_G of more than 1.0 V. Surpassing the theoretical value of the critical carrier density for the insulator-to-metal transition can explain the observation of the nearly temperature-independent conductivity when we applied a larger gate voltage. Notably, the carrier density obtained here is in the same order as the previous work on doped plasma-synthesised ZnO nanocrystals, where the insulator-to-metal transition was also observed.[10] As the gate voltage was applied above 1.5V, we obtained the carrier density accumulation for more than 30 electrons/dot, which means the transport on the higher quantised energy level was achieved. It also suggests that the electron transport in the QD-SL starts to become more delocalised on this energy level."

COMMENT #8:

Upon the very reducing gate potential, can the authors confirm that the system can still be reversibly brought back to a lower gate potential, and confirm that the effect is not polluted by the formation of metallic Pb?

Answer:

The reviewer is raising a crucial issue, and we realised its importance from the beginning. We confirmed that the system can be reversibly brought back to a lower gate potential and that the formation of metallic Pb did not pollute the observed effect. As shown in the following Fig. R3, we always measured I_D-V_G transfer characteristics after performing some temperature-dependent measurement sets before proceeding to other sets with higher applied gate voltage values. Performing I_D-V_G transfer characteristics in both forward and backward directions means we brought the system back to a lower gate voltage. As observed, the device still worked as a typical field-effect transistor with adequately high on/off ratio values and with very low off-current (see the logarithmic plots), even after the temperature-dependent measurement set with applied V_G of 2.6 V. The degradation of the current level after applying high gate voltage suggests that the observed effect is not originated from the formation of metallic Pb. If the metallic Pb pollutes the sample, the current should increase much without showing gate-dependent conductance.

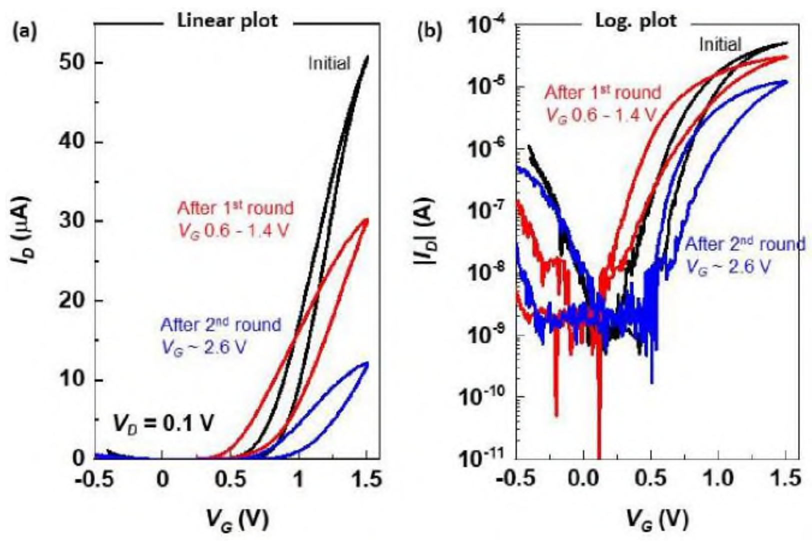

Figure R3 | Transfer characteristics ID-VG of 4T-EDLT devices. Before and after temperature-dependent conductance measurement. The left and right figure show linear and logarithmic plot, respectively.

COMMENT #9:

Line 215: "the observed size-dependent bandgap values probed by the EDLTs (Fig. S17) and excitonic peak in their absorption spectra confirmed that quantum confinement nature is preserved (Fig. S18" I would comment that, while it is true that quantum confinement is preserved, the authors are not confirming 3D confinement (but 2D is left). Second, I am 100% certain that the "bandgap values" cannot be obtained from EDLT in the way described in the SI. This refers to Fig S17: This figure purports to show that the gap of the quantum dots can be extracted by simply taking a linear line going to zero and match its slope approximately to the slope of the transport curve. Prior work showed that this is totally misleading because, on a log scale, there is no break at all. The same data should indeed be plotted on a log scale, and the authors will see that both sides of the transconductance vs reference are simple Arrhenius lines with a 1/kT slope, and that there is no "threshold" and no information retrievable about the gap. In fact, the only way to get an information on the gap is to actually extract the density of states, and this should give a bigger gap including the Coulomb charging energy. This error was previously made by the last author.

Answer:

Thank you for the comment from the reviewer. The excitonic peak in the absorption spectra of the epitaxially-connected QDs assembly has been observed in this work, and several prior reports on a quasi-2D (monolayer) [*Adv. Mater.* 30, 1802265 (2018)] and 3D (multilayer) system [*Adv. Mater.,* DOI:10.1002/adma.202207364]. The observation of the absorption signature, with clear peaks and valleys, of the QDSL monolayer reported in this work confirms that the original energy level separation is still preserved, although the QDs create atomic connections among them. Also, the absorption spectra show that the excitonic peak values are still size-dependent, suggesting the energy bandgap values of the epitaxially-connected QDSLs are also size-dependent.

The values of the energy bandgap of our QDSLs (QD diameters from 4.5 nm to 10.4 nm) are narrow enough to be observable under the ionic-liquid gating, in a similar fashion of the utilisation of a similar technique in many other material systems (including carbon nanotubes, organic semiconductors and various 2D materials) [*Adv. Funct. Mater.* 24, 33053311 (2014); *Adv. Mater.* 24, 4392-4397 (2014); *Nature Rev. Phys.* 3(7), 508-519 (2021)]. The applied gate voltage in ionic-liquid gate FET (Δ) will shift both the chemical potential (Δ) and electrostatic potential (Δ) as described in the following formula:

$$\Delta = \Delta + \Delta = \Delta + \frac{{}^{2}\Delta}{}$$

When the chemical potential (Fermi energy) of the semiconductor is within the bandgap, and the geometrical capacitance (C_G) of ionic liquid is adequately large, the second

term on the above formula can be ignored since the accumulation of charge carrier Δ inside bandgap is very small (related to any non-intrinsic available states inside the bandgap, which usually trap related). This condition can be determined by evaluating the subthreshold swing (*SS*) from the logarithmic plot of the transfer curve, which can be found in Supplementary Figs. 15 and 18 for the epitaxially-connected PbS QD-SLs and the EDT-bridged PbS QD assemblies, respectively.

We could notice the difference in *SS* characteristics between those two systems. The epitaxially-connected QDSLs exhibited a relatively constant *SS* value for all the QD diameters, around 66 mV/dec (Supplementary Fig. 15), almost achieving an ideal limit of ~60 mV/dec at room temperature. On the other hand, the EDT-bridged QD assemblies showed increased *SS* values (Supplementary Fig. 18). The small values of *SS* in epitaxially-connected PbS QDSLs suggest a cleaner state inside the bandgap. Therefore, the suppression of the second term in the above formula can be applied to the epitaxially-connected PbS QDSL system. Hence, a fair comparison of the threshold onsets can be performed for QD SLs with different QD diameters derived from the linear plot analysis (and must be from the linear plot). They would reflect the estimation of the band-edge. Such a case will not be reliably found for EDT-bridged QD assemblies.

Response to Reviewer 3:

COMMENT #1:

The manuscript by Bisri and coauthors reports the observation of metallic transport from epitaxially connected PbS quantum dot (QD) assemblies. QDs with intriguing quantum mechanical characteristics have been exploited for optoelectronic device applications (especially the light-emission or light absorption devices). Although significant progress has been made in field (even commercial products based on QDs are available), the electrical transport characteristics of QDs, through the assemblies of the materials, remain interior, which is known to be limited by inefficient hopping transport steps of charge carriers between the physically separated semiconductor crystals. This may serve as one of critical bottlenecks hampering the enhancement of device performances. The authors claim that by forming epitaxially connected superlattice of QDs (that can minimise orientational disorder among the QDs and increase the electronic overlapping between the neighbouring localised states) and gating the superlattice with electrolyte (that can induce extremely high density of charge carriers to fill the sub-states impeding transport), metallic transport behaviour is observed at high carrier density condition. This is a very important achievement in the field, and the manuscript is well-written. However, due to the following concerns, I cannot suggest its publication to Nature Communications at this point. If they are addressed carefully, I believe the results have good potential for publication in Nature Communications.

Answer:

We thank the reviewer summary of our current work, which exactly shows our motivation in elaborating on the electrical transport of colloidal semiconducting QDs. We agree with the reviewer's statement that the work related to electronic transport in QDs is the field's most crucial aspect for now. Understanding the mechanism of transport and improving the electrical performance of QDs-based devices are the key to establishing the next generation of electronic devices. We appreciate the suggestions to improve our manuscript by addressing the following comments carefully.

COMMENT#2:

Typically, metallic transport (through delocalised electronic states limited by scattering) is signatured with negative conductivity vs. temperature correlation, whereas insulator transport (through series of localised states assisted by thermal energy) follows positive conductivity vs. temperature correlation. The authors clearly showed that the highly negative

conductivity vs. temperature correlation at low carrier density conditions (small VGs) gradually approaches to a smaller value (close to zero) by inducing high carrier density (larger VGs) to the epitaxially-connected PbS QD superlattices. However, I am not fully convinced if the metallic transport is attained as the conductivity at the highest carrier density condition follows rather temperature-independent characteristics (or zero conductivity vs. temperature correlation), and explanation is needed.

Answer:

Thank you for the comment. Indeed, the metallicity and insulator behaviour can be distinguished by temperature-dependent conductivity, where the metallic transport shows a negative conductivity gradient versus temperature. What we have achieved is the almost temperature-independent conductivity. In this sense, we understand your criticism. The word of "Enabling" in the title is to show that we are on the boundary to realise the metallicity in QDs, while great efforts still must be carried out to achieve the metallic state.

In this manuscript, we have shown that the highest conductivity we have found is larger than the quantum conductance (e^2/h = 39 μS). The mobility is basically independent of the QD size. These are clear distinct from the insulating behaviour that has been reported in many references. Also, we provided a comprehensive discussion on this matter (Discussion sub-section, paragraph 4, page 14), along with the explanation and the possible origin of the transition. Based on all above, we believe that our present observation deserves being called metallic behaviour or insulator-to-metal transition.

COMMENT#3:

Also, metallic transport, the carrier mobility (different from conductivity) should be hampered by increasing charge carrier density (at low charge density conditions where insulator-type transport occurs through disordered states, increasing the charge carrier density helps filling the substates and thus can facilitate transport). The carrier density dependence of charge carrier mobility should also be confirmed.

Answer:

This comment is related to COMMENT#2. In the metallics state, the mobility should decrease with increasing of the carrier density. While in the semiconducting state, it increases with increasing the carrier density. Figure R1 shows the carrier density dependence of mobility. The mobility increases with the carrier density, indicating that our system is still in the semiconductor side, according to the definition of COMMENT#3. Despite this semiconductor-like behaviour, as explained in the reply to COMMENT#2, we claimed "Enabling metallic behaviour".

Since this matter is important, we have shown the example of the relationship between the charge carrier mobility and charge carrier density of 8.1 nm PbS QDs in Supplementary Fig. 22d.

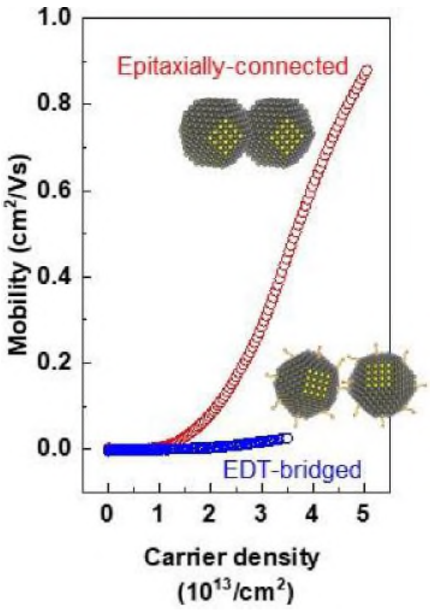

Figure R1 | Characteristics of electron mobility vs. charge carrier density accumulation. Comparison between epitaxially-connected PbS QD SLs and EDT-bridged PbS QDs assembly. The figure corresponds to Supplementary Fig. 22d in Supplementary information.

COMMENT#4:

I believe this part is the core finding of the manuscript and additional experiment to support the formation of the extended, delocalised state for transport at high carrier density conditions should be given. Here is my suggestion. Epitaxially-connected PbS QD superlattices exhibits insulator-type transport (thermally activated transport with positive conductivity vs. temperature correlation) at the low carrier density conditions, which are in their "attached-but-confined" (therefore localised) electronic state. At these conditions, the confinement of the electronic states was confirmed using UV-Vis-NIR absorbance measurement showing characteristic excitonic absorbance feature (Figure S18), which is good. At high carrier density conditions, the same superlattices now gated with electrolyte follow metallic transport indicating that the formation of delocalised state. This would now be "attached-and-delocalised" state - if I tweak the term the authors used. This should lead to apparent change in the electronic states of the superlattice, and the change should be observable through spectroelectrochemical measurement (monitor the UV-Vis-NIR spectrum while applying potentials to the ionic liquid gated devices). See Science 2003 (10.1126/science.1084424). The

in-situ measurement should be readily done from the combinations of the setup the authors already have (spectroscopy and electrical input/readouts).

Answer:

We appreciate the reviewer for the suggestion to perform spectroelectrochemistry measurements. Indeed, spectroelectrochemistry measurement is quite a powerful means. However, what we should point out regarding this issue are these two aspects:

1. One is the preservation of the quantised state in the undoped state of the epitaxially connected QDs.
2. The second is to clarify if the quantised state is still preserved in the highly doped state.

The first point is proved by the exciton peak in the undoped state of the epitaxially connected system, as shown in Supplementary Figure 21. Therefore, we called them “attached-but-confined”. Notably, “confined” means that the quantised level still survives. It is not appropriate to call them “localised”.

The second is the preservation of the quantised state even in the high carrier doping state. In the low carrier density state, the carrier transport is strongly localised. In contrast, the carrier transport becomes highly delocalised in the highly doped state, as observed in the temperature-independent conductivity. The question is whether the quantised level is still preserved or not in such a highly doped state. In order to answer this question, electrochemical measurements, either EIS or cyclic voltammetry (CV) are known to be important [*J. Phys. Chem. Lett.* 9, 1384–1392 (2018), *J. Phys. Chem. Lett.* 11, 2303–2307 (2020)].

We indeed performed the EIS measurement to determine the capacitance, and we also observed the “peak-valley” behaviour, shown in Fig. 3c and Supplementary Fig. 22c. This feature strongly indicates the existence of a peak in the electronic density of states even in the highly doped metallic state. In other words, the delocalised transport or band-like transport through the quantised states is nearly developed, meaning the formation of the miniband of the quantised state.

Figure R2 compares the gate-dependent capacitance/conductivity for EDT-capped and epitaxially connected systems. Both systems show peak behaviour in the capacitance, indicating that quantised levels exist in both systems. However, the respective localised and delocalised conductivity in EDT-capped and epitaxially connected systems imply that the electronic states are very different. In the former EDT-capped assembly, there is no miniband formation. In contrast, in the epitaxially-connected QDSLs, the miniband is well established, resulting in metallic conductivity.

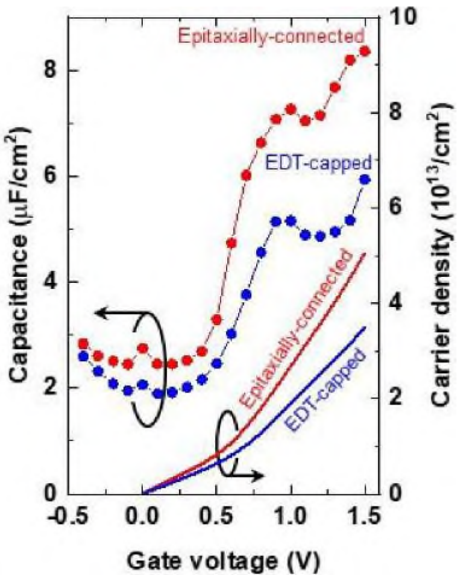

Figure R2 | Capacitance as a function of gate bias voltage and its corresponding estimated carrier density accumulation. This figure corresponds to Supplementary Fig. 22c in Supplementary information.

COMMENT#5:

Another concern is about the influence of QD size on transport. It was found that the size of QDs constructing the epitaxially-connected superlattice does not affect the transport characteristics. This is different from previous results (as already stated in the manuscript). The author should explain the why the difference occurs for the epitaxially connected PbS QDs (compared to EDT-bridged ones).

Answer:

We appreciate the reviewer's question regarding the size-dependency and independency of transport characteristics on different QDs assemblies (ligand-bridged vs epitaxially connected). This issue is one of the most important messages we deliver in our manuscript.

We have made an extended explanation about the ligand (EDT)-bridged QDs in the manuscript, specifically in the 1st paragraph of the Discussion section (page 13). Following the reviewer's suggestion, we rearranged the text and provided a more straightforward explanation so it could be easily understood. Therefore we modified the following discussion in the main text to become:

"The most striking difference in the electron transport properties between the EDT-bridged PbS QD assemblies and the epitaxially-connected QD-SLs is the size dependency of the electron mobility values (Fig. 3d). In the EDT-bridged PbS assembly, the mobility decreases

with increasing their size. The current understanding of such size-dependent mobility is the phonon-assisted hopping transport model, [55,] where the electronic hopping between the QDs is assisted by phonons. However, this hopping should get weaker as the diameter of the QDs increases due to the smaller wavefunction overlap.[54] This is because the spillover of the wavefunction is smaller when the QD diameter increases. In sharp contrast, the constant electron mobility value in the epitaxially-connected QD-SLs against the QD diameter strongly indicates that the hopping transport mechanism no longer governs their charge carrier transport process."

As for the epitaxially-connected QDs, the abovementioned hopping model is no longer suitable because it shows size-independent mobility behaviour that might indicate a distinct transport mechanism from the EDT-bridged counterpart. The model that can explain our condition is established by ref. 12 [*Nature Mater.* 15, 299–303 (2016)]. The comprehensive discussion on the epitaxially-connected PbS QDs is provided in the main text (subsection Discussion, paragraphs 3-4, pages 14-15) based on our understanding of the model.

COMMENT#6:

In fact, the size-independent mobility values shown in Figure 3d is confusing. Clearly, the current levels of the transfer characteristics shown in Figure 3b and the output characteristics in Figure S13 are all different or size-dependent. How would the mobility values extracted from these size-dependent raw data be size-independent. This seems counterintuitive.

Answer:

We appreciate the reviewer for pointing out this issue and apologise that the previous representation might confuse the reviewer. The way we displayed the output characteristics in Supplementary Fig. 14 (formerly Fig. S13) may be the origin of the confusion since both the *p*-channel and *n*-channel characteristics were plotted side-by-side. The y-axis for the *p*-channel characteristics might drive confusion since they were of different axis ranges. Meanwhile, the *n*-channel *y*-axes (right side) were of comparable orders for different QD diameters. We changed our representation of Supplementary Fig. 14 and Supplementary Fig. 17 (formerly Supplementary Fig. 16) to replot also the linear regime of the *n*-channel characteristics in separate panels to prevent any possible confusion. Importantly, we show them with a comparable y-axis range.

These *n*-channel output characteristics obtained from epitaxially-connected QDSLs with different QD diameters show comparable current levels (Supplementary Fig. 14). Furthermore, the charge carrier mobility values were extracted from the I_D-V_G transfer characteristics at these linear regimes. The values shown in Fig. 3d were extracted from the

highest applied gate voltage in transfer characteristics measurement (V_G = 1.5 V). The reviewer might want to also refer to Supplementary Fig. 23 to observe the gate voltage dependency of the charge carrier mobility values.

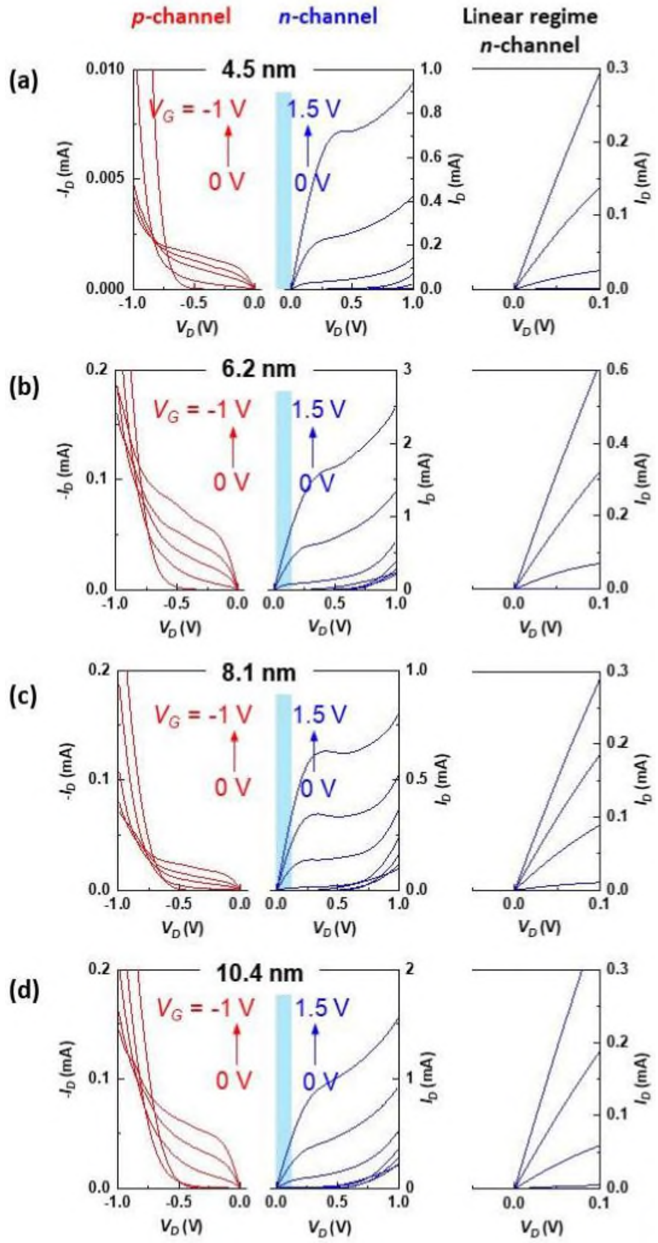

Supplementary Figure 14 | Output I_D-V_D characteristics of epitaxially-connected PbS QD-SLs ionic liquid gating FET with QDs size of (a) 4.5 nm, (b) 6.2 nm, (c) 8.1 nm, and (d) 10.4 nm. The right panel shows the correspond output characteristics in linear regime.

The most crucial aspect shown here is that the electron mobility in the epitaxially-connected QDSLs is constant within a similar order of magnitude. The *n*-channel I_D-V_D output characteristics and the I_D-V_G transfer characteristics of the epitaxially-connected QDSLs are

always within a similar order of magnitude for all different diameters of building blocks (Supplementary Fig. 14 and 15). It is reasonable to obtain nearly similar electron mobility values. In contrast, the EDT-bridged QD assemblies show different electron mobility values by orders of magnitude with increased QD size. It should translate to the difference between the current level in the I_D-V_D output characteristics and the I_D-V_G transfer characteristics by the orders of magnitudes when changing the QD diameters (Supplementary Figs. 17 and 18).

COMMENT#7:

Moreover, the authors have already confirmed that the contact resistance is a critical problem in the devices they tested. (Compare Figure 4b and 4c). If this is the case, the mobility value should be extracted with 4-probe measurement (instead of 2-probe measurement done in Figure 3b) to exclude any influence of the contacts in estimating mobility.

Answer:

We understand the reviewer's concern and would like to thank you for the suggestion. Indeed, we initially focused on a 2-terminal FET configuration for comparing the electronic performance between ligand-capped and epitaxially-connected PbS QDs assembly. The primary motivation is that the 2-terminal FET configuration is still the current reporting standard for comparing the transport characteristics in the colloidal QD field. Therefore, it would be beneficial in order to compare with the other results.

Indeed, we understand that contact resistance would be an issue for low-temperature measurements. Therefore, we also performed a 4-terminal FET configuration to diminish its influence and obtain its intrinsic properties. By diminishing the influence of contact resistance, the extracted mobility values can be one order of magnitude higher than those extracted from the 2-terminal FET configuration. It should be noted, however, that the reliability of comparing 4-terminal and 2-terminal FET configurations will also strongly depends on the channel quality and homogeneity. We also owed the good channel quality of the epitaxially-connected QDSLs.

We performed a 4-terminal FET measurement of all PbS epitaxially-connected QDSLs with all different diameters of QDs. We deduced the electron mobility values from such a configuration. Following the reviewer`s suggestion, in the revised manuscript, we also display the electron mobility values obtained from 4-terminal FET measurements in the size-dependent electron mobility plot (Figure 3d) for the epitaxially-connected QDSLs. Both the 4-terminal FET measurement results and the 2-terminal FET measurement results are consistent. We still observed the constant mobility values. Obviously, the mobility values extracted from the 4-terminal FET measurements are larger than those obtained from 2-terminal FET measurements since the influence of the contact resistance has been diminished.

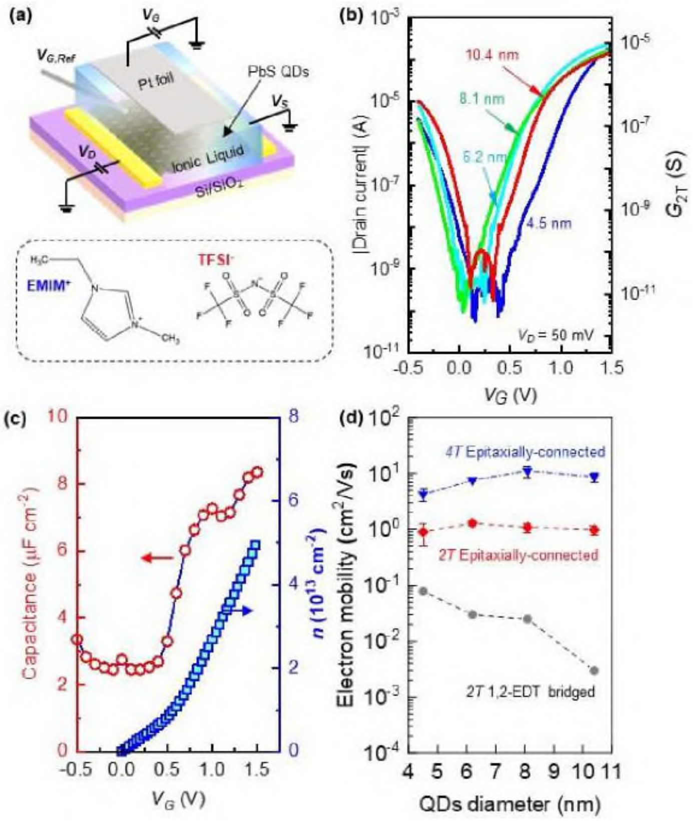

Figure 3 | Size-Independent Mobility in Electric-Double-Layer Transistors (EDLT) of PbS epitaxially-connected QD-SLs. (a) A schematic of the two-terminal EDLT device structure of the PbS QD-SL using EMIM-TFSI ionic liquid as electrolyte gate. **(b)** I_D-V_G transfer characteristics of the QD-SLs prepared from PbS QD with different diameters show ambipolar transport with a well-defined intrinsic region and a high on/off ratio for holes and electrons. **(c)** The gate-voltage-dependent capacitance value of the EDLT (red circle) and the corresponding accumulated sheet electron density (blue square) were extracted from potentiostatic electrochemical-impedance-spectroscopy (EIS) measurements. **(d)** Plots of the characteristic electron mobility values of the epitaxially-connected QD-SLs EDLT versus the diameter of the QDs (red diamond) showing size-independent values, in contrast to the conventional (decreasing) size-dependent electron mobility in the ligand-capped QD assemblies (i.e. EDT-capped PbS QD, black circle), the 4-terminal FET mobility of epitaxially-connected PbS QD-SLs (blue triangle) show the similar behaviour to the corresponding 2-terminal with higher mobility value.

COMMENT#8:

If the mobility and thereby the associated transport characteristics are size-independent, the temperature-dependence of the mobility for epitaxially-connected PbS QD superlattice should be also confirmed. They should be size-independent too. This needs to be definitely confirmed.

Answer:

Thank you for the reviewer's suggestion. We agree that measuring the temperature-dependent conductance for all sizes on epitaxially-connected PbS QD-SLs would be interesting. However, we cannot report them yet at this moment due to some practical technical issues. Measuring the low temperature with adequately high gate voltage is already challenging,

considering the stability of QDs assembly during the application of gate bias voltage, and a special experimental setup is required. This special setup, which is the built-in cryo-glovebox system, is currently under extensive maintenance since it is being moved to another institution following one of our co-authors. Hence, performing the complete measurement for all sizes of QDs at this moment is impossible. We will perform this more comprehensive research direction once the experimental setup is ready and improved.

Nevertheless, one of our hypotheses regarding the size-independency of low-temperature measurement in epitaxially-connected PbS QD SLs is as follows. Indeed, we might observe a similar mechanism (either Mott-VRH or ES-VRH) if we could cool down the temperature below 20K. However, in respect of achieving metallicity, they may behave differently. Carrier density will take precedence in determining whether the metallicity can be achieved or not. For the connected QDs, their connection width determines the critical carrier density value. The narrower the connection width, the higher the carrier density values need to be achieved. These connection width values are not only QD-diameter dependent but also facet-size-proportion-dependent and superlattice-angle-dependent. If we assume that, in general, the connection width is simply proportional to the diameter of the QDs (i.e. half of the diameter) (Supplementary Fig. 29), QD SLs of the smaller QDs would require much higher carrier density to be accumulated in order to achieve similar observation. The required carrier density would be beyond the limit achieved by using the current ionic liquid gate without destroying the QD assemblies due to device operation much beyond the electrochemical window. Nevertheless, this hypothesis must be proven and will be our next research subject.

COMMENT#9:

The description determining the hopping model from in Figure S21 is hardly convincing. The raw temperature dependent conductivity data can be fitted to different models following different temperature dependence. The authors determined that the transport follows the 3D Mott VRH model, simply based on the R^2 values of the fit. Even if the R^2 values for the fitting based on the 3D Mott VRH model shows the largest value among the series (Figure S21 d and h), all these values are in the range between 0.96 and 1.00 (the 2D VRH model fitting yields R^2 values between 0.99 and 1.00). Under this situation, I am not fully convinced if one can determine the transport model from the method based on R^2 values.

Answer:

We appreciate the reviewer for this comment. Indeed, this statistical significance issue for discussing the accurate transport mechanism is quite puzzling since all models fit

reasonably well with our observation. Taking this comment and also the other reviewers' comments into account, we have revised the corresponding part of the main text as follows.

Original paragraph:

"The controllability of the assembly formation and the fine-tuning of the carrier density by the ionic gating enable us to discuss the charge carrier transport in the QD assemblies. The high carrier density accumulation in the compact well-ordered monolayer of EDT-bridged QD assemblies shows that the transport mechanism strongly deviates from the nearest-neighbour hopping (NNH) Arrhenius-type behaviour. We then compared possible scenarios of different types of variable range hopping (VRH). Within the given temperature range, the transport process fits best with Mott 3D-VRH (Supplementary Fig. 24c) while largely deviating from Efros-Skhlovskii (ES) VRH ("$T^{-1/2}$). Due to the high carrier density accumulation by the ionic gating, there is no observable transition from NNH.[30] The observation of Mott 3D-VRH ("$T^{-1/4}$) indicates that significant disorder remains due to the non-coherence of the QD orientation in the EDT-bridged PbS QDs. However, they can achieve highly ordered assembly with uniform inter-QD distance."

After modification:

"The controllability of the assembly formation and the fine-tuning of the carrier density by the ionic gating enable us to discuss the charge carrier transport in the QD assemblies. The high carrier density accumulation in the compact and well-ordered monolayer of EDT-bridged QD assemblies shows that the transport mechanism strongly deviates from the nearest-neighbour hopping (NNH) Arrhenius-type behaviour, which is transforming to the variable range hopping (VRH). We then compared possible scenarios of different types of VRH. Within the given temperature range, the transport process fits best with Mott 3D-VRH (Supplementary Fig. 24c). However, we found that the fit also works well with Efros-Skhlovskii (ES) and Mott 2D-VRH and (Supplementary Figs. 24a and 24b, respectively). Rigorous identification of the hopping mechanisms should be made at lower temperatures, e.g., $T <$ 20K,[59] which was not covered in the current study and thus is left for a future study. The steeper temperature dependence and the Mott-VRH-like behaviour indicate that significant disorder remains due to the non-coherence of the QD orientation in the EDT-bridged PbS QDs, despite the uniform inter-QD distance."

COMMENT#10:

All the study in the manuscript was done by analysing the electron transport. PbS QDs follow ambipolar transport. Is there any reason why the study was done for electron transport only? Would there be different findings if the study was done based on hole transport?

Answer:

Thank you for the question from the reviewer. PbS QDs should intrinsically have ambipolar transport characteristics capability, where both electrons and holes can be transported. However, in a clean system with no significant electron traps, the electron conductance is much higher than the hole conductance, which can differ as much as 2 or 3 orders of magnitudes. Therefore, elucidating the electron transport in PbS QDs is fundamentally of a higher priority. Furthermore, many years of efforts have enabled us to minimise electron traps in PbS QDs, enabling reliable investigation of electron transport intrinsically.

In addition, positive gate modulation (to accumulate electrons) by ionic-liquid gating always works better than the negative gate modulation for Pb-chalcogenide QDs. It originated from the limitation of electrochemical windows of, primarily, $EMIM^+$ and $TFSI^-$. In particular, the electrochemical window of the hole accumulation side is typically reduced on the Pb-chalcogenide system than on standard Au or Pt. Therefore, attempts to accumulate holes by applying a similar voltage range for electron accumulation led to an electrochemical reaction.

Combining the above factors creates a hole accumulation (in negative gate bias) that is always lower than the electron accumulation. Nevertheless, selecting a suitable ionic liquid that can promote better (and stable) hole accumulation might pave ways to perform similar transport measurements like the electron side. We believe the same conclusion will be obtained by understanding the transport mechanism.

COMMENT#11:

If the superlattice is made of monolayer thick PbS QDs which with diameter of 4-10 nm, it means that the 4-10 nm film is the channel which is placed on top of 40 nm thick electrode (Au 30 nm and Ti 10 nm) in the given bottom contact device structure. In this case, is the electrode really the Au or is it the Ti? Also weren't there any issues with leakage current? (The gate current should be also plotted along with the transfer characteristics to clarify this concern).

Answer:

We thank the reviewer's comment. Yes, the film is formed by a monolayer assembly of QDs where the thickness is determined by the diameter of QDs itself. With this thickness (4

– 10 nm), the charge carrier might also be injected from the side of the Ti layer as well. However, it will not change our situation since Ti or even the possible formation of an oxide layer (TiOx) will also be a good electron injector to the PbS QD conduction level. Relatively good, nearly ohmic, contacts for electron injection are consistently demonstrated in 2-terminal FET at room temperature.

The crucial concern in any metal/QDs interface is the contact resistance that might appear between the QDs and electrode, regardless of which metal. Nevertheless, in this manuscript, we also showed that we could diminish this effect by changing our device configuration to 4-terminal FET.

We also would like to address the other concern of the reviewer. Although the assembly of QDs consists only of a monolayer, we did not observe any leak current (I_G) anomalies. I_G values are minimal during the gate voltage sweeping for transfer characteristics measurement, constant gate voltage application during output characteristic measurement, and low-temperature measurement. We provide additional information regarding this aspect by plotting the I_D-V_G transfer characteristics together with the corresponding I_G-V_G leak current both for the epitaxially-connected PbS QD-SLs (Supplementary Fig. 16) and EDT-bridged PbS QDs assembly (Supplementary Fig. 19). The minimal values of the I_G gate leak current, especially in the monolayer devices of the epitaxially-connected QDSLs, also suggest the high quality of the large-scale assembly coverage. This good coverage prevents the ionic liquid from leaking to the source or drain electrodes.

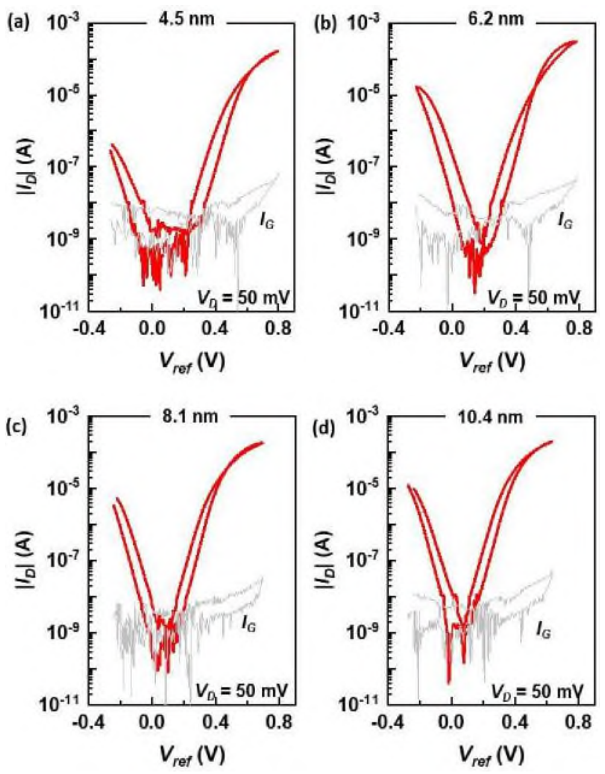

Supplementary Figure 16 | Logarithmic plot of transfer curve I_D-V_G and leak current I_G. The channel consists of epitaxially-connected PbS QD-SLs with diameter of (a) 4.5 nm, (b) 6.2 nm, (c) 8.1 nm, and (d) 10.4 nm.

COMMENT#12:

In page 6 and 7, the authors stated that the superlattice with uniform preferential orientation is on a large scale of 20 micrometers. Can the authors show SEM images of the 20 micrometer superlattice?

Answer:

Thank you for the comment pointing out the misleading statement regarding the area of observation under the SAED measurement. The micron-scale (20 i.im) written in the main text is an aperture diameter for choosing the observation area. It does not represent the entire area of observation. This size of the observed area is about 250 nm in diameter. Therefore, to prevent an ambiguous message, we revised the description in subsection "Epitaxially-Connected PbS QD Assemblies", paragraph 4 (page 7), from:

"...The sharp spots of the electron diffraction pattern obtained from the corresponding selected area electron diffraction (SAED) characterisation (**Fig. 1e**) also indicate the uniform preferential orientation of the atomic lattice in the epitaxially connected QD-SL on a larger scale (φ = 20 i.im)."

To become,

"...Figure 1e shows the selected area electron diffraction (SAED) pattern from the area of 250 nm in diameter. The sharp diffraction spots indicate the uniform preferential orientation of the atomic lattice over the scale of observation in the epitaxially connected QD-SL."

Furthermore, the arguments for large-area superlattice formation are also based on the results of GISAXS/GIWAXS measurements. The observation area (assembly exposed by the incident x-ray and scatter the x-ray) is in tens of micrometer scale. We obtained very sharp peaks from this large observation area, which are the signatures of atomic coherent and superlattice orientation.

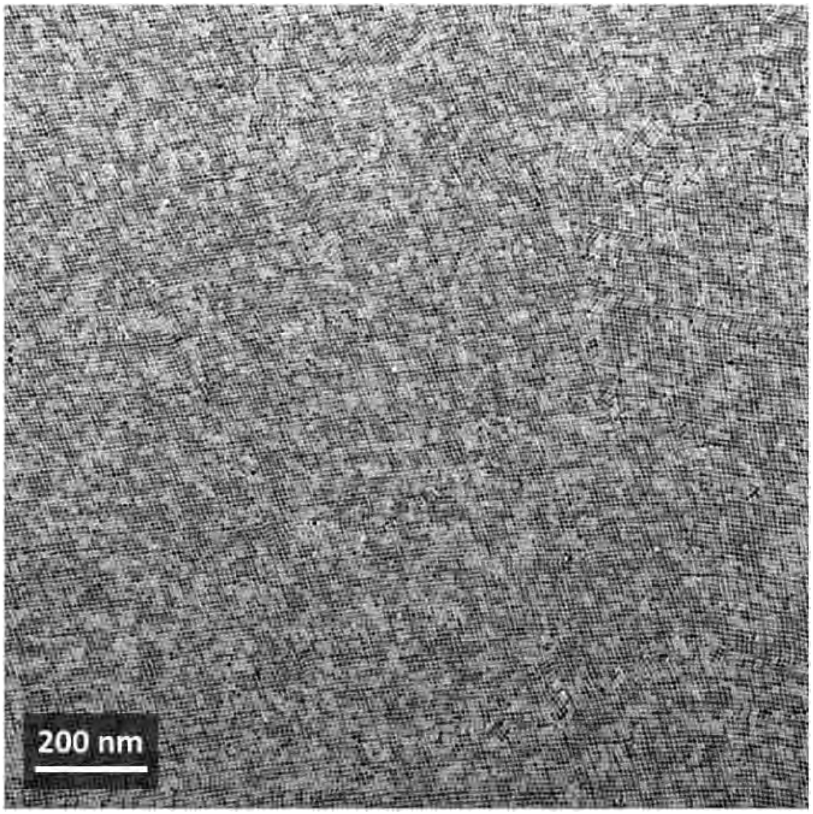

Figure R1 | Low-resolution TEM image with lower magnification (100,000×) of 8.1 nm epitaxially connected PbS QD SLs.

We also performed low-magnification TEM, as shown in figure R1 above. This magnification creates a 1.5 x 1.5 μm^2 observable frame. Any lower magnification, including by using SEM, would not be able to resolve the individual QD. At lower magnification, the number

of the QD within the imaging frame would become more than the number of available pixels in any imaging detectors.

To also qualitatively show the coverage of the QDSL monolayer on micrometer scale, we provide atomic force microscopy (AFM) images of the epitaxially-connected QDSL monolayers below as new Supplementary Figures.

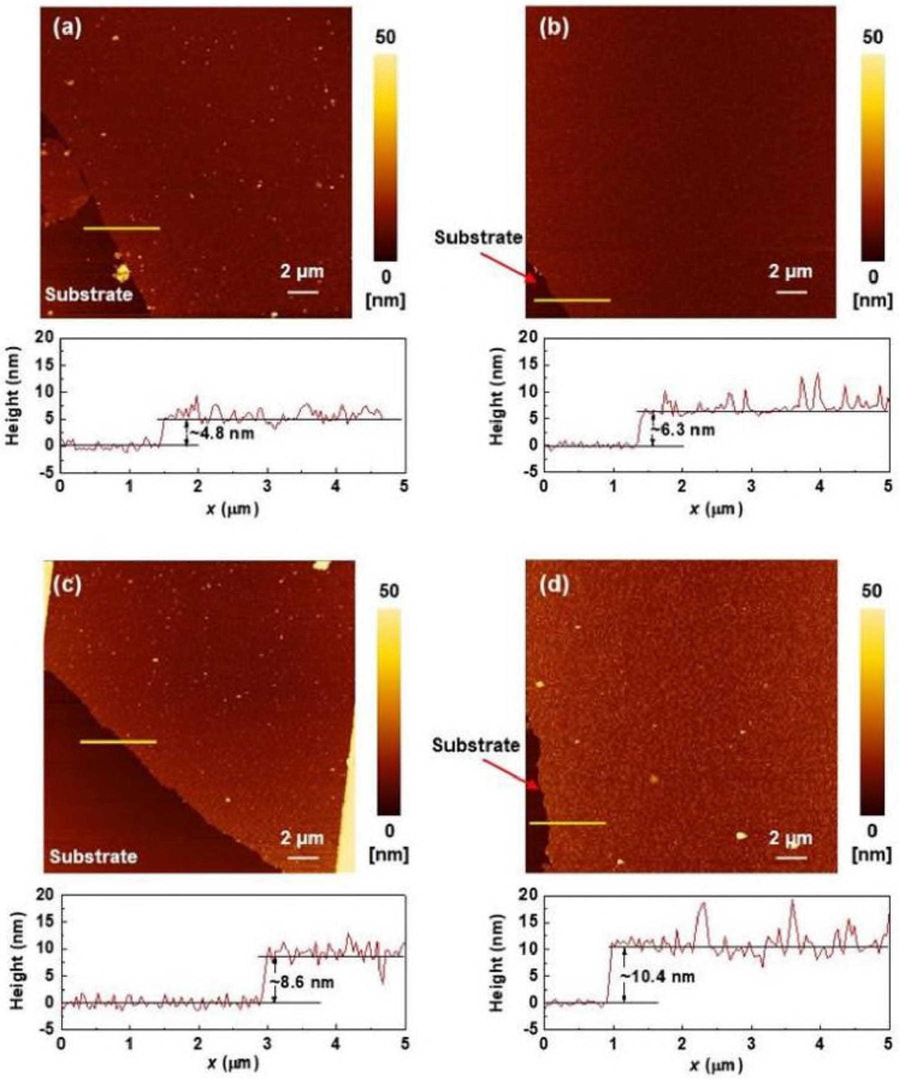

Supplementary Figure 6 | Atomic force microscopy (AFM) images of epitaxially-connected PbS QD-SLs. The AFM measurements capture monolayer assemblies built from **(a)** 4.5 nm, **(b)** 6.2 nm, **(c)** 8.1 nm, and **(d)** 10.4 nm diameters of PbS QDs. The corresponding thickness profile is provided below each image by referencing to the surface of the SiO_2 substrates.

The AFM images show good coverages within those 20 x 20 μm^2 scanning areas, which are comparable to the size the channel lengths of the devices, both the interdigitated 2-terminal FET and the 4-terminal FET (specifically, the distance between two voltage probes. Based on all these state-of-the-art morphological characterisations (AFM, TEM, and GISAXS/GIWAXS), we comprehensively analysed the actual conditions of the superlattices. The large-scale

coverages of the assemblies thus enable us to perform reliable electronic transport characterisations.

COMMENT#13:

Why was the 4-probe measurement done using side-gate EDLT? Is there a chance that the charge accumulation in the superlattice be different when using side-gate compared to using conventional top-gate?

Answer:

We thank you for the question. There should not be any difference between side gate and top gate configurations in terms of charge carrier density accumulation, as long as the gate electrode is larger than the QDs area. Our purpose in changing the structure from top-gate to side-gate devices is to accommodate the requirement of the cryostat setup that demands the compactness of the device layout on the substrate. While the two-terminal FET layout with a top gate can be accommodated, the additional number of electrodes in the four-terminal measurements created space restrictions. Using a side-gate structure can solve that.

COMMENT#14:

The capacitance value will be given from the equipment and this value would be divided by the area. I am curious how the authors determined the area for obtaining specific capacitance values.

Answer:

We appreciate the reviewer for the comment regarding specific capacitance values. To perform the capacitance measurement, we utilised the interdigitated device configurations since it has a sufficiently large area so that we could record the electrochemical impedance spectroscopy (EIS) signal well. The values of the capacitance (i.e. gate-dependent capacitance) were obtained from the analysis of the EIS measurement. Indeed, the capacitive area is one crucial parameter that needs to be considered. We defined the capacitive area for determining specific areal capacitance as the area of QDs assembly (including the electrode) covered by the ionic liquid, as shown inside the square with a dashed line below. We normally manually pattern the QD assembly into such a specific shape.

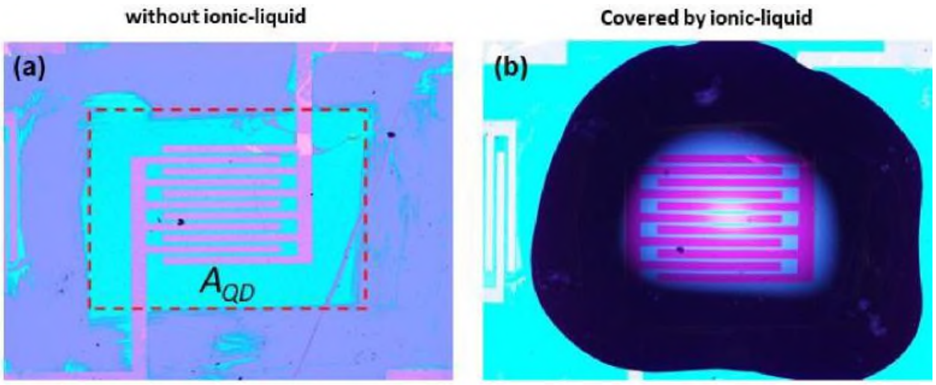

Figure R2 | Optical microscope image of device. (a) before and (b) after being covered by ionic-liquid. The area is defined as the area of ODs covered by ionic-liquid which is illustrated within the red-dashed line in (a).

The details of measurement and determination of capacitance value and charge carrier density can be found in the Supplementary Information (Supplementary Note 4 and Figure S19).

REVIEWER COMMENTS

Reviewer #1 (Remarks to the Author):

I reviewed the new version of this paper. It takes care of my comments and I recommend to publish it.

Reviewer #2 (Remarks to the Author):

What are the noteworthy results?

-The authors achieved metallic transport in an array of connected quantum dots at a high charge number. Prior work on disordered highly doped connected ZnO nanocrystals showed the same feature, but this realization is structurally ordered.

Will the work be of significance to the field and related fields?

-The work sets a standard for the general ongoing interest in the conductivity of quantum dot solids.

How does it compare to the established literature? If the work is not original, please provide relevant references.

-The work is original and appears to have been performed more carefully that recently work published claiming 300cm2/Vs mobility in similar system.

Does the work support th.e conclusions and claims, or is additional evidence needed?

-I think so.

Are there any flaws in the data analysis, interpretation and conclusions? Do these prohibit publication or require revision?

Is the methodology sound? Does the work meet the expected standards in your field?

-No obvious flaw.

Is there enough detail provided in the methods for the work to be reproduced?

-It is hard, but seems possible to reproduce.

Reviewer #3 (Remarks to the Author):

I appreciate the efforts put together by the authors in preparing the revised manuscript. Most of my comments were addressed carefully. However, the following issue still remains as a serious concern.

I view that the important findings and main contributions of this paper in the community is the fabrication of epitaxial attachment of QDs in forming large area superlattice experimentally, which permitted attaining metallic transport behavior through QD superlattice at high charge carrier density conditions. The revised manuscript at the current stage, however, does not fully support that these findings can be highlighted.

- Experimental methods in attaining the epitaxial attachment (although I deeply appreciate the efforts the in doing so) has been reported previously (Law et al Nature Commun. 2022, 13, 6802 for PbSe QDs, which I believe share many of the electrical/electronic/structural characteristics with PbS QDs).

- The claim of the metallic transport remains unclear; It is stated that the achievement is ON THE BOUNDARY and that what is shown is almost temperature-independent conductivity (which implies that it may eventually be negative temperature dependent conductivity, if metallic behavior is truly reached, but it is NOT attained at the current stage). These are somewhat vague statements.

- More importantly, I am skeptical if the statement is well supported by experimental results. The claim is primarily supported by the experimental observation of the size-independent transport characteristics shown in the new Figure 3d. The authors claimed that the results show “size-independent mobility values”even after the measurement excluded the contact effect. However, considering the error bar of the data shown, I view that size-dependence is there (yet moderate) . In fact, this moderate change (less than an order of magnitude) is just about the size-dependence in the mobility values reported previously (Law et al. Nano Lett. 2010, 10, 1960 for electrons in PbSe QDs. (It may be a coincidence, but interestingly, the size dependent mobility value peaked at a specific size for both PbSe QDs (6 nm) in the previous report and PbS QDs (8 nm) here). Even with such a moderate size-dependence observed at room temperature, studies showed that the transport characteristics differs drastically for QDs in different sizes, when temperature-dependent transport characteristics experiments were carried out (Wood et al. Nat. Comm. 2020. 11. 2852 for PbS QDs, Frisbie et al. Nano Lett. 2011, 11, 3887 for PbSe QDs, Sim et al. J. Phys. Chem. Lett. 2012, 3, 714).

- Unless the size-independence in transport is rigorously supported from experimental data, I can only hesitate in agreeing i) that mobility of the superlattices are QD-size independent ii) that it shows completely different behavior to what was observed previously, and iii) that therefore it deserves to be states as “enabling metallic transport through QDs.

Overall, I still consider that the experiment I suggested in the previous review must be carried out for the integrity of the work. As already mentioned by the authors, the size independent transport is the most critical experimental result that can support the authors statement of new finding.

For your note, I commented as follows “If the mobility and thereby the associated transport characteristics are truly size-independent, the temperature-dependence of the mobility for epitaxially-connected PbS QD superlattice should be also confirmed. They should be size-independent too. This needs to be definitely confirmed.”

Point-by-point responses to reviewers
NCOMMS-22-37189A
"Enabling Metallic Behaviour in Two-Dimensional Superlattice of Semiconductor Colloidal Quantum Dots"

Response to Reviewer 1:

COMMENT #1:

I reviewed the new version of this paper. It takes care of my comments and I recommend to publish it.

Answer:

We are delighted that the reviewer is satisfied with the revised manuscript and recommended the publication of this manuscript as it is in *Nature Communications*.

Response to Reviewer 2:

COMMENT #1:

What are the noteworthy results?
-The authors achieved metallic transport in an array of connected quantum dots at a high charge number. Prior work on disordered highly doped connected ZnO nanocrystals showed the same feature, but this realisation is structurally ordered.

Will the work be of significance to the field and related fields?
-The work sets a standard for the general ongoing interest in the conductivity of quantum dot solids.

How does it compare to the established literature? If the work is not original, please provide relevant references.
-The work is original and appears to have been performed more carefully that recently work published claiming 300 cm^2/Vs mobility in similar system.

Does the work support the conclusions and claims, or is additional evidence needed? -I think so.

Are there any flaws in the data analysis, interpretation and conclusions? Do these prohibit publication or require revision?
Is the methodology sound? Does the work meet the expected standards in your field?
-No obvious flaw.

Is there enough detail provided in the methods for the work to be reproduced? -It is hard, but seems possible to reproduce.

Answer:

We thank you for all the comments from the reviewers. We appreciate the reviewer's understanding of the challenges and merits of our current work.

Response to Reviewer 3:

COMMENT #1:

I appreciate the efforts put together by the authors in preparing the revised manuscript. Most of my comments were addressed carefully. However, the following issue still remains as a serious concern.

I view that the important findings and main contributions of this paper in the community is the fabrication of epitaxial attachment of QDs in forming large area superlattice experimentally, which permitted attaining metallic transport behaviour through QD superlattice at high charge carrier density conditions. The revised manuscript at the current stage, however, does not fully support that these findings can be highlighted.

- Experimental methods in attaining the epitaxial attachment (although I deeply appreciate the efforts in doing so) has been reported previously (Law et al Nature Commun. 2022, 13, 6802 for PbSe QDs, which I believe share many of the electrical/electronic/structural characteristics with PbS QDs).

Answer:

We appreciate the reviewer's comment. Indeed, the fabrication of epitaxially-connected QD superlattices has recently become one emerging avenue for investigation in the colloidal QD field. Notably, electrical characterisations of such assemblies are attractive. To make it possible, obtaining high-quality and large-scale QDSLs is vital. As highlighted by the other reviewer, it is still challenging and not trivial for many researchers. The mentioned publication focused on the STM/STS characterisation of the PbSe QDSLs, where the large-scale assembly is unimportant. We may share some steps of the fabrication methods.

In contrast, our manuscript emphasises the work on a large-scale monolayer assembly of epitaxially-connected PbS QDSLs where comprehensive structural and electrical characterisations were performed. It is for the first time that the influence of the QD diameter in the epitaxially-connected QDSLs system is investigated, with a particular focus on the relationship between QD size, orientational ordering, and their electrical transport properties.

COMMENT#2:

- The claim of the metallic transport remains unclear; It is stated that the achievement is ON THE BOUNDARY and that what is shown is almost temperature-independent conductivity (which implies that it may eventually be negative temperature dependent conductivity, if

metallic behavior is truly reached, but it is NOT attained at the current stage). These are somewhat vague statements.

Answer:

We understand the reviewer's concern regarding this point. Negative temperature-dependent conductivity is among the criteria of metallic transport but is not the only one. Before achieving negative temperature-dependent conductivity, one should realise a system where the conductivity value indeed surpasses the quantum conductance limit as proof of the charge carrier delocalisation. In addition to that, for epitaxially-connected QDs or nanocrystals, a theoretical framework has been established that stipulates the critical carrier density to achieve metallic behaviour [*Nature Mater* 15, 299–303 (2016)], in which the epitaxial connection cross-section and the number of electronic degeneracies of the compounds are the crucial parameters. This critical carrier density should be surpassed.

Almost similar insulator-to-metal transition occurrence has recently been reported for highly-doped plasma-grown ZnO nanocrystal networks [*Nano Lett.* 17, 4634–4642 (2017); *Sci. Adv.* 5, eaaw1462 (2019)]. In this report, the temperature-dependent conductivity also only reached a condition where it was temperature-independent in the metallic region. The achievement of quantum conductance is the crucial key to metallic transport, although only temperature-independent conductance was demonstrated. To date, this phenomenon has never been demonstrated for the colloidal QD system. Thus, we believe that our system reached the level which is worth being called metallic transport.

COMMENT#3:

- More importantly, I am skeptical if the statement is well supported by experimental results. The claim is primarily supported by the experimental observation of the size-independent transport characteristics shown in the new Figure 3d. The authors claimed that the results show "size-independent mobility values" even after the measurement excluded the contact effect. However, considering the error bar of the data shown, I view that size-dependence is there (yet moderate). In fact, this moderate change (less than an order of magnitude) is just about the size-dependence in the mobility values reported previously (Law et al. Nano Lett. 2010, 10, 1960 for electrons in PbSe QDs). It may be a coincidence, but interestingly, the size dependent mobility value peaked at a specific size for both PbSe QDs (6 nm) in the previous report and PbS QDs (8 nm) here. Even with such a moderate size-dependence observed at room temperature, studies showed that the transport characteristics differs drastically for QDs in different sizes, when temperature-dependent transport characteristics experiments were carried out (Wood et al. Nat. Comm. 2020. 11. 2852 for PbS QDs, Frisbie et al. Nano Lett. 2011, 11, 3887 for PbSe QDs, Sim et al. J. Phys. Chem. Lett. 2012, 3, 714).

Answer:

We understand the reviewer's concern regarding the QD-size-dependency of the charge carrier mobility values. We should emphasise that the mobility values reported in this manuscript were deduced by considering the gate-voltage-dependent charge carrier density values through measurements. Supplementary Fig. 23a-b shows the influence of the charge carrier density (induced by the gate voltage) and the QD size on the electron mobility values for both the EDT-bridged QD assemblies and the epitaxially-connected QD-SLs. At low carrier density ($V_G < 1$ V), we observed the increase of electron mobility by the diameter (from 4.5 nm to 6 nm), before decreasing again at a larger diameter (> 6 nm) (Supplementary Fig. 23a). To some extent, it is potentially comparable to what was observed in the old reports of disordered (non-monolayer) QD assembly films highlighted by the reviewer [Law et al. *Nano Lett.* 10, 1960 (2010); (and its derivative) *J. Phys. Chem. Lett.* 3, 714 (2012); and *Nano Lett.* 11, 3887 (2011)] where the QDs film is modulated by SiO_2 gated.

However, once the carrier density increases (applying higher gate voltage), the mobilities decrease as the QD size increases on the EDT-bridged QDs assembly case. <u>It should be noted that the decrease (or the change) of mobility by size is on the order of magnitude</u>. It is consistent with the phonon-assisted hopping transport model [Wood et al. *Nature Comm.* 11, 2852 (2020)] and many other recent observations in intrinsic systems (spectroscopy studies) [e.g. *Nano Lett.* 17, 893 (2017)]. Generally speaking, the effect of extrinsic effects, such as carrier traps, dominates the transport mechanism in the low carrier-density regions. In contrast, the trap effect is less effective in the high carrier density region, and intrinsic effects such as phonon scattering become more dominant.

Notably, the electron mobility values in the epitaxially-connected QD-SLs showed size-independent behaviour once they entered their more intrinsic condition ($V_G > 0.6$ V) (Fig. Supplementary S23b). The observation of the size-independent mobility of the epitaxially-connected PbS QD-SLs at room temperature, in the more intrinsic condition, is the manifestation where the transport mechanism should be completely different from those occurring in the ligand-bridged (EDT-bridged) QDs assemblies. By performing temperature-dependent conductivity measurements, the difference becomes more evident.

<u>**COMMENT#4:**</u>

- Unless the size-independence in transport is rigorously supported from experimental data, I can only hesitate in agreeing i) that mobility of the superlattices are QD-size independent ii) that it shows completely different behaviour to what was observed previously, and iii) that therefore it deserves to be states as "enabling metallic transport through QDs.

Answer:

We believe the provided experimental data strongly support our conclusions that the size-dependent mobility is very weak in epitaxially-connected QDs, in sharp contrast to EDT-bridged assemblies. Indeed, the room temperature mobility does not precisely show constant value over the various size of QDs. However, the variation of the mobilities value is minimal, less than one order of magnitude. It is totally different from the mobility trends of the conventional ligand-capped counterparts and from the references that the reviewer pointed out in the above comments.

Following the reviewer's comment on point (i), we rephrased the "constant or size-independent electron mobility value" to become "mobility trend with a very weak size dependency" to avoid any possible ambiguity or misunderstanding. For example, in the Discussion section, paragraph 1, page 14:

"In sharp contrast, the constant electron mobility value in the epitaxially-connected QD-SLs against the QD diameter strongly indicates that the hopping transport mechanism no longer governs their charge carrier transport process."

to become

"In sharp contrast, the electron mobility values in the epitaxially-connected QD-SLs show weak dependency against the QD diameter. The differences in the mobility values are within a similar order of magnitude. This observation suggests that the hopping transport mechanism no longer governs their charge carrier transport process."

Furthermore, addressing points (ii) and (iii), we emphasise that simultaneous observation of weakly size-dependent mobility and *T*-independent conductivity above the quantum conductance is entirely a new finding. These findings thus led us to conclude the observation of metallic transport enabled by field-induced doping of the electric double-layer gating.

COMMENT#5:

Overall, I still consider that the experiment I suggested in the previous review must be carried out for the integrity of the work. As already mentioned by the authors, the size independent transport is the most critical experimental result that can support the authors statement of new finding.

For your note, I commented as follows "If the mobility and thereby the associated transport characteristics are truly size-independent, the temperature-dependence of the mobility for epitaxially-connected PbS QD superlattice should be also confirmed. They should be size-independent too. This needs to be definitely confirmed."

Answer:

We understand the reviewer standing regarding this matter. Nevertheless, the absence or the existence of the suggested additional experiments will not affect the integrity of this work. All the assertions have been supported by the currently available experimental evidence. Furthermore, as mentioned by the other reviewers, our comprehensive and careful approach can be considered the current "golden standard" in performing electrical characterisations of colloidal QD devices and the relevant structural correlations.

As we explained, including our previous responses, the observation of insulator-to-metallic transition (IMT) in the epitaxially-connected QD-SLs is valid and consistent with the best available theoretical framework for observing the metallic transport (associated with band-like transport) in QDs (i.e. by Skhlovskii et al.). The most essential prerequisite is to surpass the critical carrier density and achieve quantum conductance.

As explained in the main text and the responses to reviewers, this paper aims to fabricate films with large-scale structural coherence and to elucidate their relation to transport properties. The systematic investigation of size dependence of low-temperature transport is a future issue. We anticipate that the low-temperature transport is size dependent because the criteria for the metallic transport should be size-dependent since the corresponding critical carrier density is determined by the connection width (strongly related to the QDs size itself). Since this additional effect could make our claim too complicated, we decided not to go into the size-dependency of the low-temperature transport in this paper.
